# Using a ResearchKit Smartphone App to Collect Rheumatoid Arthritis Symptoms From Real-World Participants: Feasibility Study

**DOI:** 10.2196/mhealth.9656

**Published:** 2018-09-13

**Authors:** Michelle Crouthamel, Emilia Quattrocchi, Sarah Watts, Sherry Wang, Pamela Berry, Luis Garcia-Gancedo, Valentin Hamy, Rachel E Williams

**Affiliations:** 1 GlaxoSmithKline Collegeville, PA United States; 2 GlaxoSmithKline Stockley Park Uxbridge United Kingdom; 3 GlaxoSmithKline Stevenage United Kingdom

**Keywords:** rheumatoid arthritis, smartphone, mobile phone, patient-reported outcome measures, mobile applications

## Abstract

**Background:**

Using smartphones to enroll, obtain consent, and gather self-reported data from patients has the potential to enhance our understanding of disease burden and quantify physiological impact in the real world. It may also be possible to harness integral smartphone sensors to facilitate remote collection of clinically relevant data.

**Objective:**

We conducted the Patient Rheumatoid Arthritis Data From the Real World (PARADE) observational study using a customized ResearchKit app with a bring-your-own-device approach. Our objective was to assess the feasibility of using an entirely digital approach (social media and smartphone app) to conduct a real-world observational study of patients with rheumatoid arthritis.

**Methods:**

We conducted this observational study using a customized ResearchKit app with a bring-your-own-device approach. To recruit patients, the PARADE app, designed to guide patients through a series of tasks, was publicized via social media platforms and made available for patients in the United States to download from the Apple App Store. We collected patient-reported data, such as medical history, rheumatoid arthritis-related medications (past and present), and a range of patient-reported outcome measures. We included in the assessment a joint-pain map and a novel objective assessment of wrist range of movement, measured by the smartphone-embedded gyroscope and accelerometer.

**Results:**

Within 1 month of recruitment via social media campaigns, 399 participants self-enrolled, self-consented, and provided complete demographic data. Joint pain was the most frequently reported rheumatoid arthritis symptom to bother study participants (344/393, 87.5%). Severe patient-reported wrist pain appeared to be inversely linked with the range of wrist movement measured objectively by the app. At study entry, 292 of 399 participants (73.2%) indicated a preference for participating in a mobile app–based study. The number of participants in the study declined to 45 of 399 (11.3%) at week 12.

**Conclusions:**

Despite the declining number of participants over time, the combination of social media and smartphone app with sensor integration was a feasible and cost-effective approach for the collection of patient-reported data in rheumatoid arthritis. Integral sensors within smartphones can be harnessed to provide novel end points, and the novel wrist range of movement test warrants further clinical validation.

## Introduction

### The Need for Novel Data Collection Methods

Traditionally, clinical research and data collection is primarily conducted through a site-based approach. Patients are screened and recruited by clinical research centers, followed by a series of clinical visits scheduled for procedures and clinical assessments. Other methods include data collection via telephone, mail, or electronic surveys. These site-based approaches can be complex, costly, and time consuming; limitations associated with these approaches include investigator bias [1], parking lot syndrome [2], and white coat syndrome [3]. Since health status involves multiple dimensions and is a continuum, clinicians’ assessments during infrequent clinical visits may not be sufficient to fully evaluate health status. Novel data collection methods to capture continuous data from the patient perspective are needed.

Mobile phone ownership is becoming more widespread globally, with the number of people using mobile phones in 2017 estimated to be 4.8 billion worldwide [4]. Following the decline in the popularity of other forms of communication (postal mail and landline phones) the mobile phone has recently emerged as a potentially useful and successful technology for health measurement and health management [5-7], a clear shift that appears to be welcomed by patients [8]. When paired with specialized apps, designed to provide standardization of the captured data, mobile phones may be easily used to both engage the patient with their treatment and provide a conduit through which patient-reported data can be gathered and transmitted [9]. The use of smartphones to collect real-world data directly from patients has been shown to be cost effective and fast, and, importantly, it empowers the patients [5,7,10]. Although smartphones have been shown to be effective at gathering data, high rates of attrition have been reported in studies using smartphones alone [5,11,12], highlighting a need to improve and optimize current methods of promoting participant retention.

### Rheumatoid Arthritis as a Case Study

Patients with long-term chronic and physically disabling conditions, such as rheumatoid arthritis (RA), who need to manage daily care activities and fluctuations in their RA symptoms at home, may be a population for whom electronic data collection could be of particular use. RA is a chronic, progressive autoimmune disease affecting the mobile joints of the body, which may result in substantial and irreversible disability; symptoms include joint pain or tenderness, joint swelling, morning stiffness, reduction in joint range of movement (ROM), muscle pain, and fatigue [9,13]. Various chronic and progressive autoimmune conditions, including RA, are typically characterized by periodic disease flares followed by periods of relative quiescence that are unpredictable. Assessment of disease status during prescheduled physician visits, whether for routine purposes or for gathering data for clinical research purposes, can easily miss these periods of disease exacerbation, leading to an incorrect interpretation of the patient’s disease status. The use of smartphone apps in clinical research means that medical data can be efficiently collected at a greater number of time points, compared with more traditional research methods [12]. Thus, the ability of the patient to self-report data relating to the subjective signs and symptoms of their disease may pave the way for more holistic clinical research datasets, able to better capture the experience of patients living with chronic conditions, and may provide immediate feedback to their health care providers, ultimately improving clinical benefit overall [11].

### Data Capture Using ResearchKit

ResearchKit (Apple Inc, Cupertino, CA, USA) is an iOS-based, open source framework for mobile medical research released in 2015. It is an app composed of preconstructed modules, which can be adapted according to the research requirements. To date, ResearchKit has been used in observational, real-world studies of cardiovascular health, asthma, Parkinson disease, type 2 diabetes, and cancer [5,7,11,12]. So far, to our knowledge, it has not been used to conduct an entire scientific research study in patients with RA enrolled remotely, including obtaining patients’ consent and data capture.

Several observational studies have assessed the use of smartphones to measure subjective RA disease activity through adoption of validated questionnaire-based patient-reported outcome (PRO) measures [14-16]. Additionally, creative use of the integrated sensor platforms present within most smartphones could facilitate collection of a large number of objective variables relevant to RA. For example, monitoring the impact of physical activity on cardiovascular health, by gathering objective data relating to patient mobility and activity levels through the use of the integral smartphone global positioning system, was shown to be successful [12]. An attempt to capture data relating to gait analysis in patients with RA has been reported, but this method awaits full validation [15,17].

### Objectives

The Patient Rheumatoid Arthritis Data From the Real World (PARADE) study was a siteless, prospective, real-world observational study in which patients with RA could self-recruit, provide consent, enroll, and report their medical data entirely via a customized ResearchKit app downloaded to their own smartphone. The key objectives were to assess the feasibility of using ResearchKit to enroll patients into a study and of collecting patient data using this app, including subjective assessments (ie, self-reported symptoms, validated health-related quality of life surveys, and a joint-pain map), as well as a novel, objective wrist ROM test. To determine the feasibility of the app and siteless approach to recruitment, the aims of the study were to enroll a minimum of 300 participants in 1 month, demonstrate participant demographics similar to those of an existing RA registry, and evaluate algorithms developed to support the objective measurement of RA symptoms via the app.

## Methods

### App Design and Testing

The PARADE app was created and developed by GlaxoSmithKlein (GSK; Brentford, UK) and Possible Mobile (Denver, CO, USA) using the ResearchKit platform, which is open source and available on GitHub [18]. Two sets of user acceptance testing were performed during the app’s development. During user acceptance testing, users tested the software to ensure that it could handle required tasks in real-world scenarios. The first user acceptance testing was performed with 3 volunteers with RA who provided feedback on screen functionality and the electronic PROs. The second user acceptance testing was performed with study team members 1 month before the app launch as the final approval testing. Multimedia Appendix 1 provides screenshots from the app.

### Recruitment Process and Ethics

This study (study number 205718) was approved by the Quorum institutional review board (Quorum Review Inc, Seattle, WA, USA). The study was an observational platform pilot; it was not registered within the clinical trial registry. The app was made available in the United States via the Apple App Store. We identified California, Texas, New York, Pennsylvania, and Florida as the top 5 states with a high prevalence of RA; therefore, we launched targeted digital patient recruitment campaigns via social media platforms, such as Facebook (Facebook, Inc, Menlo Park, CA, USA) and Twitter (Twitter, Inc, San Francisco, CA, USA), in those states. We also targeted HealthUnlocked (HealthUnlocked, London, UK), Inspire (Inspire, Arlington, VA, USA), and users on Facebook who were followers of the Arthritis Foundation [19] and Creaky Joints [20] to increase awareness and drive interest to download the PARADE app. Prospective participants downloaded the app using their own App Store credentials and self-navigated through elements of the app, including a study video, eligibility screen, electronic informed consent screen, and data collection screen, via the smartphone touchscreen interface. The involvement of GSK was made clear on the Welcome screen of the app and in the informed consent process.

We set the recruitment time frame for 1 month with a target enrollment of 300 patients. The consenting process was compliant with Title 21 of the Code of Federal Regulations Part 11 (21CP11) to ensure that it was trustworthy, reliable, and equivalent to paper records according to the US Food and Drug Administration.

Once prospective participants had downloaded and opened the app, they were presented with information about the study and an inclusion and exclusion criteria questionnaire. Inclusion criteria were being 21 years of age or over, being English speaking, living in the United States, and having a physician’s diagnosis of RA. Participants who met the eligibility criteria proceeded to electronic informed consent. Once they completed this, they could receive a copy of the consent form by email for their records. Collection of personally identifiable information (including email address) was restricted to the consent process and was housed separately from the study data on secure servers (Medidata Solutions Inc, New York, NY, USA). Throughout, participants navigated the app using the smartphone touchscreen.

### Study Design

We conducted the study entirely via the app with no human interaction, medical intervention, or financial incentives.

Possible Mobile programmed the app to automatically randomly allocate participants, without stratification, into 2 groups with differing degrees of access provided to their personal data. Group A could access their personal symptom data dashboard every day, whereas group B could access it only at the end of the study after 12 weeks of data collection. The informed consent form notified participants that they would be assigned by chance into 1 of 2 study groups; however, participants were not informed about the purpose of the randomization.

### Assessments

Data captured at enrollment (week 1) were baseline demographics, health history information, retention, and medications. Participants were encouraged to complete several subjective and objective study tasks at different time points, as described below.

#### Subjective Study Tasks

Each week, participants received a reminder via the app to complete a variety of tasks accessed via their personalized dashboard and designed to evaluate their disease status. Weekly reported outcome measures were the Rheumatoid Arthritis Severity Scale (patient global assessment) [21] and a series of semiquantitative scales, including a pain scale, morning stiffness scale, and mood scale (not reported here). In addition, participants were encouraged to complete a weekly survey to monitor their satisfaction with the app. At weeks 1, 4, 8, and 12, participants were encouraged to complete additional validated PRO measures: a health status survey (5-level version of the EuroQoL, 5 dimensions) [22], a physical function assessment (Health Assessment Questionnaire-Disability Index) [23] and a fatigue scale (Functional Assessment of Chronic Illness Therapy-Fatigue) [24].

An interactive joint-pain map was designed specifically for the app to record the number and severity of painful joints (from 55 prespecified joints). At weeks 1 and 8, participants were asked to score the pain in each joint (joints were presented in a body map) as 0 (no pain), 1 (mild pain), 2 (moderate pain), or 3 (severe pain). At the final assessment (week 12), a participant satisfaction survey was deployed.

#### Objective Study Task: Wrist Range of Movement Test

**Figure 1:**
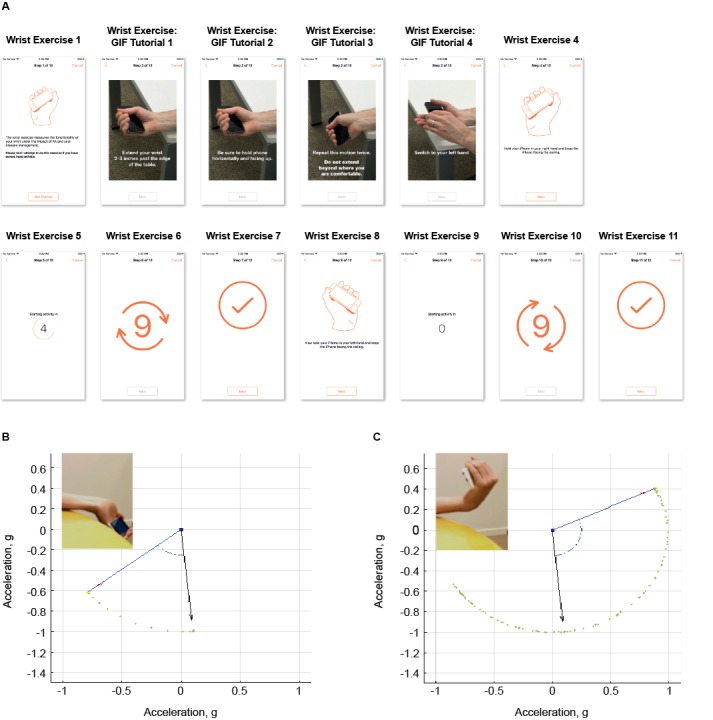
Wrist range of movement (ROM) exercise. (A) Instructions for the wrist ROM exercise provided to participants via the app. (B, C) Wrist ROM preliminary validation based on experimental test data. (B) Relative change in phone orientation (blue arrow) at full wrist extension with respect to initial orientation (black arrow). (C) Relative change in phone orientation at full wrist flexion. In both examples, the video frame from the task performance at the moment of measurement is displayed as a reference for visual inspection. Green dots correspond to phone orientation at previous measurements.

Given the shortage of validated techniques available to assess wrist ROM and the lack of a reference standard comparator, we developed a novel objective wrist ROM exercise for this study. The exercise was to be completed at weeks 1 and 12 (Multimedia Appendix 1). Briefly, participants were instructed to sit down and, in turn, to place their forearm at the edge of a standard-sized table, holding the smartphone in their hand, and to flex and extend their wrist joint to its maximum ROM (Figure 1, part A). We used raw sensor data from the smartphone gyroscope and accelerometers, captured by the app during this exercise, to assess the extent of flexo-extension ROM of each wrist joint as an objective measure of disease activity. We developed mathematical algorithms in Matlab 2016 (The Mathworks Inc) to convert the raw sensor data into ROM data. Figure 1 parts B and C illustrate the process for ROM extraction based on the phone’s orientation computed by the algorithm. Images from experimental data acquisition are also displayed for reference.

### Data Handling and Analysis

Data collected from the PARADE app were stored in secure 21CP11-compliant servers within the Medidata clinical research platform. Data were converted into JavaScript Object Notation files. Completed questionnaire data were subsequently converted into statistical analysis system datasets. All study data were anonymized, and no personally identifying information was obtained as part of the study data. The study was primarily a feasibility study of the Apple ResearchKit app, and the data analyses focused on descriptive information. We included data from participants who discontinued the study when analyzable. No missing data imputation was performed. As this was a pilot study to investigate the feasibility of using a smartphone app to conduct an observational study, we anticipated no specific results.

## Results

### Study Enrollment and Data Collection

We conducted the PARADE real-world observational study in the United States between July and November 2016. Within 1 month of launch, 1170 downloads of the PARADE app were completed, the majority in response to Facebook advertisements (1018/1170, 87.01%). Of these, 428 proceeded to consent; however, 29 individuals consented outside the 1-month window and therefore we did not include their data in the evaluation.

**Figure 2:**
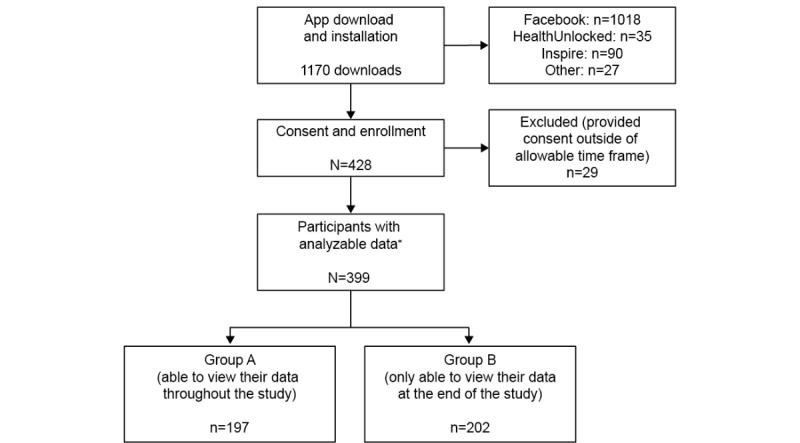
Patient Rheumatoid Arthritis Data From the Real World (PARADE) app study recruitment. *Defined as those completing all demographic questions.

**Table 1:** Demographics and baseline clinical characteristics.

Characteristics		Group A (n=197)	Group B (n=202)	Total (N=399)
Female, n (%)		158 (80.2)	164 (81.2)	322 (80.7)
Age (years), mean (SD)		49.2 (12.48)	46.8 (12.00)	47.9 (12.28)
**Ethnicity, n (%)**				
	White	154 (78.2)	168 (83.2)	322 (80.7)
	African American	5 (2.5)	11 (5.4)	16 (4.0)
	Hispanic	27 (13.7)	13 (6.4)	40 (10.0)
	Asian	7 (3.6)	4 (2.0)	11 (2.8)
	Other	4 (2.0)	6 (3.0)	10 (2.5)
Body mass index (kg/m^2^), mean (SD)		29.3 (7.03)	29.7 (7.55)	29.5 (7.29)
**Education, n (%)**				
	Middle school or below	4 (2.0)	1 (0.5)	5 (1.3)
	High school	44 (22.3)	42 (20.8)	86 (21.6)
	College	91 (46.2)	111 (55.0)	202 (50.6)
	Graduate school	58 (29.4)	48 (23.8)	106 (26.6)
**Smoking history, n (%)**				
	Current	18 (9.1)	19 (9.4)	37 (9.3)
	Previous	61 (31.0)	60 (29.7)	121 (30.3)
	Never	118 (59.9)	123 (60.9)	241 (60.4)
**Duration (years) since diagnosis, n (%)**				
	<2	60 (30.5)	62 (30.7)	122 (30.6)
	2-5	44 (22.3)	47 (23.3)	91 (22.8)
	5-10	45 (22.8)	46 (22.8)	91 (22.8)
	>10	48 (24.4)	47 (23.3)	95 (23.8)

The remaining 399 (34.10%) of the 1170 participants enrolled, consented, and provided complete demographic data, and hence we considered them to have contributed analyzable data (Figure 2).

**Figure 3:**
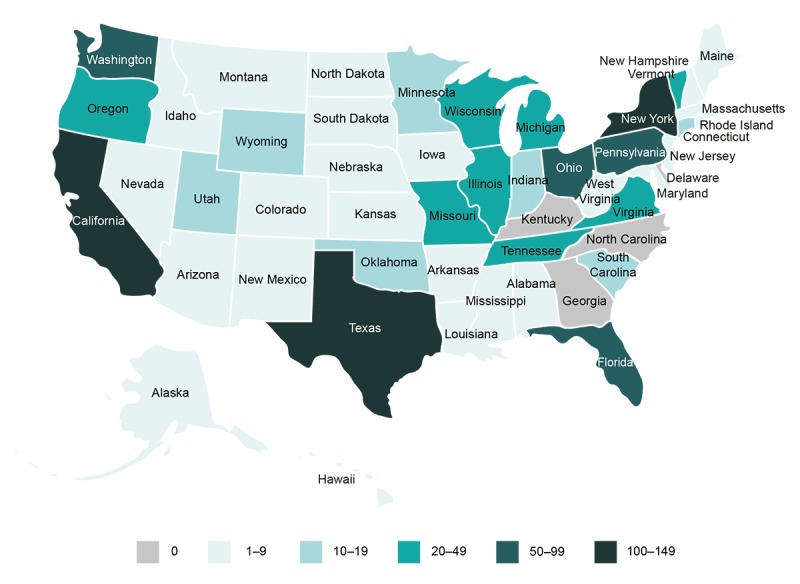
Geographic distribution of participants within the United States.

Table 1 lists participant demographics and clinical characteristics. Table 2 lists the participants’ current medications. The study population was predominately female (322/399, 80.7%) and white (322/399, 80.7%) with a mean age of 47.9 (SD 12.28) years; 77.2% (308/399) of participants were educated to college or graduate school level (Table 1). Disease duration was less than 2 years in 30.6% (122/399) of participants, between 2 and 5 years in 22.8% (91/399), between 5 and 10 years in 22.8% (91/399), and over 10 years in 23.8% (95/399). Differences between group A and group B were minimal, suggesting that the app-programmed randomization worked effectively. Patients were enrolled from a wide geographic distribution across the United States (Figure 3 [25]). Consistent with our geotargeting approach, California, Texas, Pennsylvania, New York, and Florida were among the states with the highest number of downloads.

### Subjective Study Tasks

**Figure 4:**
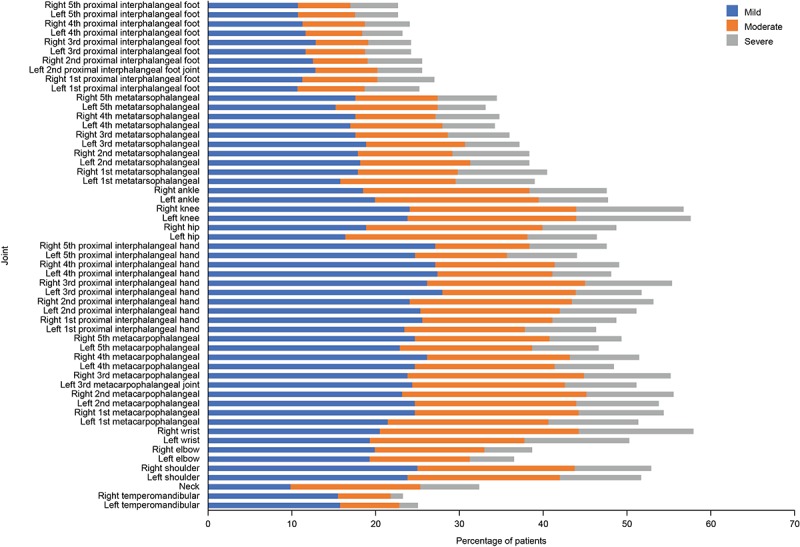
Joint pain map. Percentage of patients reporting any pain in each of 55 joints at week 1 (n=336).

Joint pain was the most frequently reported RA symptom to bother study participants (344/393, 87.5%). Consistent with RA symptoms, painkillers, nonsteroidal anti-inflammatory drugs, corticosteroids, and methotrexate were the most commonly used medications (Table 2). Participant self-reported joint pain from the joint-pain map assessment was completed by 83.7% (334/399) of participants at week 1. Responses included identification of painful joints and the severity of pain in each individual joint scored on a scale of 0 to 3. At week 1, the right wrist (195/336, 58.0%) and left knee (194/336, 57.7%) were the most frequently cited locations of mild, moderate, or severe joint pain (Figure 4). Joint pain was most commonly reported to be mild and was similar for the left and right joints.

Data from PRO measures collected via the app showed no substantial changes in any of the scales throughout the study; however, the number of participants decreased throughout the study (Multimedia Appendix 2).

**Table 2:** Current rheumatoid arthritis medications.

Medication	Study population, n (%)		
	Group A (n=194)	Group B (n=194)	Total (n=388)
Painkillers	89 (45.9)	81 (41.8)	170 (43.8)
NSAIDs^a^	111 (57.2)	83 (42.8)	194 (50.0)
Corticosteroids	57 (29.4)	60 (30.9)	117 (30.2)
Methotrexate	76 (39.2)	79 (40.7)	155 (39.9)
Azathioprine	3 (1.5)	2 (1.0)	5 (1.3)
Auranofin	1 (0.5)	0	1 (0.3)
Chloroquine	1 (0.5)	0	1 (0.3)
Hydroxychloroquine	46 (23.7)	61 (31.4)	107 (27.6)
Leflunomide	13 (6.7)	17 (8.8)	30 (7.7)
Mycophenolate	3 (1.5)	0	3 (0.8)
Sulfasalazine	11 (5.7)	18 (9.3)	29 (7.5)
Abatacept	14 (7.2)	15 (7.7)	29 (7.5)
Adalimumab	23 (11.9)	19 (19.8)	42 (10.8)
Certolizumab	7 (3.6)	3 (1.5)	10 (2.6)
Etanercept	14 (7.2)	20 (10.3)	34 (8.8)
Golimumab	4 (2.1)	7 (3.6)	11 (2.8)
Infliximab	6 (3.1)	10 (5.2)	16 (4.1)
Rituximab	4 (2.1)	7 (3.6)	11 (2.8)
Tocilizumab	7 (3.6)	6 (3.1)	13 (3.4)
Tofacitinib	9 (4.6)	9 (4.6)	18 (4.6)
Others	19 (9.8)	21 (10.8)	40 (10.3)

^a^NSAID: nonsteroidal anti-inflammatory drug.

### Objective Study Task: Wrist Range of Movement Test

**Figure 5:**
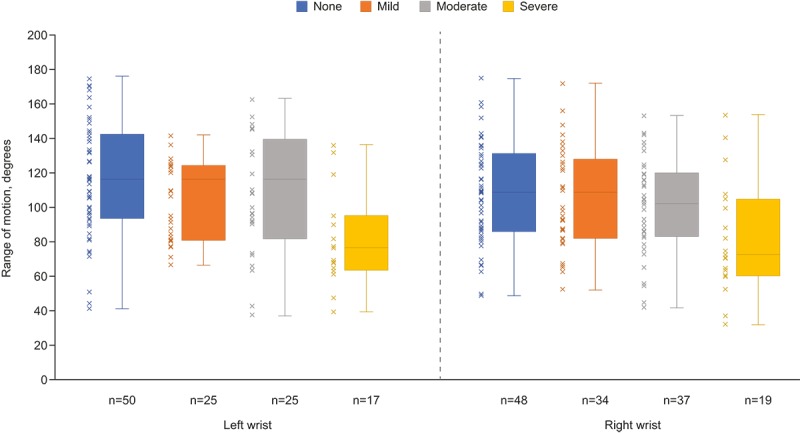
Association between patient wrist range of movement and reported level of wrist pain from the joint pain map assessment at week 1. Boxes represent the upper and lower quartiles; the line inside each box represents the median; the whiskers extending vertically from the boxes represent the range.

We developed a novel tool to objectively evaluate the participants’ wrist joint ROM and tested it for the first time in this study. The novel wrist ROM task was carried out by 71.4% (285/399) of participants at week 1. To evaluate potential associations between this objective test and patient-reported joint pain, we compared the wrist ROM data with the wrist joint pain scores from the joint-pain map assessment of each participant. Severe patient-reported wrist pain appeared to be inversely linked with the wrist ROM measured by the app (Figure 5).

### App Evaluation and Participant Retention

At the beginning of the study, we asked participants whether they would prefer to participate in a study conducted at a clinic or using a mobile app; 73.2% (292/399) expressed a preference for a mobile app, 3.0% (12/399) preferred a clinic-based study, and 13.8% (55/399) answered both, with the remainder having no preference.

**Figure 6:**
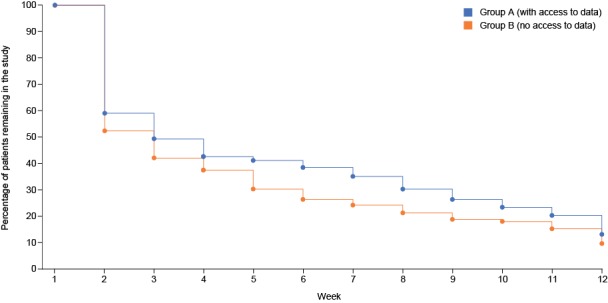
Proportion of patients retained within the study over 12 weeks.

At week 2, 162 of 399 (40.6%) participants completed at least one study assessment; this decreased to 45 of 399 (11.3%) participants at week 12. We did not collect reasons for attrition. The percentage of participants remaining in the study was slightly greater among those who had daily access to their data than among those who did not (26/197, 13.2% vs 19/202, 9.4%, respectively; Figure 6); however, the number of participants remaining in both groups was low. We did not statistically compare retention rates due to the high rate of attrition.

## Discussion

### Principal Findings

The PARADE study was, to our knowledge, the first industry-sponsored study in which patients with RA could self-recruit, consent, enroll, and report data entirely via their iPhone using a ResearchKit app. This study demonstrated the feasibility of using smartphones to conduct a real-world study. In particular, the enrollment approach was successful in obtaining participants from a wide geographic distribution across the United States, as well as a wide ethnic diversity and a demography representative of patients with RA. Within 30 days, we exceeded our enrollment target of 300, demonstrating that the use of digital platforms to reach a large RA population can result in rapid study enrollment. The use of a smartphone was well received by the participants, with 73.2% (292/399) reporting a preference for participating in a mobile app–based study over a site-based study; however, this finding may not be representative of the general RA population.

To reduce any inherent enrollment bias and to ensure the validity of future studies using smartphones for data collection, it is particularly important to ensure that a representative sample of patients can access and enroll in the study. The PARADE study population was comparable with an RA population from a study that used more traditional methods of data collection, the Consortium of Rheumatology Researchers of North America (CORRONA) RA registry, believed to be representative of the US RA population [25]. Similarly, high proportions of women participated, consistent with the higher prevalence of RA in women; however, we found that the PARADE study population were younger and were more educated than those in the CORRONA registry (Table 3). Presumably this reflects the increasing likelihood of a younger and more highly educated demographic owning a smartphone [26]. Participants in the PARADE study had a greater ethnic diversity (19% other ethnicities) compared with those represented in the CORRONA registry (11%), suggesting that using downloadable smartphone apps to engage patients may be effective in recruiting a broadly representative ethnic population but may favor participation by individuals with higher education and younger age.

The app enabled collection of a range of RA-related data, including medications, symptoms, quality of life, joint-pain map data, and wrist ROM measures. The app has the potential for data collection at a higher frequency (eg, multiple points per day) than would be logistically possible in standard clinical studies; therefore, it can provide a more holistic view of disease exacerbation and remission. Furthermore, a previous study demonstrated that capture of Disease Activity Score in 28 joints data using a dedicated smartphone app correlated well with monthly clinical assessments of RA disease activity [15].

**Table 3:** Demographic profile of Patient Rheumatoid Arthritis Data From the Real World (PARADE) participants compared with representative data from the Consortium of Rheumatology Researchers of North America (CORRONA) registry of US patients with rheumatoid arthritis [25].

Demographic	PARADE	CORRONA
Age (years), mean (SD)	47.9 (12.3)	58.9 (13.4)
Female, n (%)	322 (81)	19,242 (77)
White, n (%)	322 (81)	22,240 (89)
Other ethnicities, n (%)	77 (19)	2749 (11)
College/graduate school educated, n (%)	308 (77)	13,744 (55)

Whereas other studies have reported issues with storage and transmission of data files from the phone, due to file size [14], we encountered no such challenges or difficulties in this study.

The design of studies in which patients self-report via electronic interfaces has the potential to revolutionize how clinical research is conducted [27] but is limited by a relative lack of simple and validated objective measures that can be captured electronically. This has led to a predominance of subjective assessments, typically patient questionnaires or PRO measures. Some studies have observed that such self-reporting can be prone to bias, with patients over- or underestimating the true situation [12]. Development of methods that can provide objective as well as subjective data are needed to improve the breadth and quality of results. However, some previous attempts to obtain objective measures remotely (eg, by connecting a hand dynamometer to a smartphone) have required additional instrumentation [28]. We explored the use of a smartphone app to capture objective data directly relevant to disease activity via the smartphone-embedded sensors to record ROM in the wrist. Validated tools to evaluate wrist ROM are lacking, and there is no reference standard comparator available. Previous work, however, has demonstrated the reliability of smartphone apps for the goniometric evaluation of joint ROM [29,30]. In our study, we saw a link between participants’ subjective assessment of severe wrist pain and functional assessment of wrist ROM, suggesting that combining the use of questionnaires and sensor-based recordings from the smartphone may provide a valuable combination to monitor and quantify patient symptoms and disease impacts. Further research is required to validate the extent to which wrist ROM correlates with RA management and remission.

One common trend observed with the use of smartphones in clinical research is that, while engagement may be initially high, the rate of attrition is also high [31,32]. Although only 41% of our participants provided data at week 2, overall, retention was slightly better among participants who could view their data throughout the study than among those who could view it only at the final assessment (88/197, 44.7% vs 74/202, 36.6%, respectively), although we did not statistically evaluate these results and did not monitor whether participants actually accessed their data. This is in line with the findings of other studies (eg, [14]) where the ability to access personalized data could act as an incentive for patients to continue engagement. For example, in a study using computer-based technology to support a weight-loss program, patients who frequently used smartphone technology to view their progress lost more weight than those who did not [31]. For personalized data to provide an incentive to boost participation, the data provided must be meaningful and valuable to the patient, for example, by tracking improvement or progress toward goals, or flagging potential issues. Previous studies have explored various forms of incentivization to boost engagement, most notably the ability to visualize a medical benefit and payment [32,33]. Other ideas include data sharing, participation in an online participant forum, and a strong initial understanding and belief in the objective of the study [14]. It is likely that a combination of different incentives, tailored to suit each specific population, may be required to obtain maximal engagement.

### Limitations

Our study had several limitations. The proportion of participants completing the study was low, and few people completed the evaluation of the app. Given that there was no medical intervention or other variables introduced that might drive a change in PRO measures over time, we expected little change in data from the PROs over the course of the study. However, the high level of attrition precludes the possibility of longitudinal assessments. Differences in retention rates between groups should be considered with caution. The results from this study are descriptive only, and we did not collect data on whether those patients who were able to access their personal data actually did so. A separate study would be required to further investigate retention rates. The app relied on participants’ self-motivation and accurate self-reporting with no way to authenticate the data. It is important to ensure that, in the design of future studies, data shared with patients must add real value, including clinical value, so that any smartphone data that patients share with their physicians will facilitate clinical decisions, not just exacerbate clinician information overload [33].

### Conclusion

This study successfully demonstrated the feasibility of using a smartphone coupled with ResearchKit to obtain patient-reported data in RA from a real-world perspective. It reports the first use of the smartphone gyroscope to measure wrist joint ROM, which was linked with patient-reported joint pain. We created a bespoke algorithm to derive clinically meaningful information on wrist ROM from raw sensor data. Further details on the methodology and accuracy assessment may be presented in a separate publication. This may lead to development and validation of other novel objective end points using smartphone-integrated sensors and may lead to an expansion of the objective data that can be captured from electronic patient-reported clinical research.

## Appendix

Multimedia Appendix 1 Screenshots from the PARADE app.

Multimedia Appendix 2 Mean patient-reported outcome responses.

## Abbreviations

21CP11: Title 21 of the Code of Federal Regulations Part 11
CORRONA: Consortium of Rheumatology Researchers of North America
GSK: GlaxoSmithKline
PARADE: Patient Rheumatoid Arthritis Data From the Real World
PRO: patient-reported outcome
RA: rheumatoid arthritis
ROM: range of movement
