# Supplementary material for: Using a ResearchKit Smartphone App to Collect Rheumatoid Arthritis Symptoms From Real-World Participants: Feasibility Study
Source: JMIR Mhealth Uhealth. 2018 Sep 13;6(9):e177. doi: 10.2196/mhealth.9656 (PMC6231853; doi:10.2196/mhealth.9656)
Supplement: Multimedia Appendix 1 [file mhealth_v6i9e177_app1.pdf]

POSSIBLE mobile

06-14-16

PARADE app - GSK
IRB Submission Deck

Presentation Created For GlaxoSmithKline

Created By POSSIBLE Mobile

CONTENTS

3 Welcome Screens
4 Welcome Screens, 2
5 Eligibility Screens
6 Consent Screens
7 Consent Screens, 2
8 Consent Screens, 3
9 Passcode Screens
10 Registration Screens
11 Medical History Survey Screens
12 Medical History Survey Screens, 2
13 Medical History Survey Screens, 3
14 Medical History Survey Screens, 4
15 Medical History Survey Screens, 5
16 Task List & Badges Screens
17 Dashboard Screens
18 Dashboard Screens, 2
19 Learn Screens
20 Learn Screens, 2
21 Learn Screens, 3
22 Profile & More Screens
23 Pain Scale Screens
24 Morning Stiffness Survey Screens
25 Mood Scale Screens
26 RA Severity Survey Screens
27 Walk Test Screens
28 Walk Test Screens, 2
29 Walk Test Screens, 3
30 Health Status Survey Screens
31 Health Status Survey Screens, 2
32 HAQ Screens
33 HAQ Screens, 2
34 HAQ Screens, 3
35 HAQ Screens, 4
36 HAQ Screens, 5
37 HAQ Screens, 6
38 HAQ Screens, 7
39 Fatigue Scale Screens
40 Fatigue Scale Screens,2
41 Fatigue Scale Screens, 3
42 Fatigue Scale Screens, 4
43 Joint Pain Map Screens
44 Wrist Exercise Screens
45 Wrist Exercise Screens, 2
46 Wrist Exercise Screens, 3
47 Wrist Exercise Screens, 4
48 End of Study Screens
49 End of Study Screens, 2
50 End of Study Screens, 3

Welcome Screens

POSSIBLE mobile

Welcome Screen: First Card

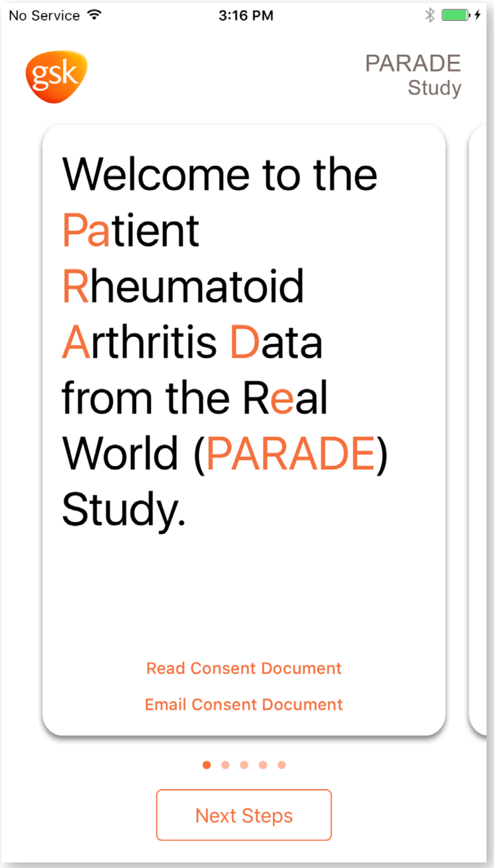

Welcome Screen: Second Card

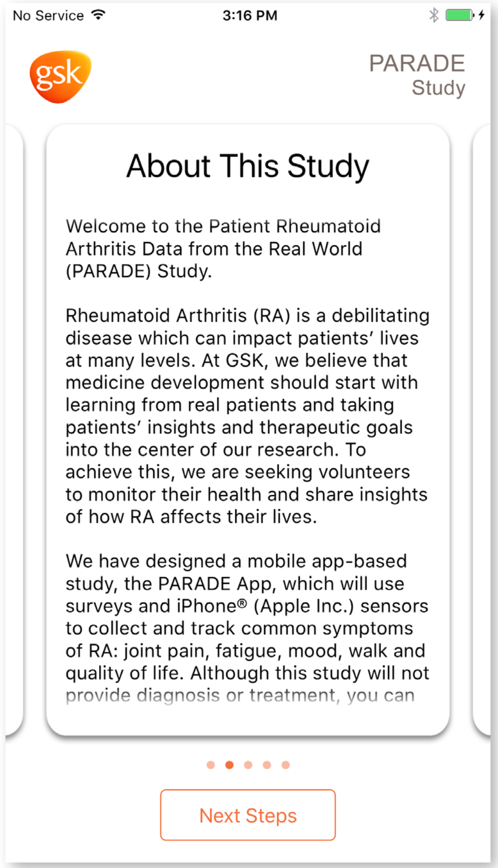

Welcome Screen: Second Card (cont.)

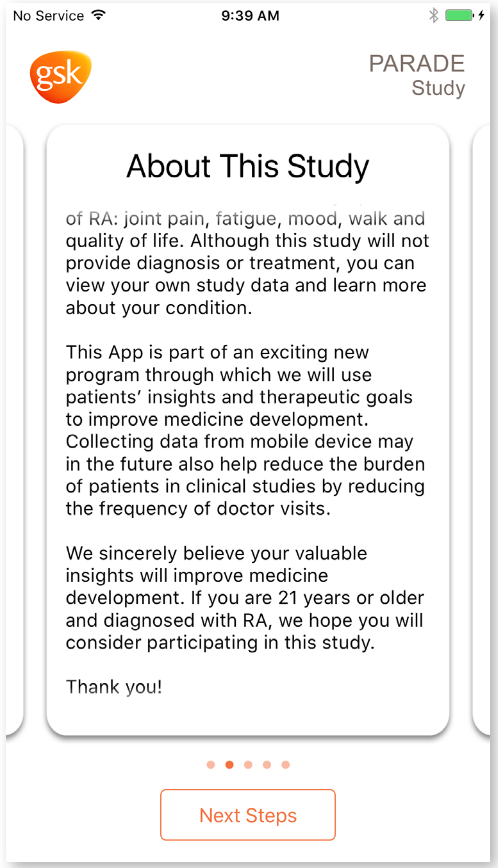

Welcome Screen: Third Card

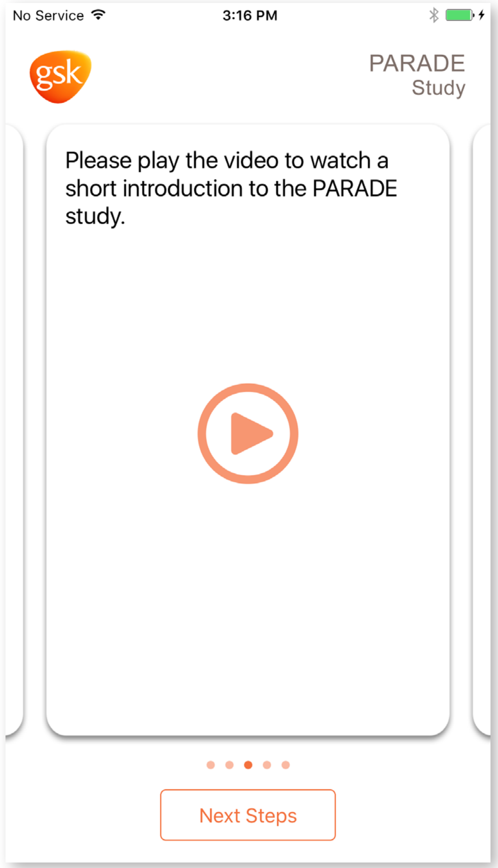

Welcome Screens, 2

POSSIBLE mobile

Welcome Screen: Video

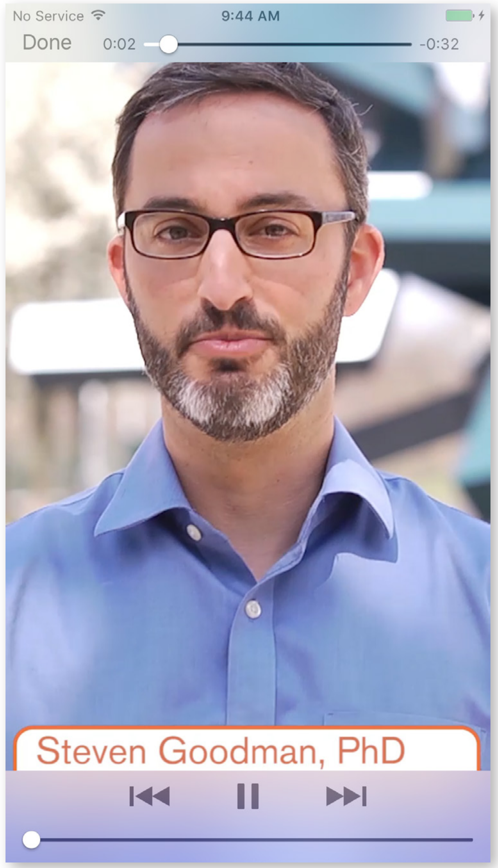

Welcome Screen: Fourth Card

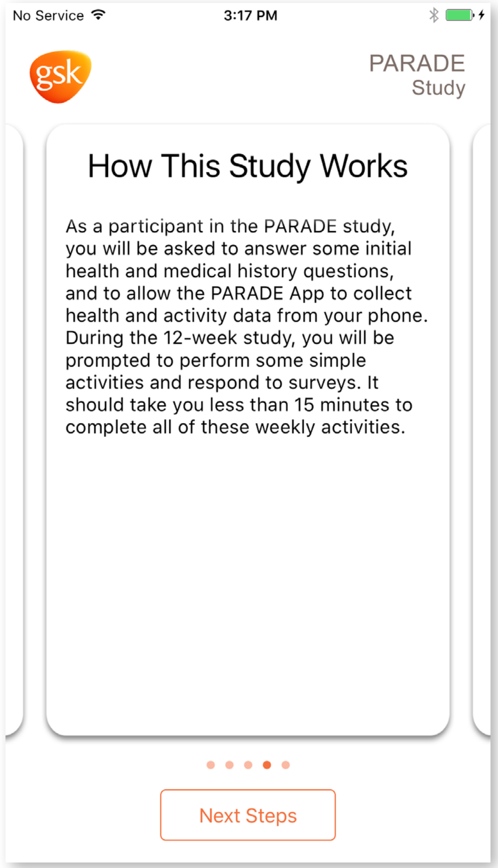

Welcome Screen: Fifth Card

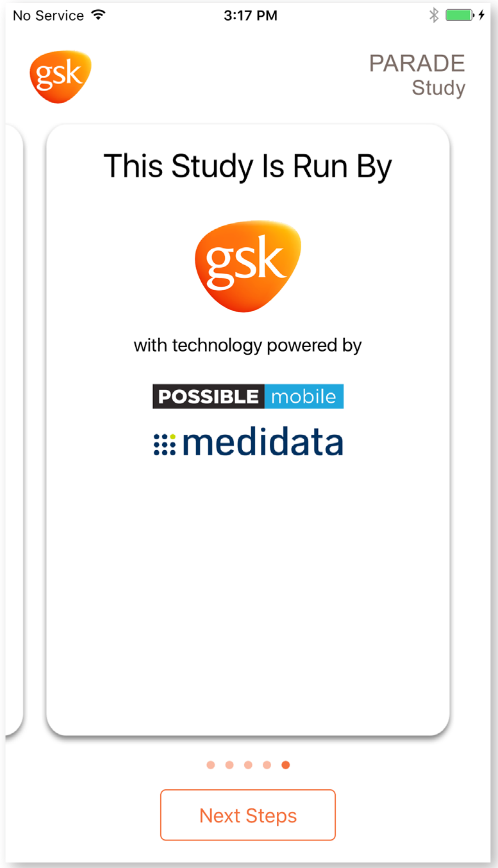

Eligibility Screens

POSSIBLE mobile

Eligibility Screen 1

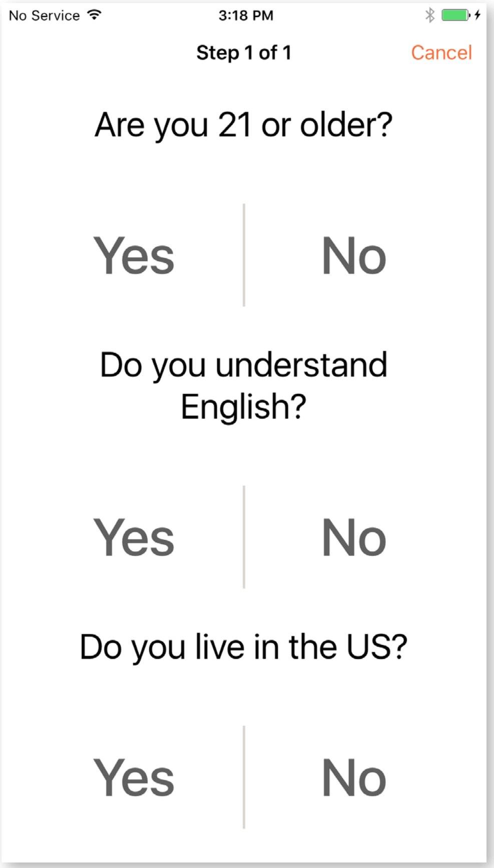

Eligibility Screen 1 (cont.)

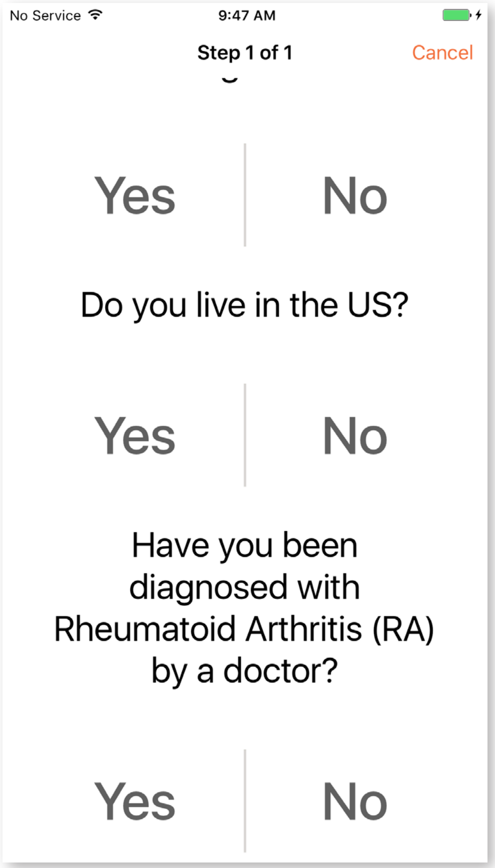

Eligibility Screen 2

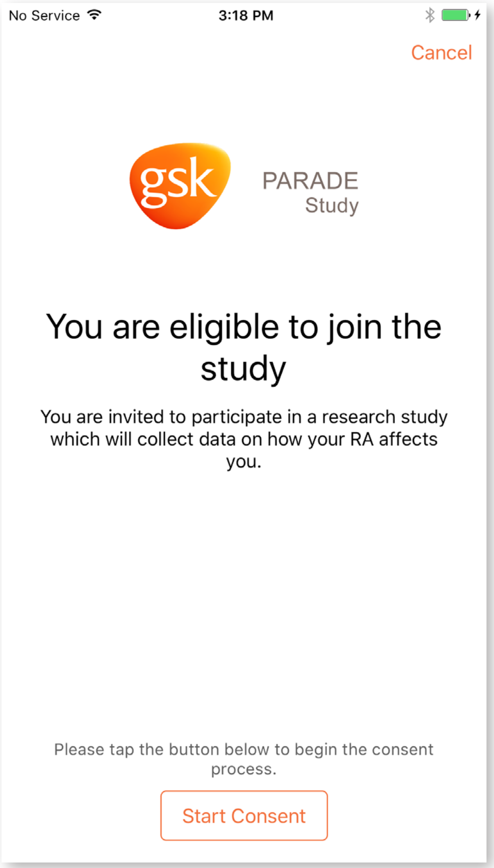

Consent Screens

POSSIBLE mobile

Consent PDF Page 1

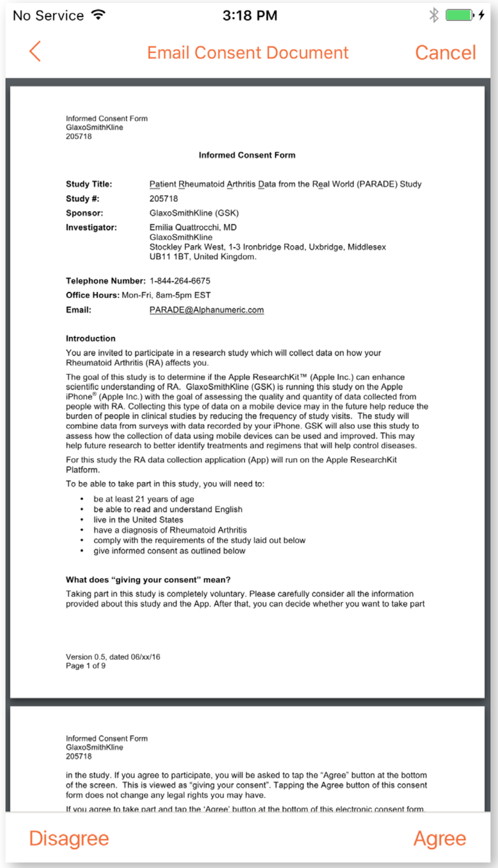

Consent PDF Page 2

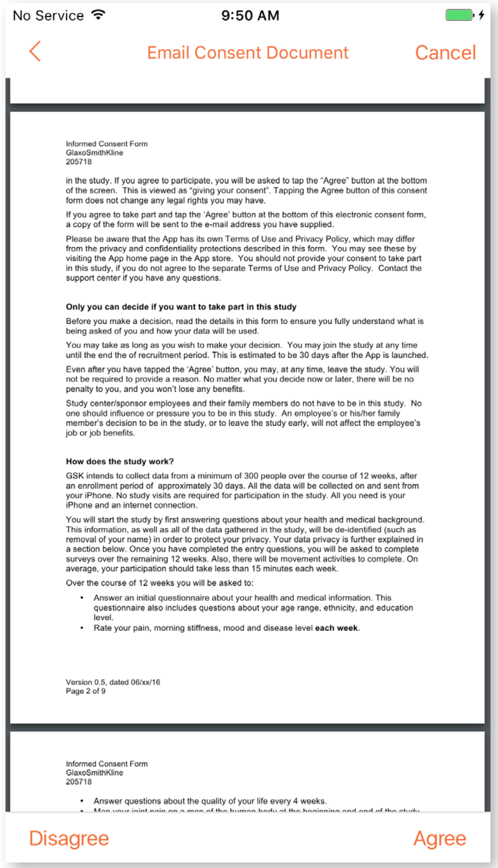

Consent PDF Page 3

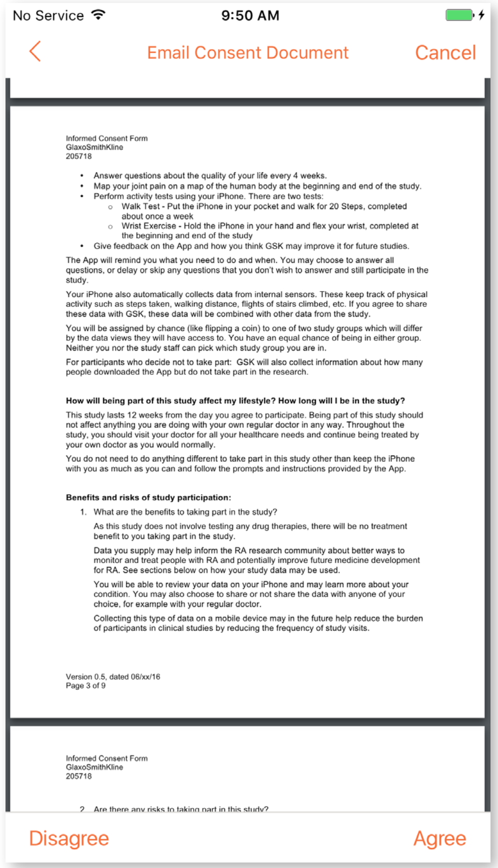

Consent PDF Page 4

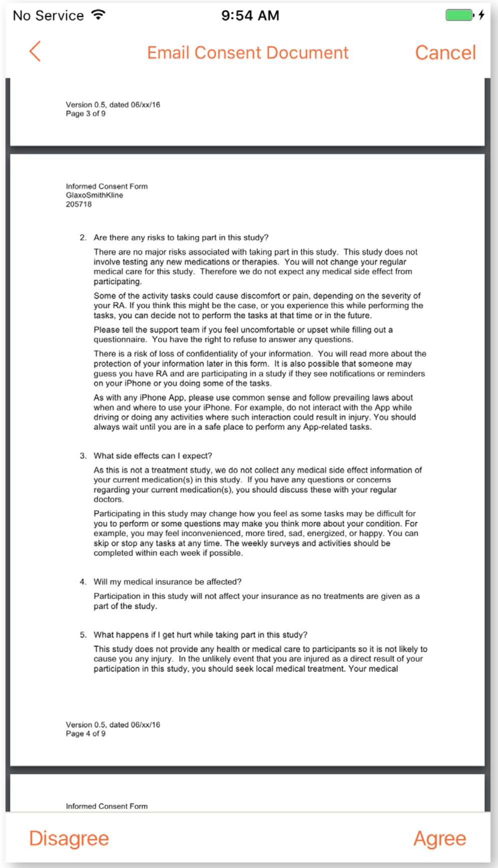

Consent Screens, 2

POSSIBLE mobile

Consent PDF Page 5

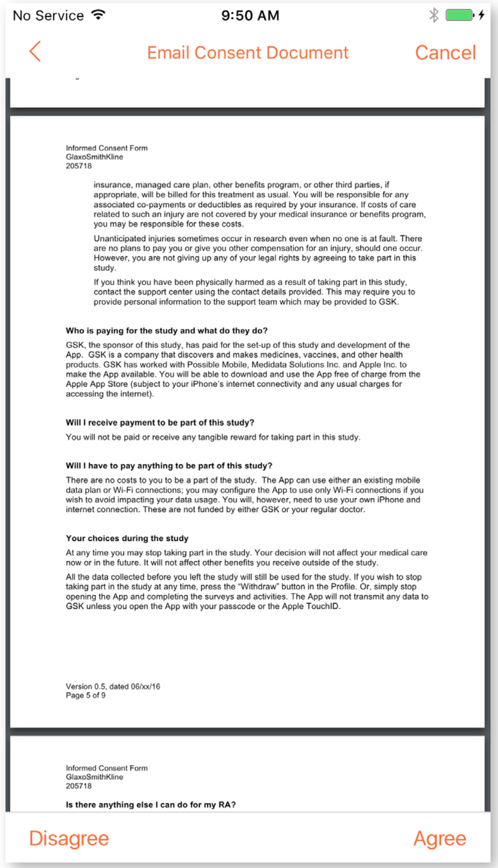

Consent PDF Page 6

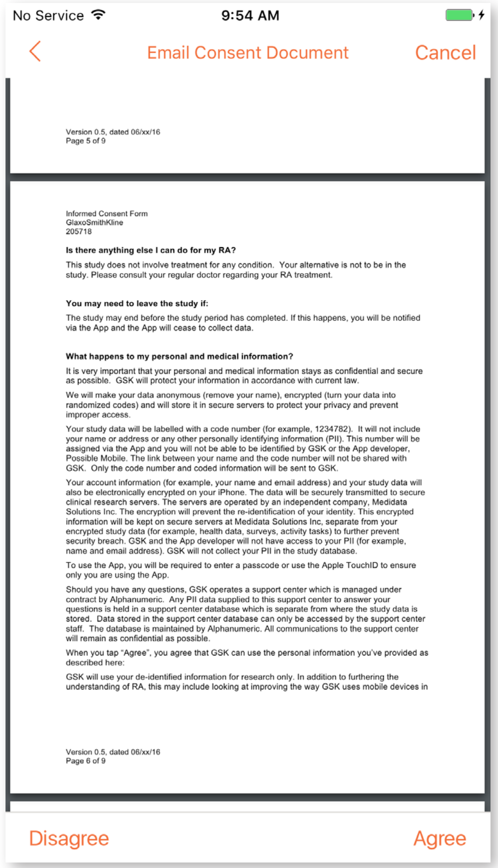

Consent PDF Page 7

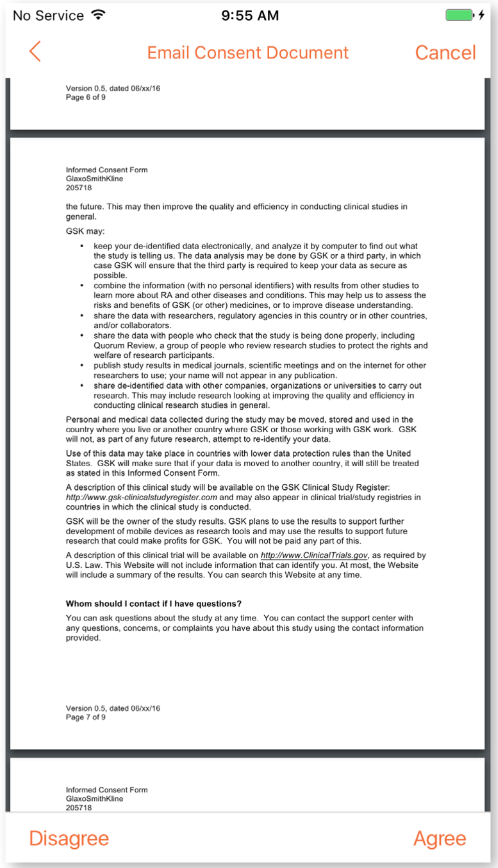

Consent PDF Page 8

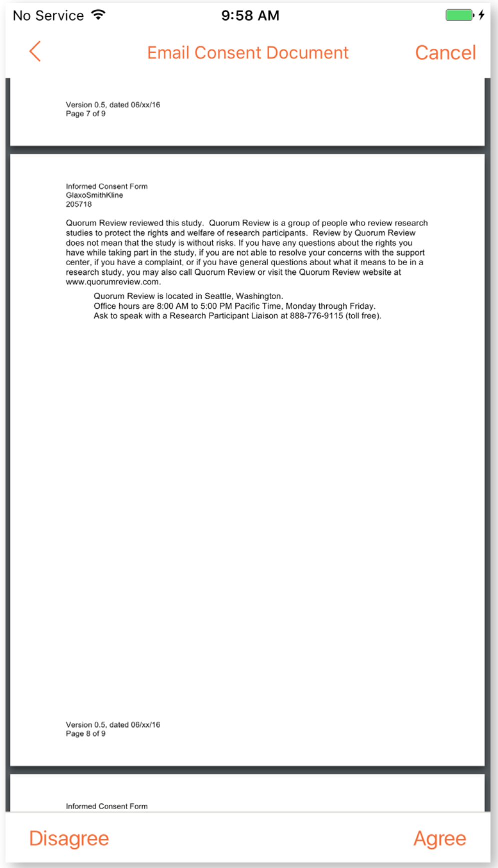

Consent Screens, 3

POSSIBLE mobile

Consent PDF Page 9

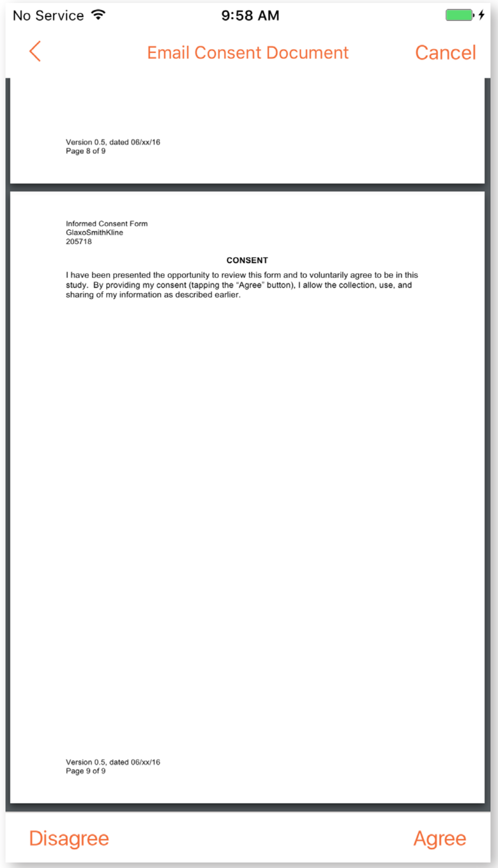

Consent Agree Notification

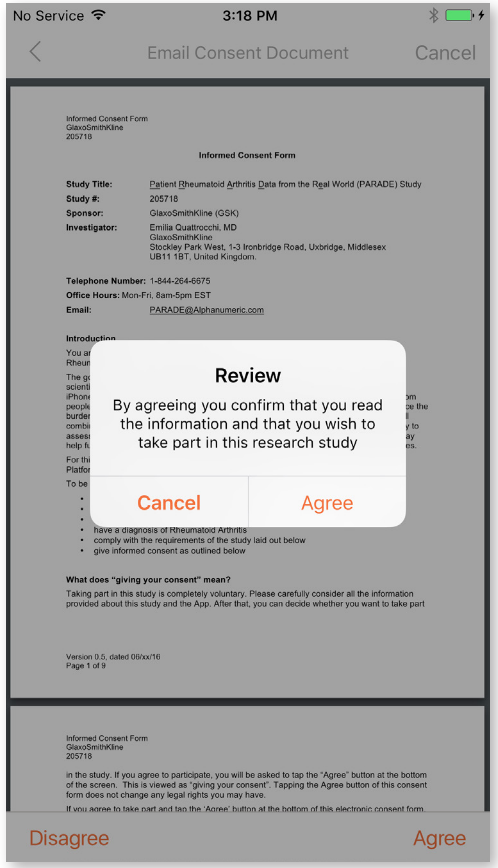

Consent Signature

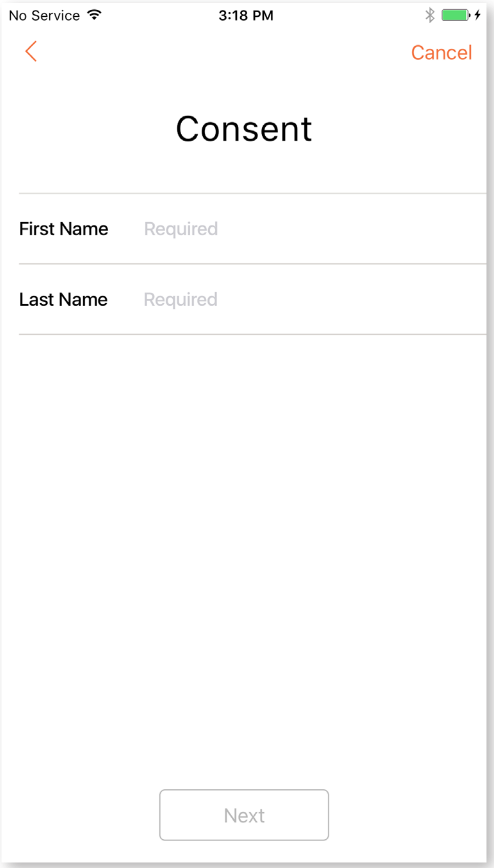

What to Expect

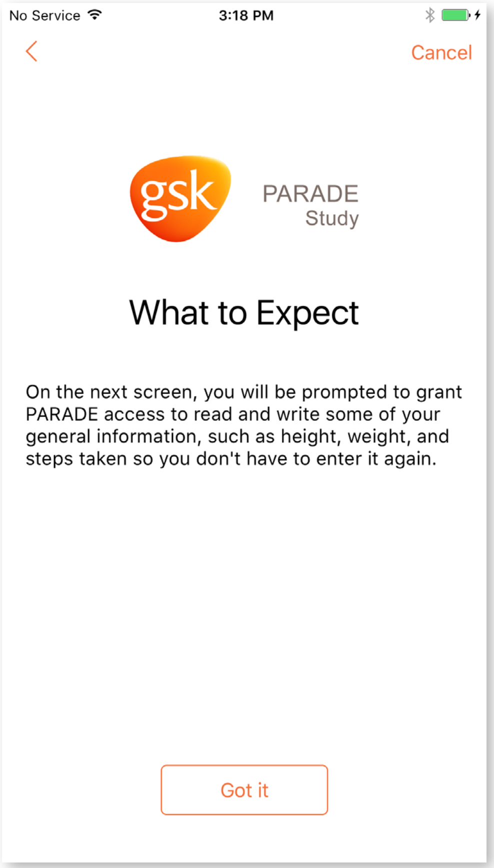

Passcode Screens

POSSIBLE mobile

Enter Passcode

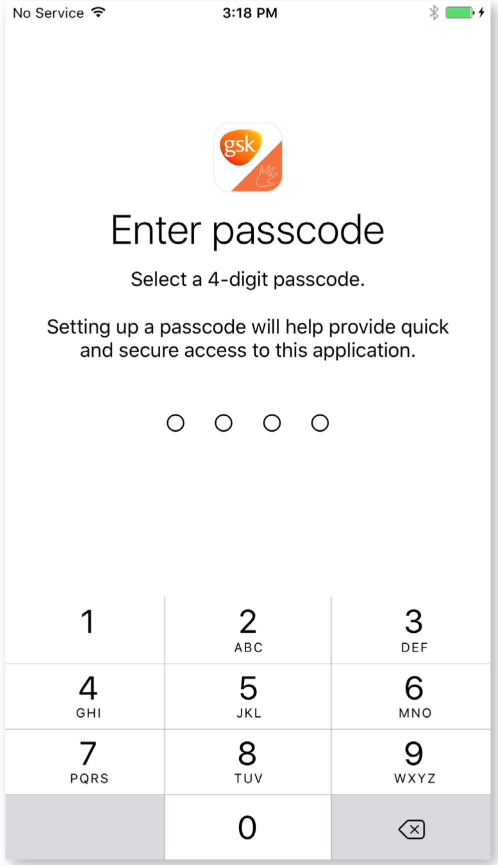

Confirm Passcode

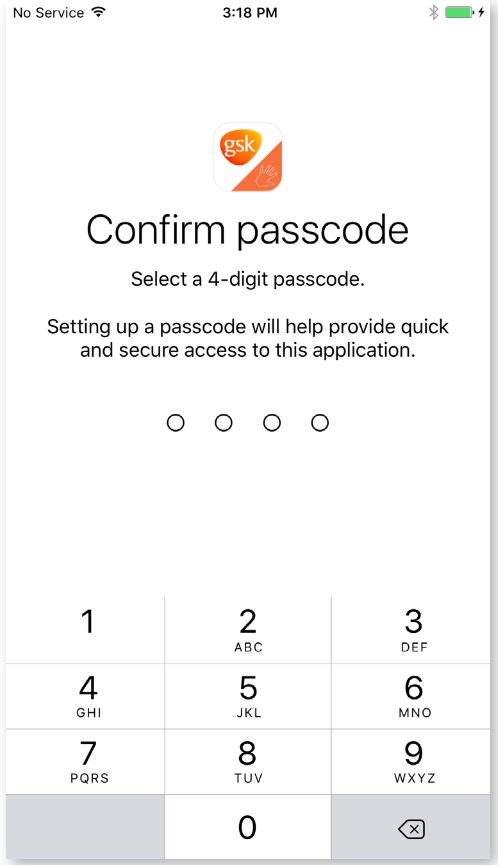

Passcode Saved / Touch ID prompt

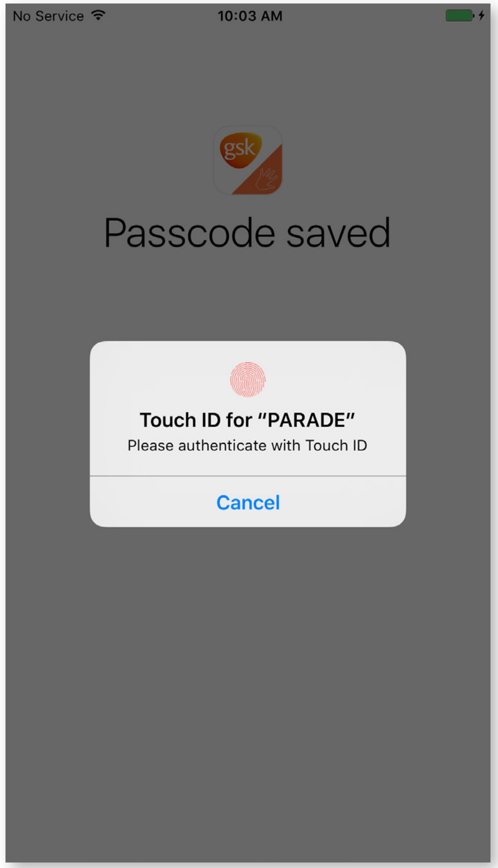

Registration Screens

POSSIBLE mobile

Create Profile

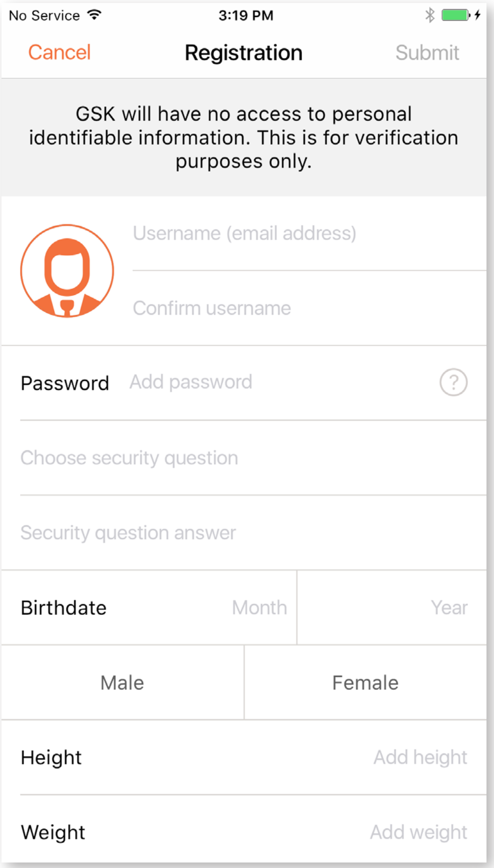

Intro Survey

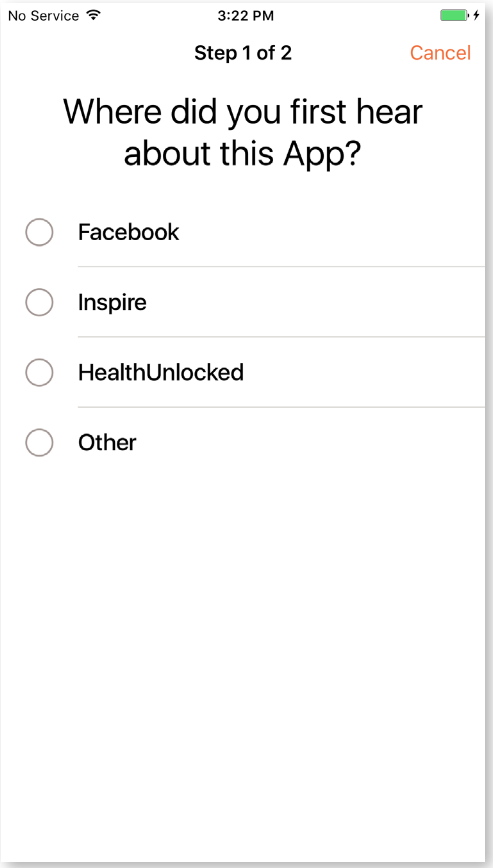

Intro Survey Complete

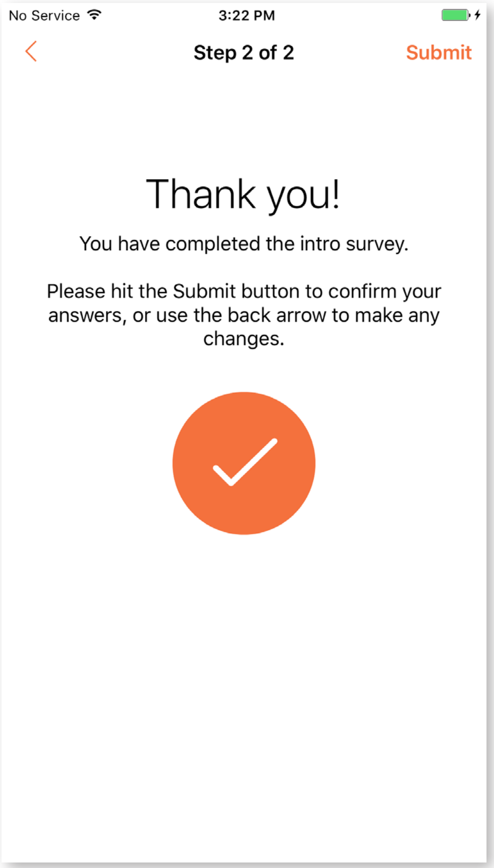

Health and Medical Screens

POSSIBLE mobile

Health and Medical History 1

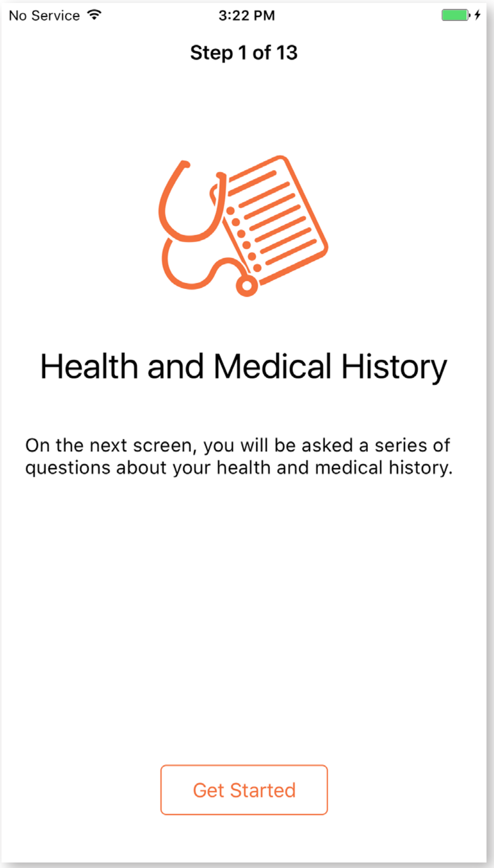

Health and Medical History 2

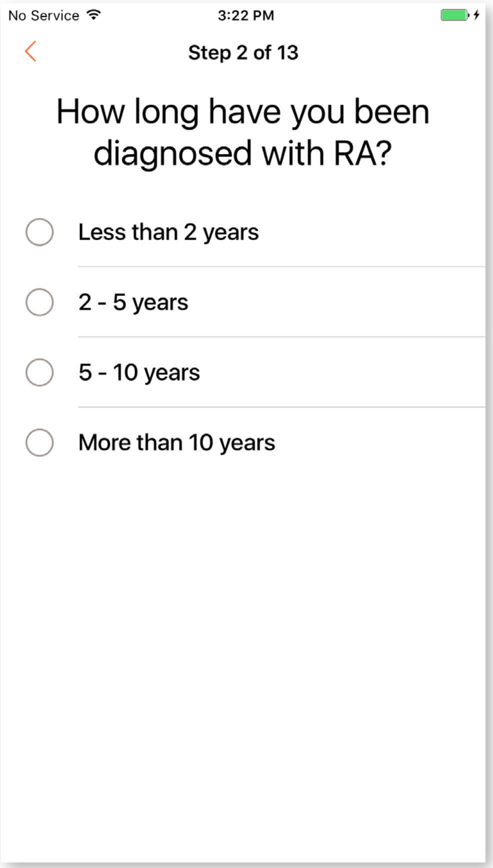

Health and Medical History 3

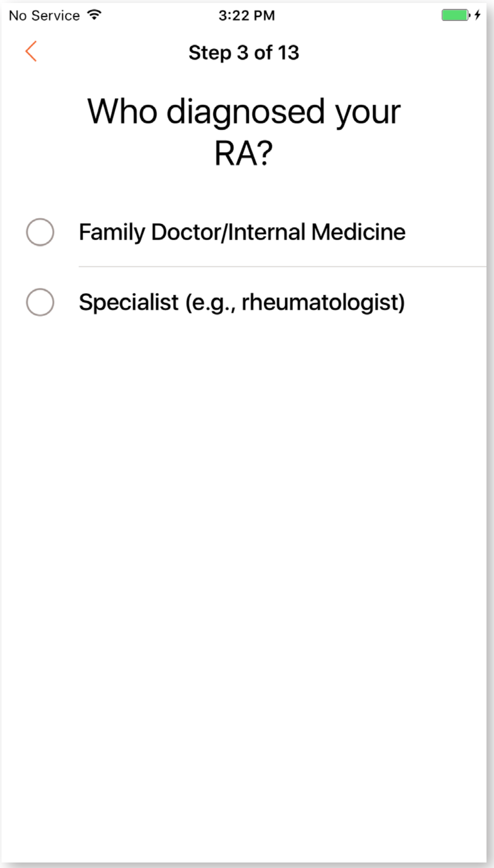

Health and Medical History 4

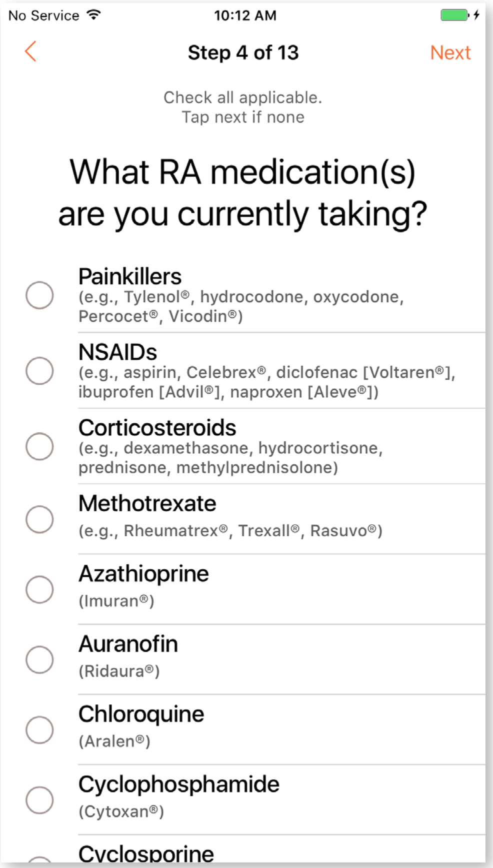

Health and Medical Screens, 2

POSSIBLE mobile

Health and Medical History 4 (cont.)

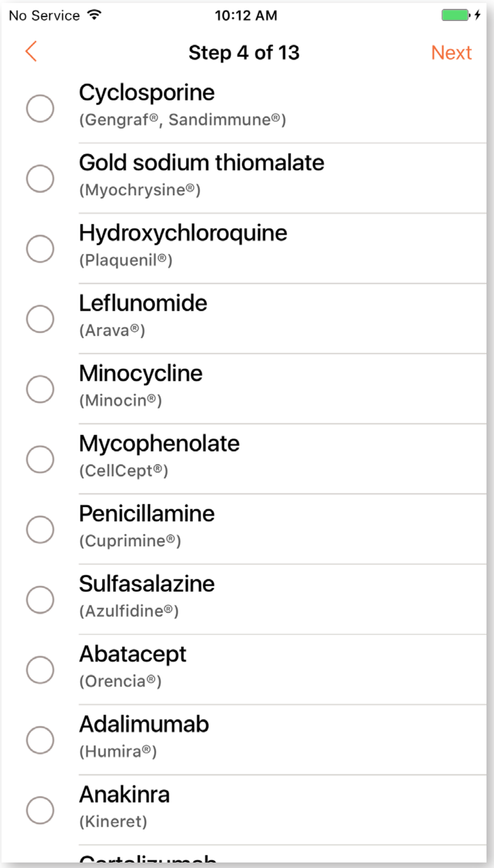

Health and Medical History 4 (cont.)

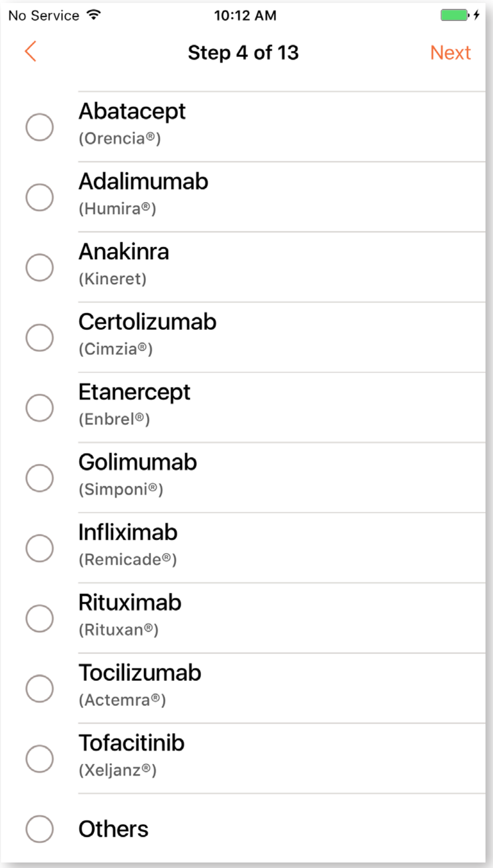

Health and Medical History 5

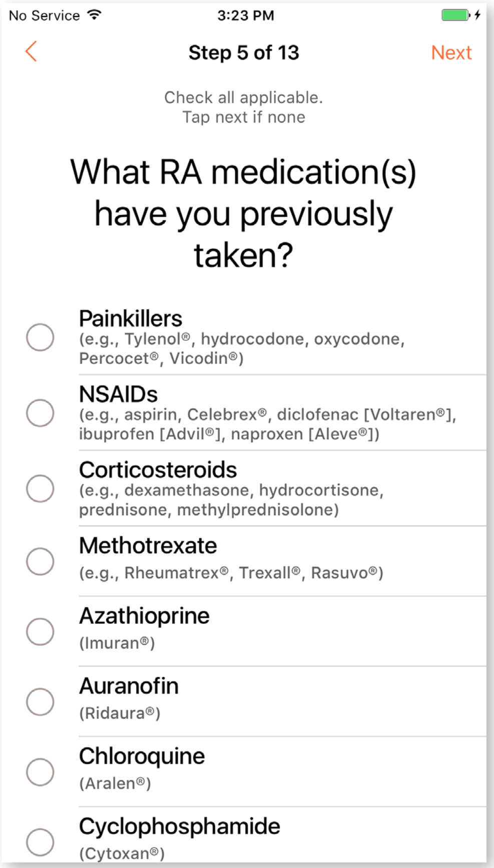

Health and Medical History 5 (cont.)

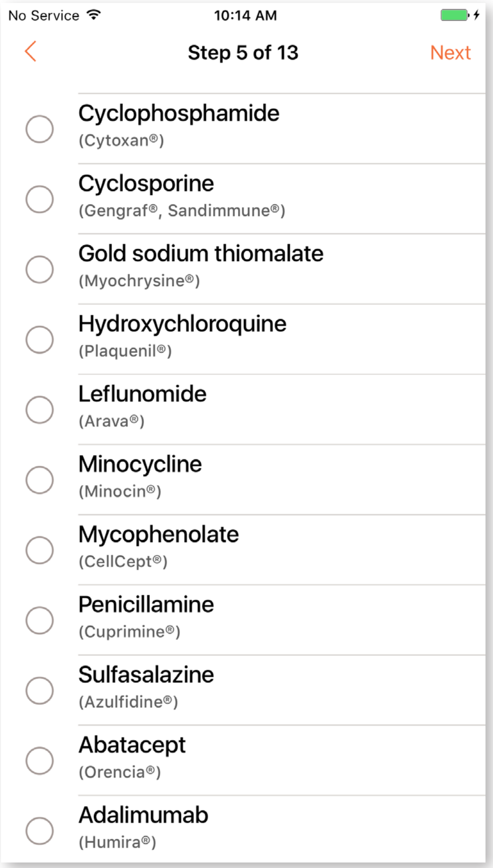

Health and Medical Screens, 3

POSSIBLE mobile

Health and Medical History 5 (cont.)

No Service 10:14 AM

Step 5 of 13 Next

- Abatacept (Orencia®)
- Adalimumab (Humira®)
- Anakinra (Kineret)
- Certolizumab (Cimzia®)
- Etanercept (Enbrel®)
- Golimumab (Simponi®)
- Infliximab (Remicade®)
- Rituximab (Rituxan®)
- Tocilizumab (Actemra®)
- Tofacitinib (Xeljanz®)
- Others

Health and Medical History 6

No Service 3:23 PM

Step 6 of 13

What is your ethnicity?

- Caucasian
- African-American
- Hispanic
- Asian
- Other

Health and Medical History 7

No Service 3:23 PM

Step 7 of 13

Smoking History:

- Current smoker
- Previous smoker
- Never smoked

Health and Medical History 8

No Service 3:23 PM

Step 8 of 13

What is the highest level of education you have achieved?

- Middle school or below
- High school
- College
- Graduate school

Health and Medical Screens, 4

POSSIBLE mobile

Health and Medical History 9

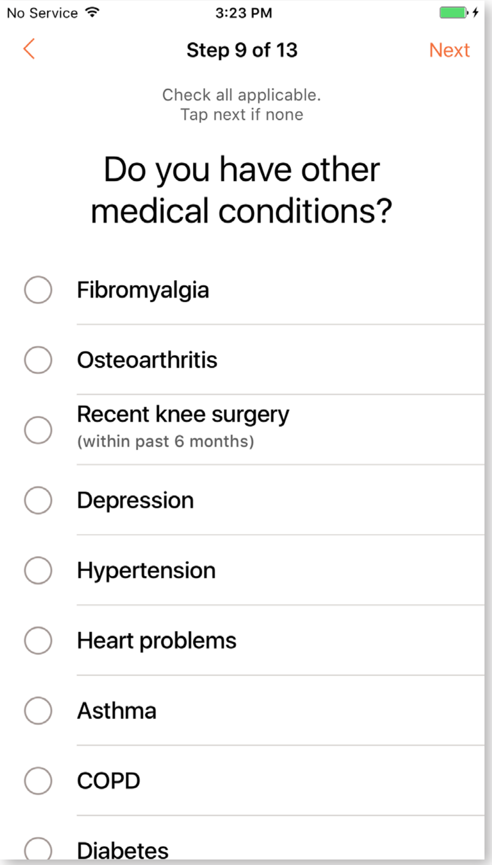

Health and Medical History 9 (cont.)

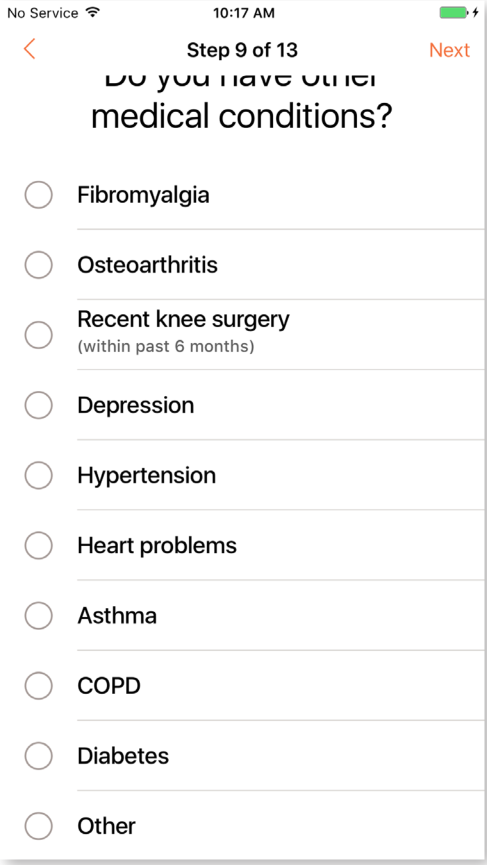

Health and Medical History 10

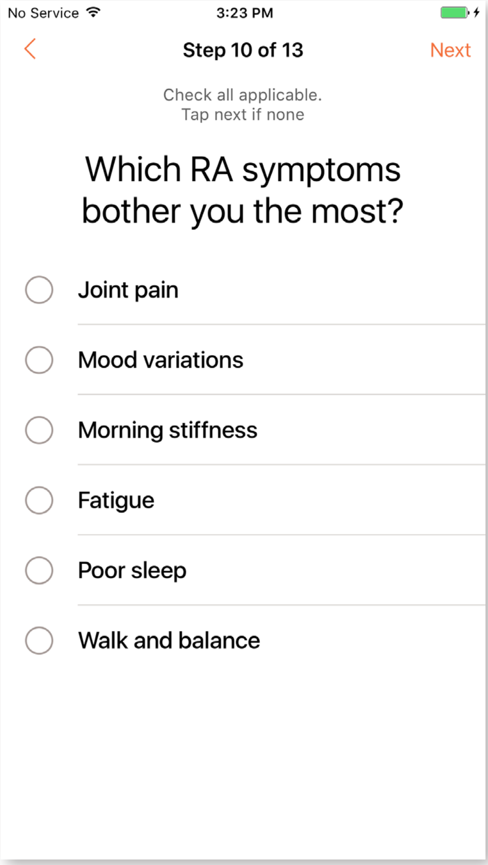

Health and Medical History 11

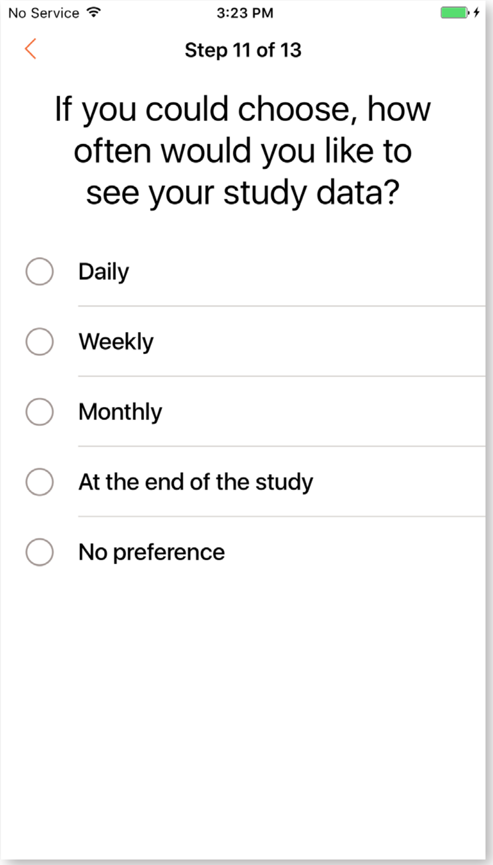

Health and Medical Screens, 5

POSSIBLE mobile

Health and Medical History 12

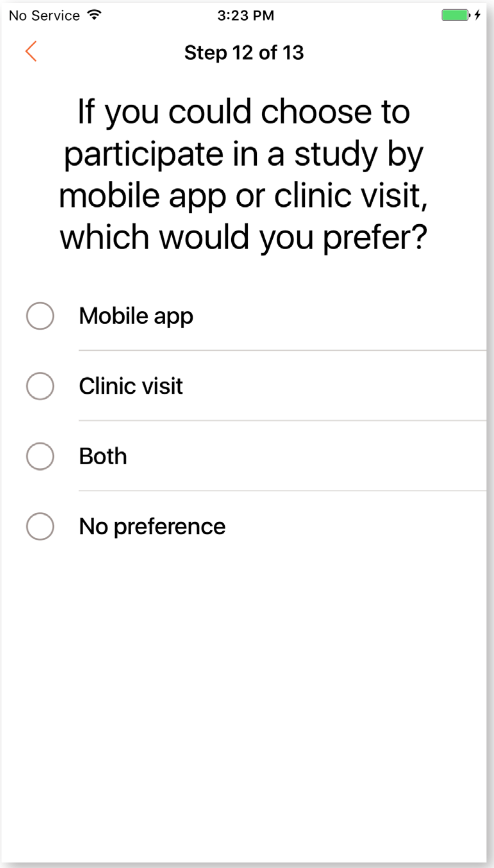

Health and Medical History 13 (cont.)

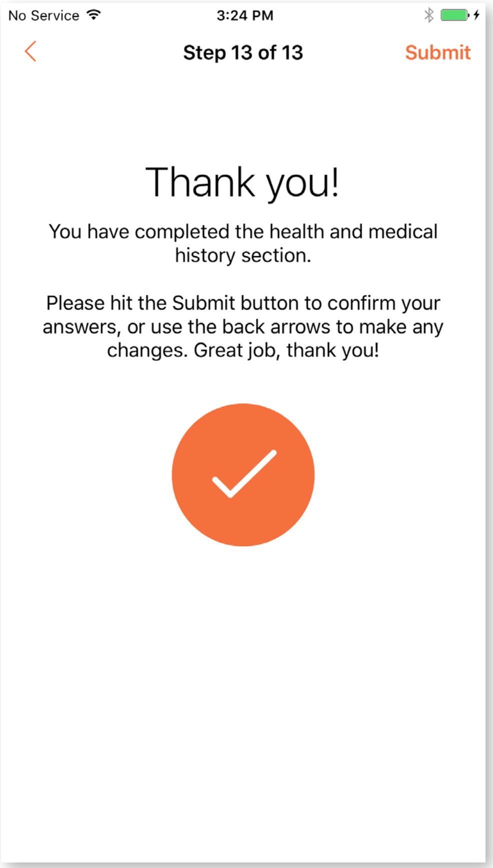

Task List & Badges Screens

POSSIBLE mobile

Task List

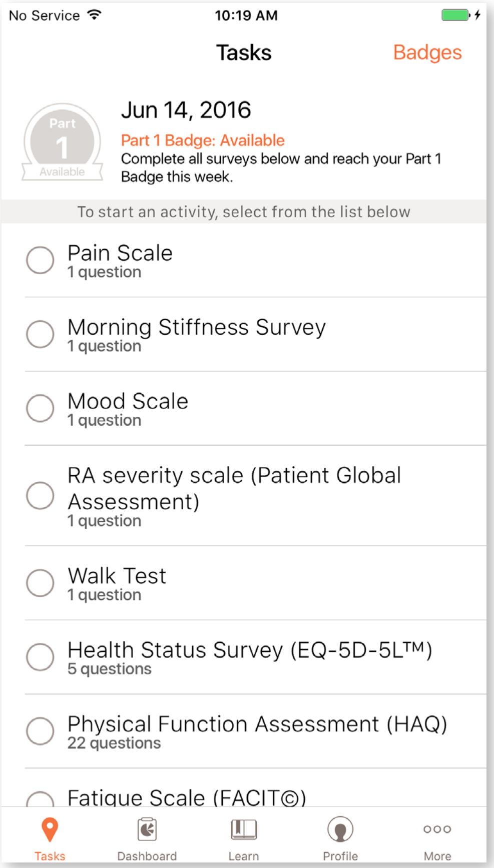

Task List (cont.)

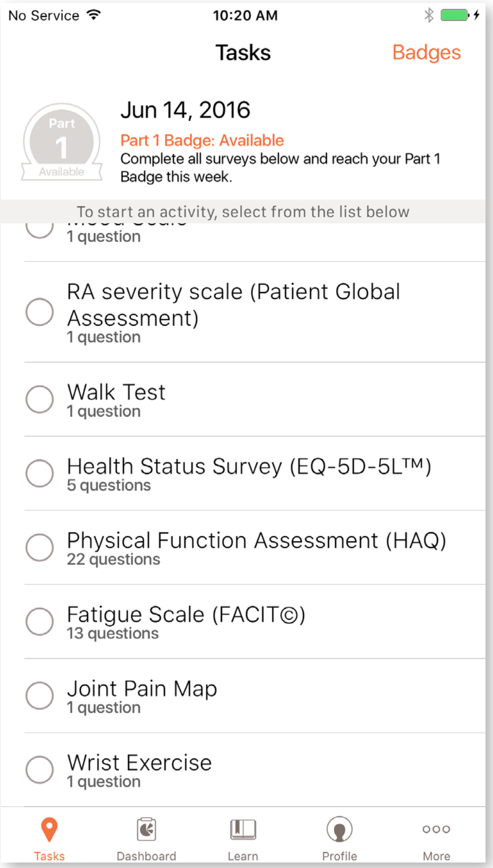

Badges

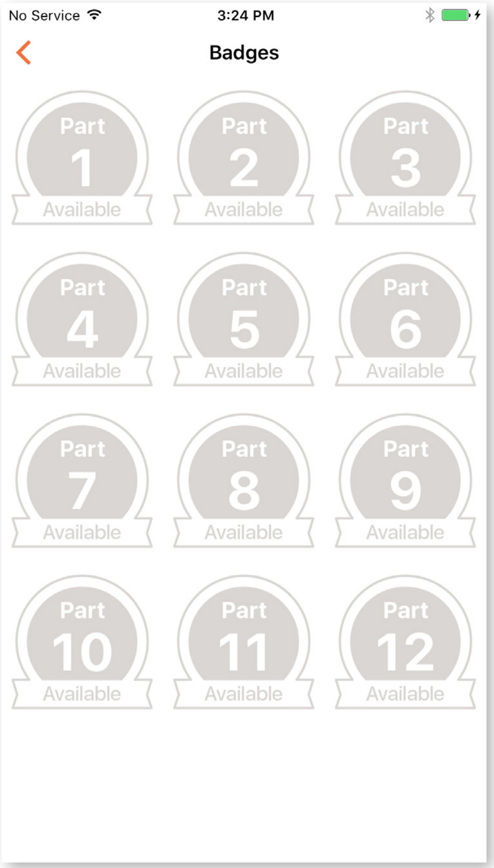

Dashboard Screens

POSSIBLE mobile

Dashboard Group B

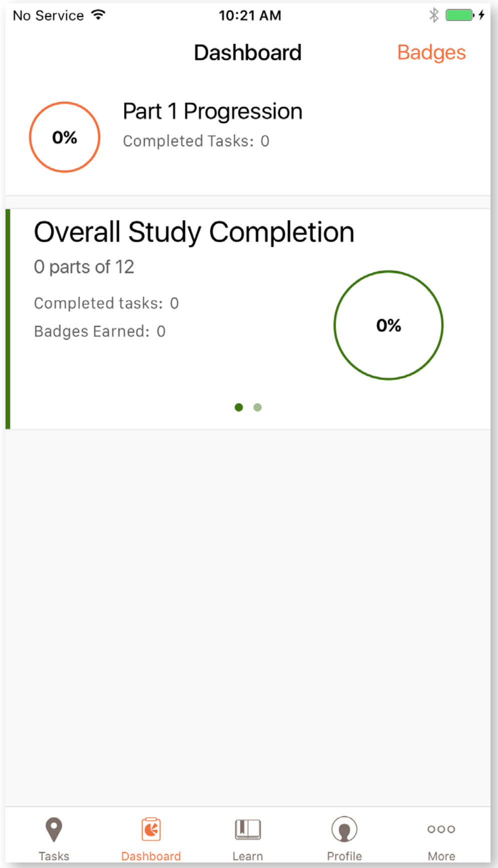

Dashboard Group B (cont.)

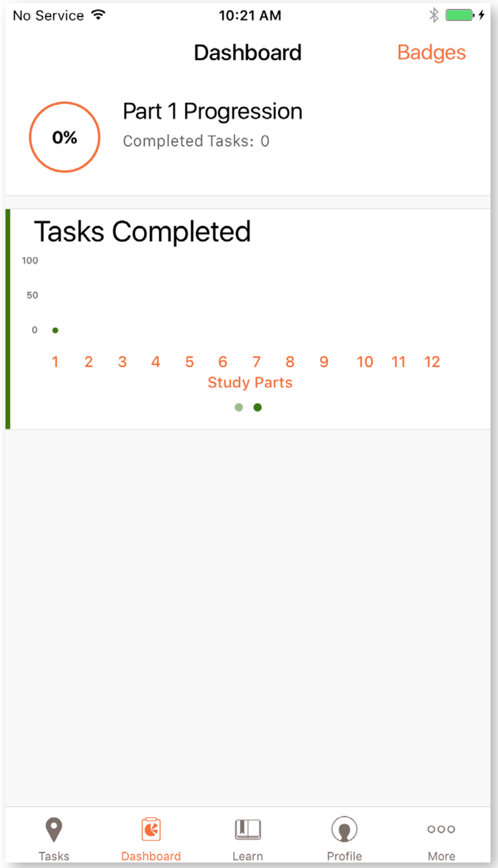

Dashboard Group A

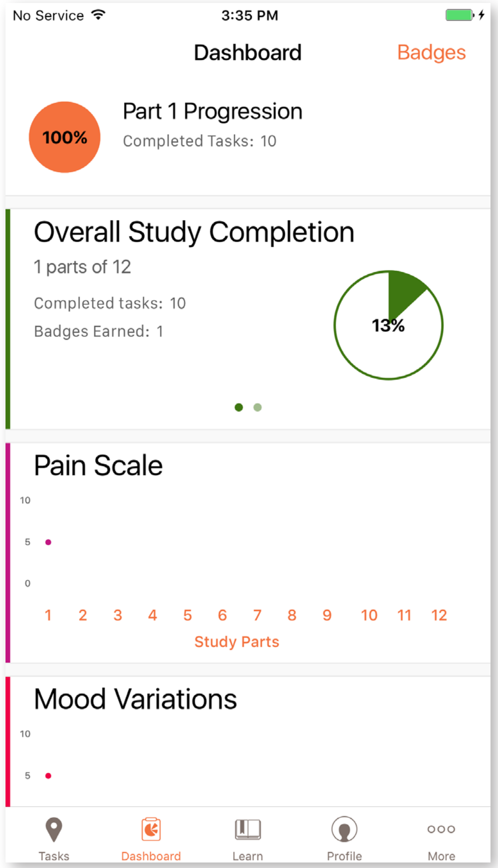

Dashboard Grou A (cont.)

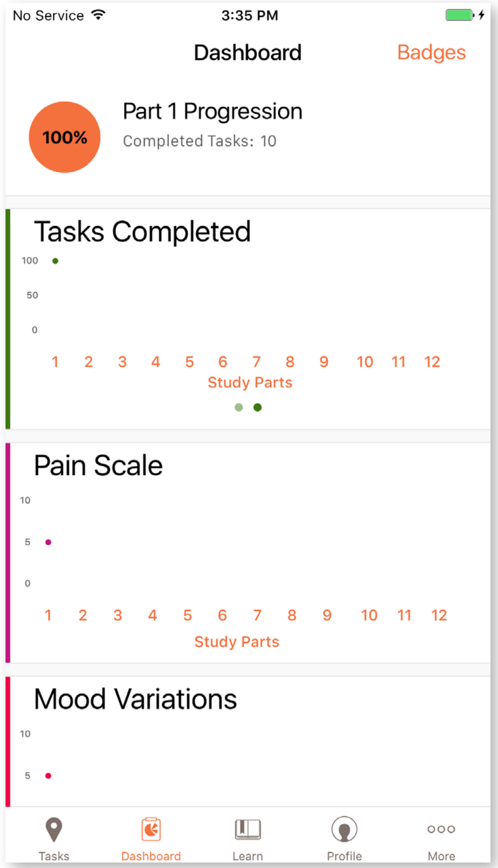

Dashboard Screens, 2

POSSIBLE mobile

Dashboard Group A (cont.)

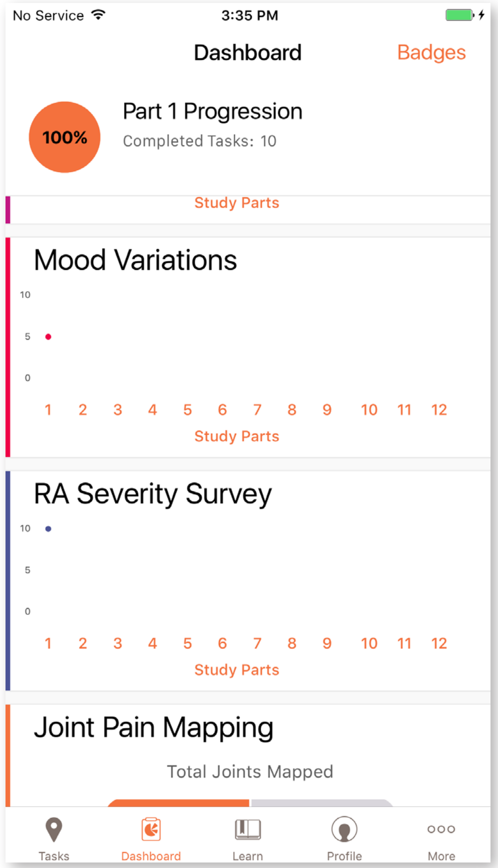

Dashboard Group A (cont.)

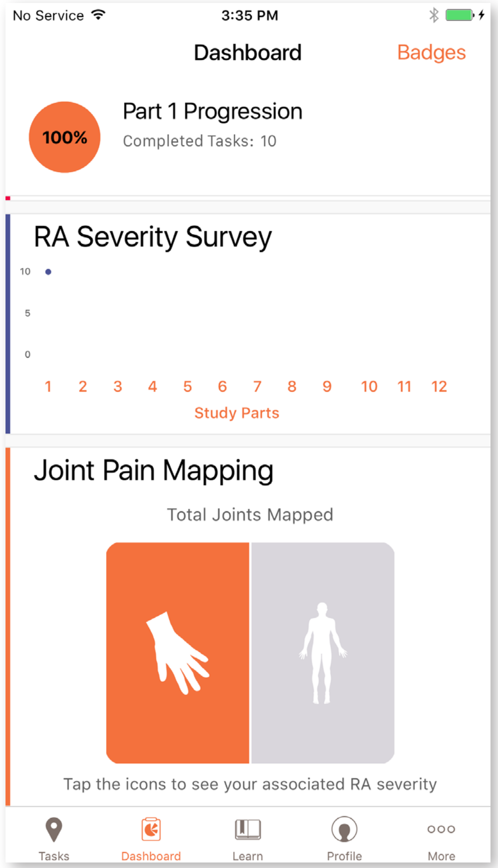

Dashboard Group A (cont.)

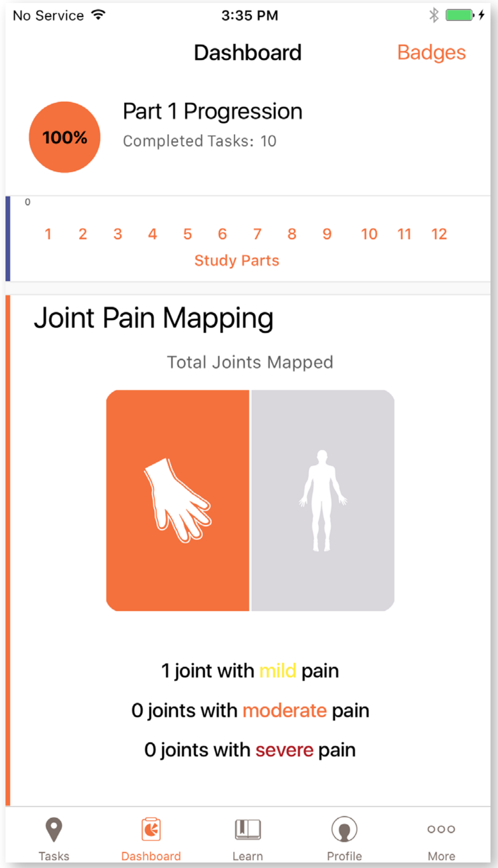

Dashboard Group A (cont.)

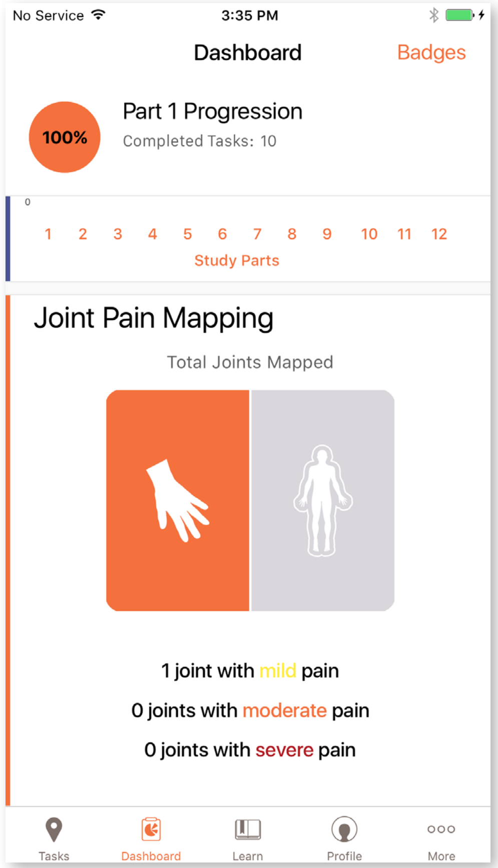

Learn Screens

POSSIBLE mobile

Learn Screen

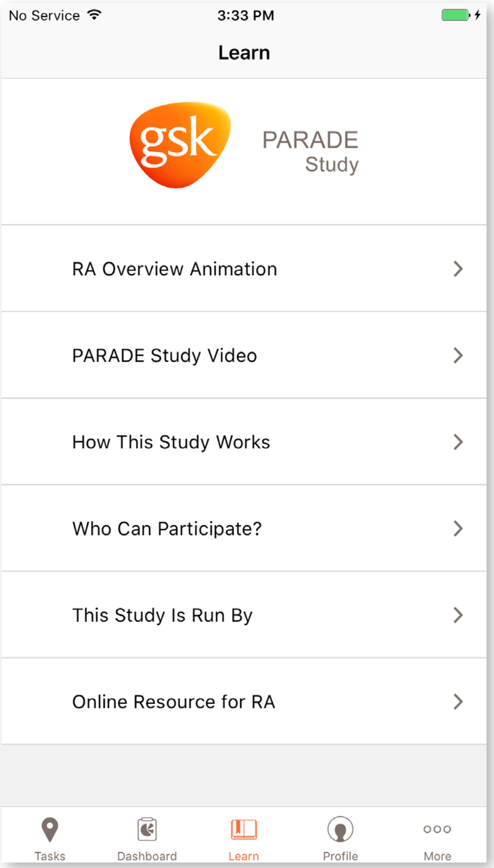

RA Overview Animation Screen

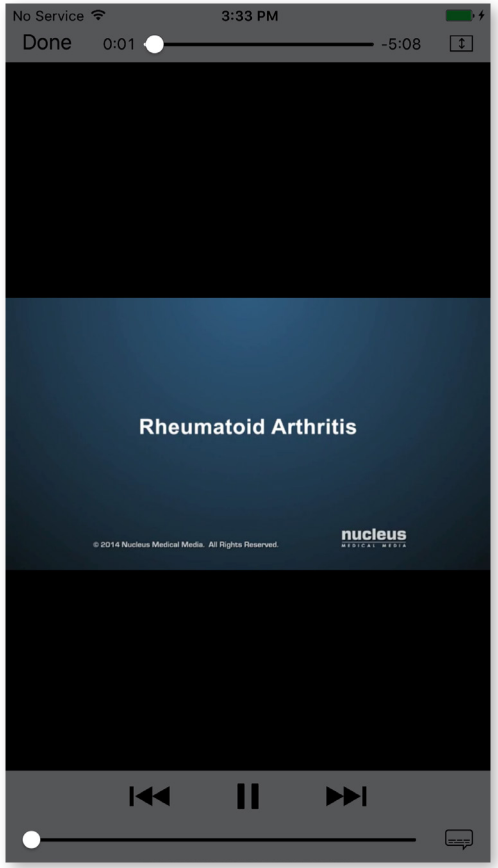

PARADE Study Video

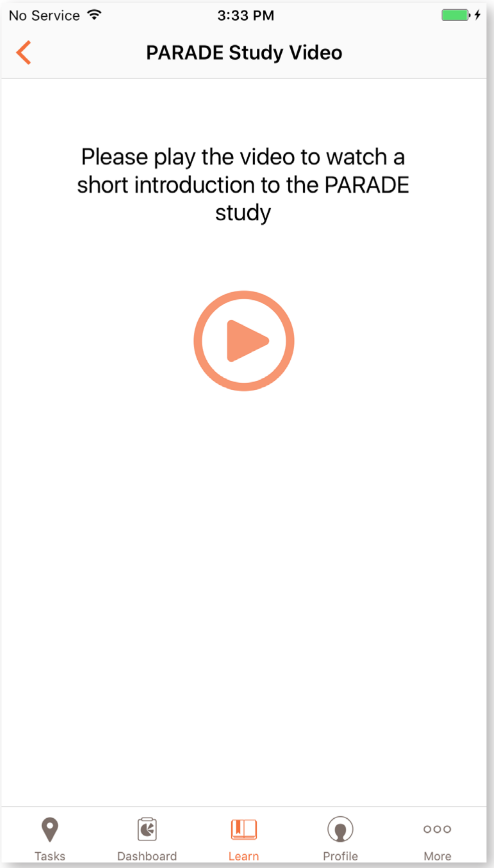

How This Study Works

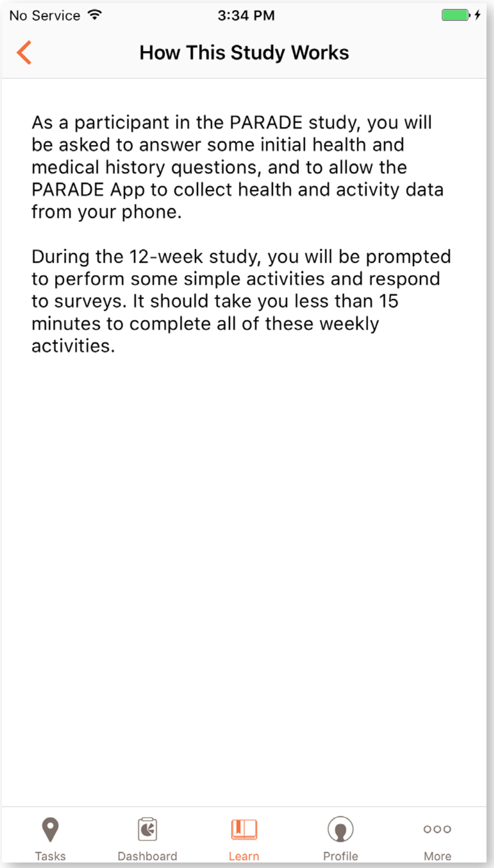

Learn Screens, 2

POSSIBLE mobile

Who Can Particpate

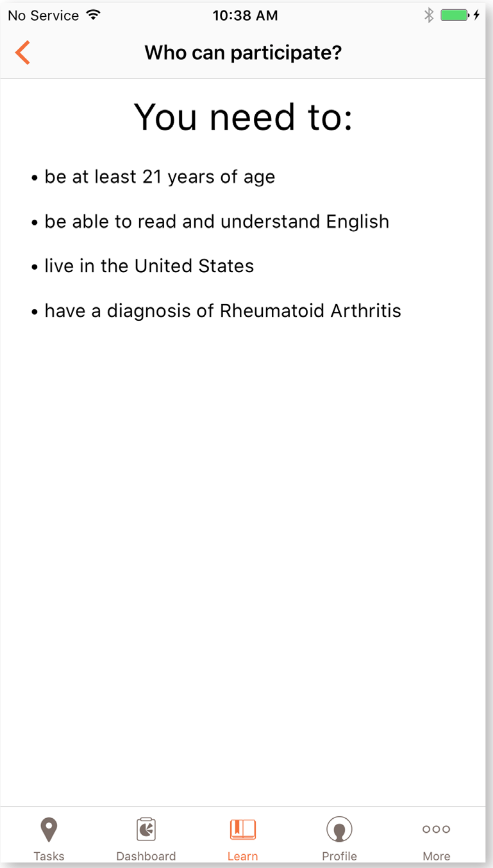

This Study Is Run By

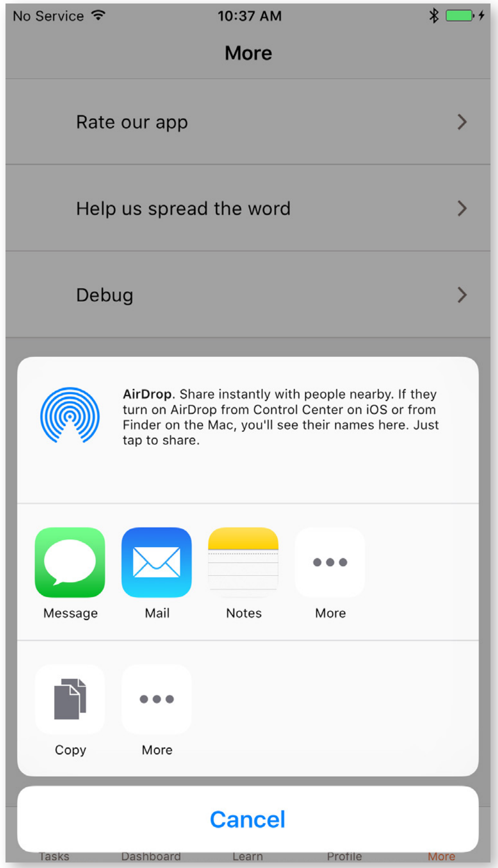

Online Resource for RA

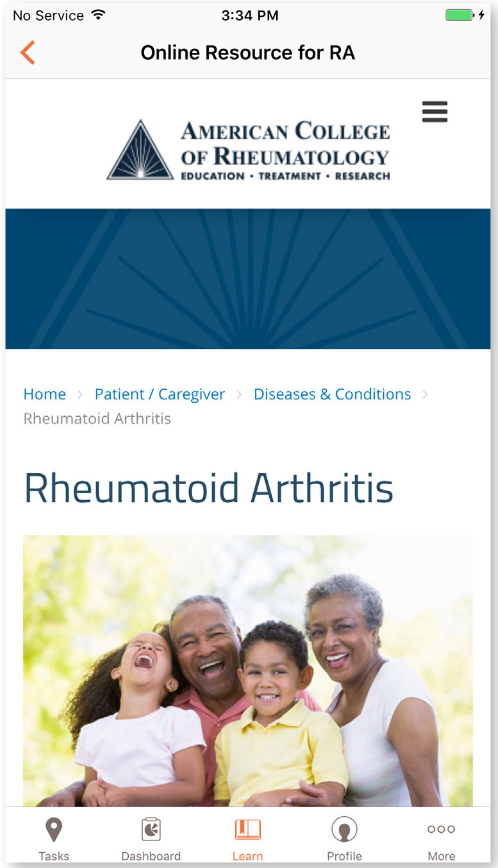

Online Resource for RA (cont.)

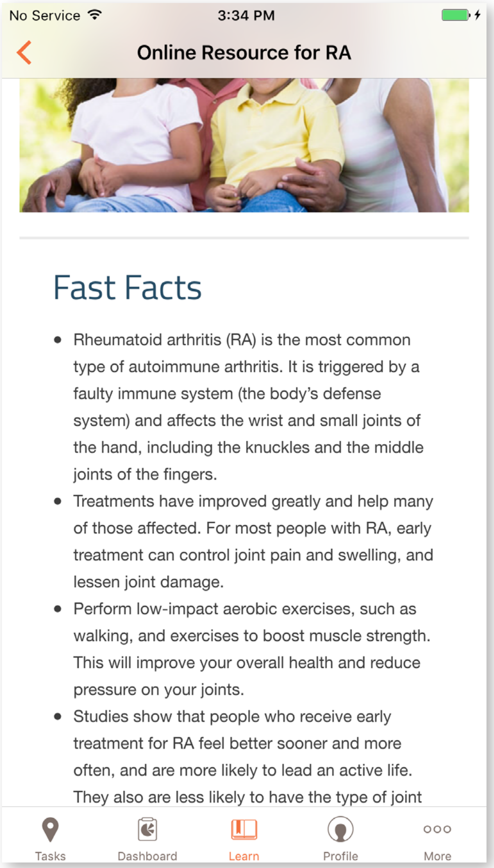

Learn Screens, 3

POSSIBLE mobile

Online Resource for RA (cont.)

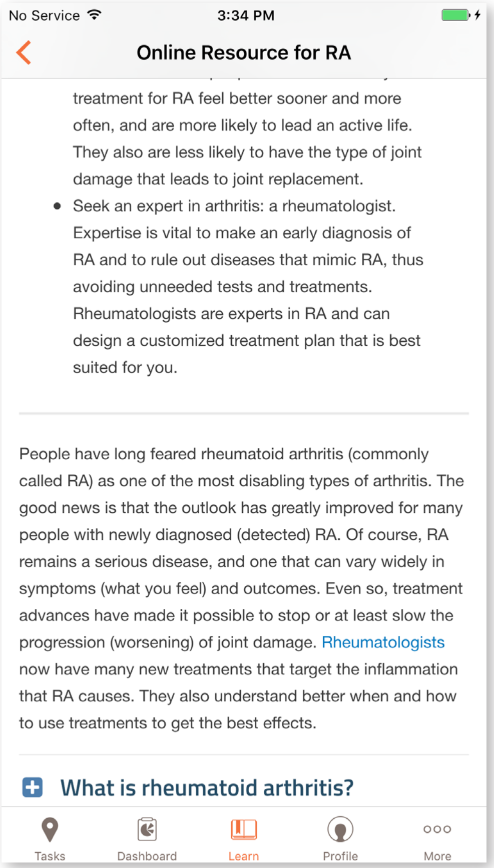

Online Resource for RA (cont.)

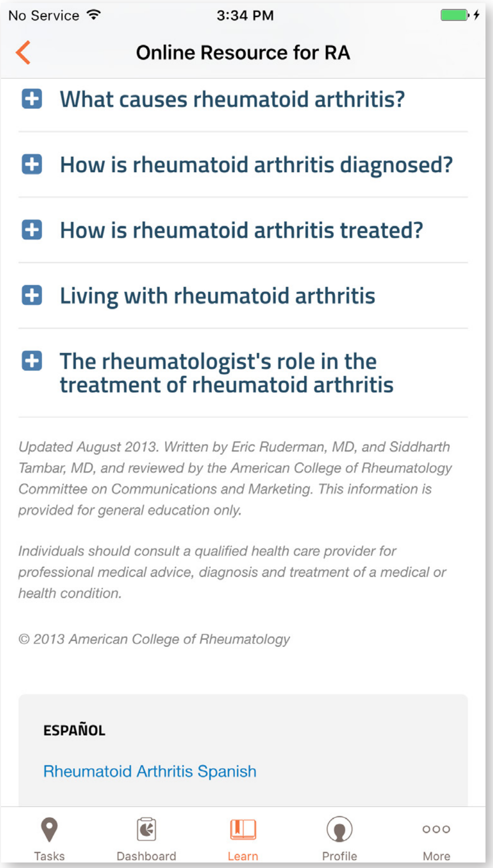

Online Resource for RA (cont.)

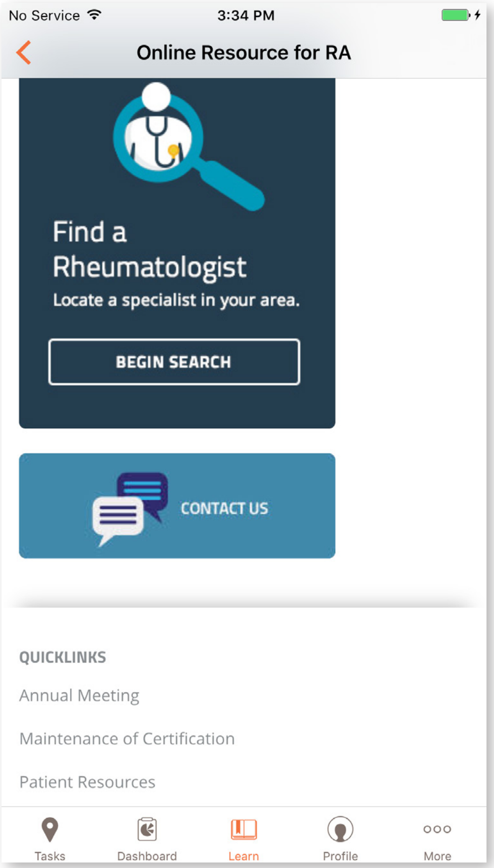

Profile & More Screens

POSSIBLE mobile

Profile View

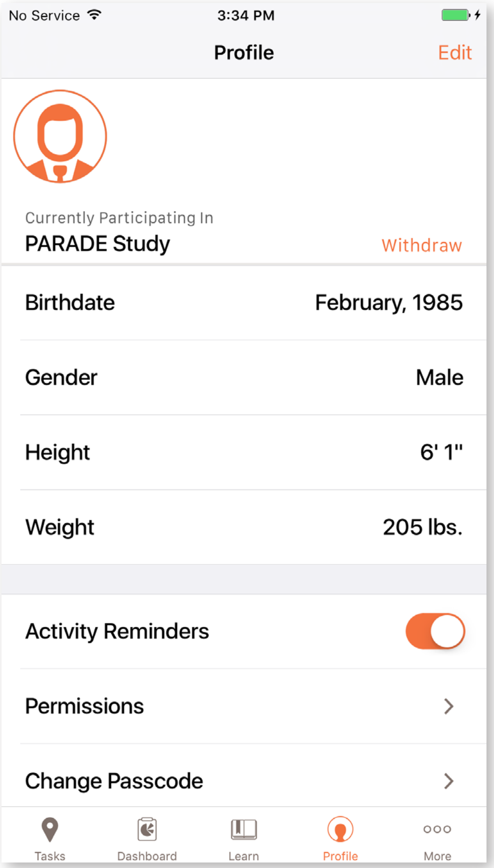

More View

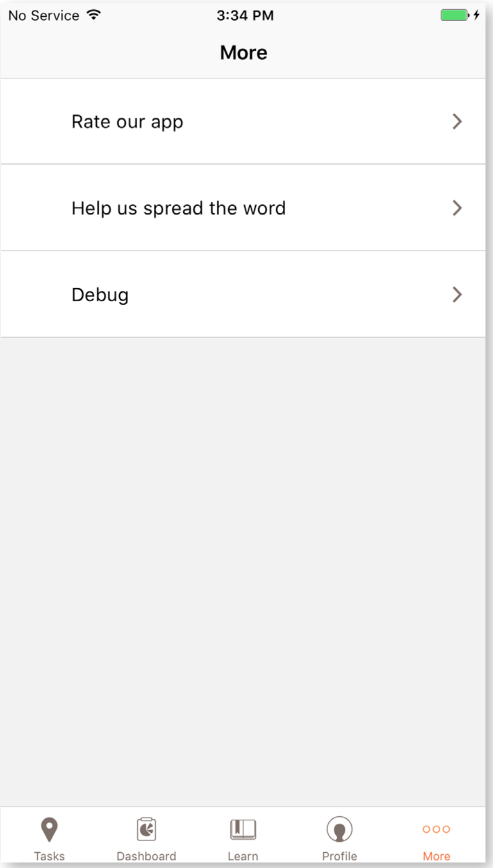

Spread the Word Dialog

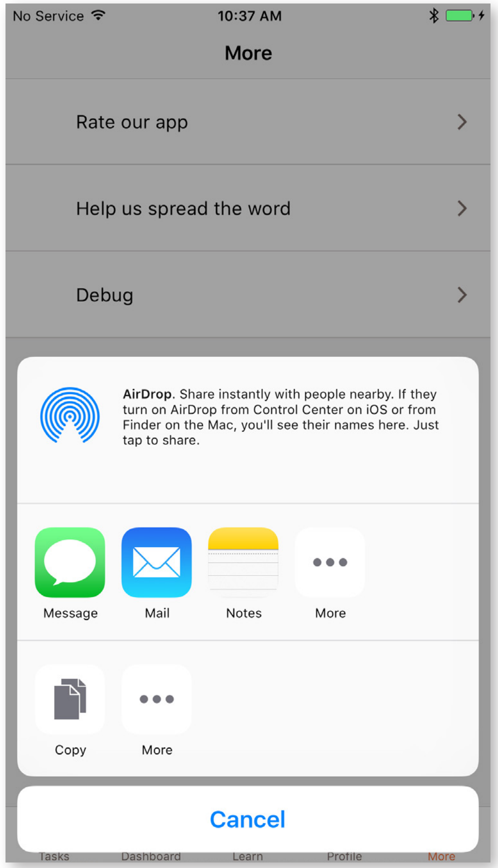

Pain Scale Screens

POSSIBLE mobile

Pain Scale 1

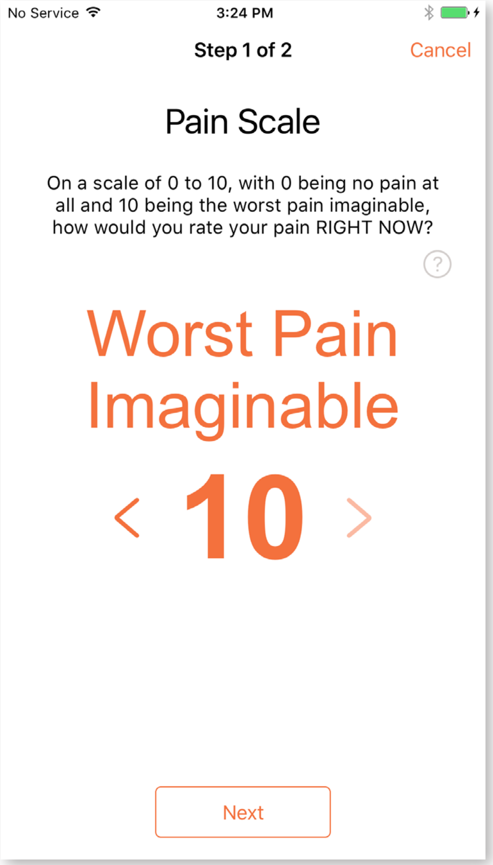

Pain Scale: More Info

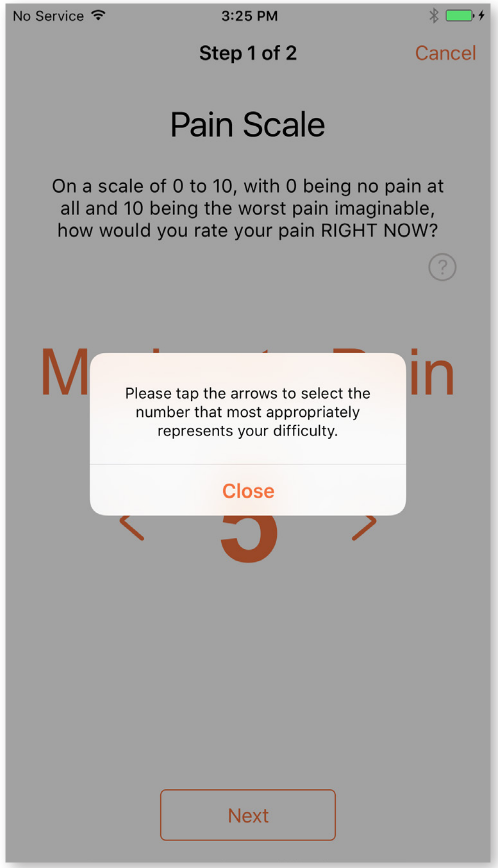

Pain Scale 2

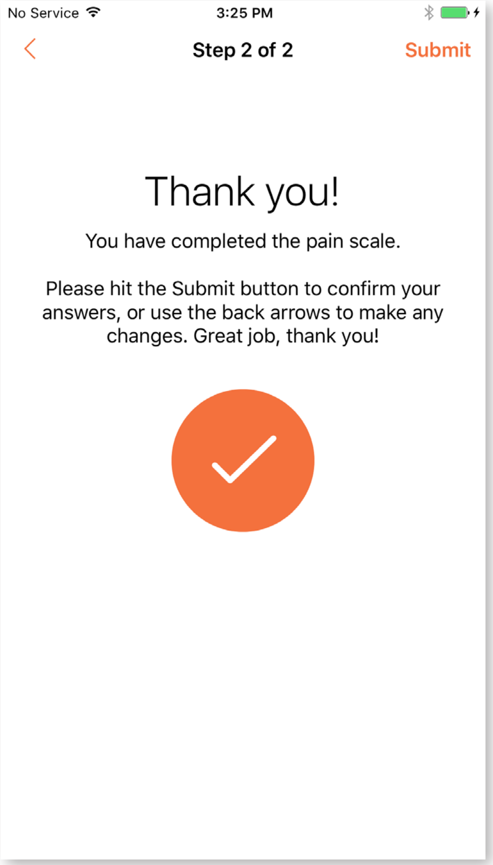

Morning Stiffness Screens

POSSIBLE mobile

Morning Stiffness Survey 1

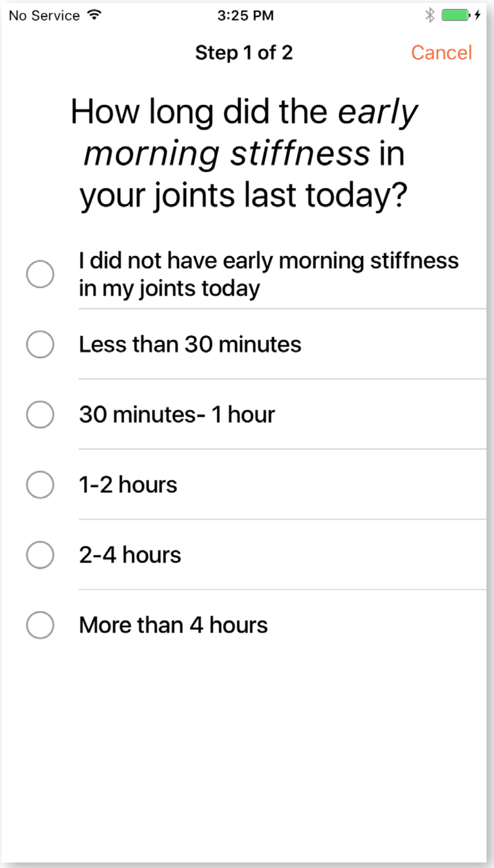

Morning Stiffness Survey 2

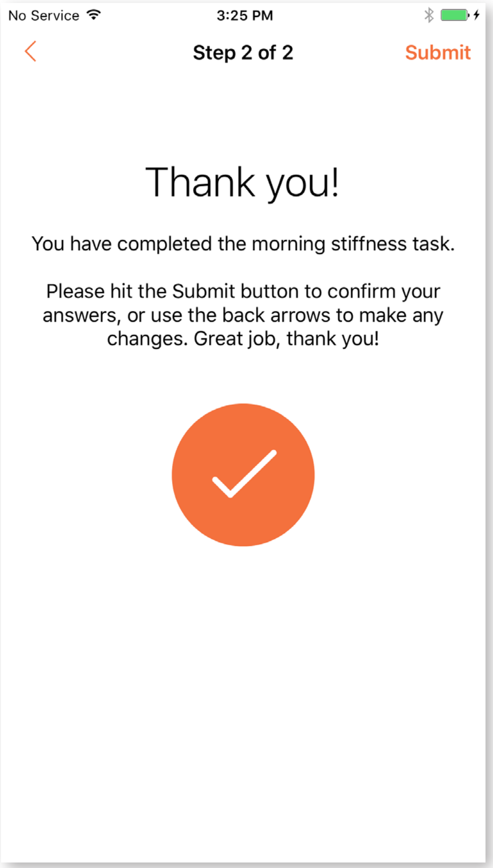

Mood Scale Screens

POSSIBLE mobile

Mood Scale 1

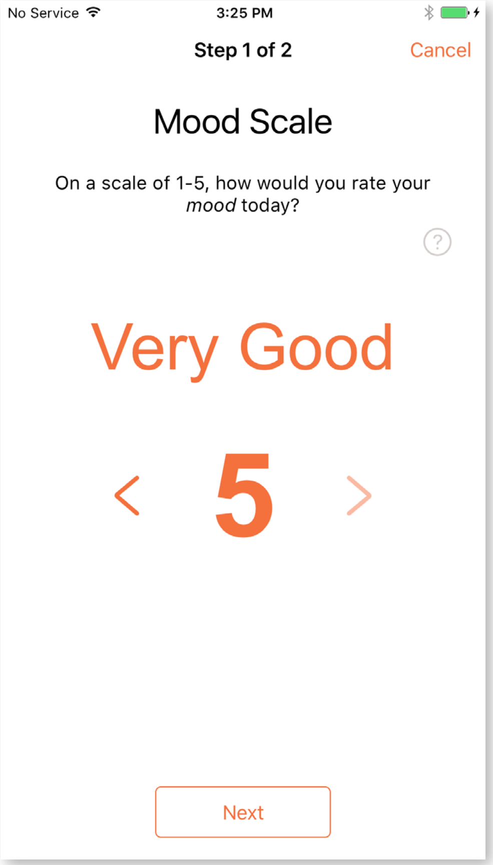

Mood Scale: More Info

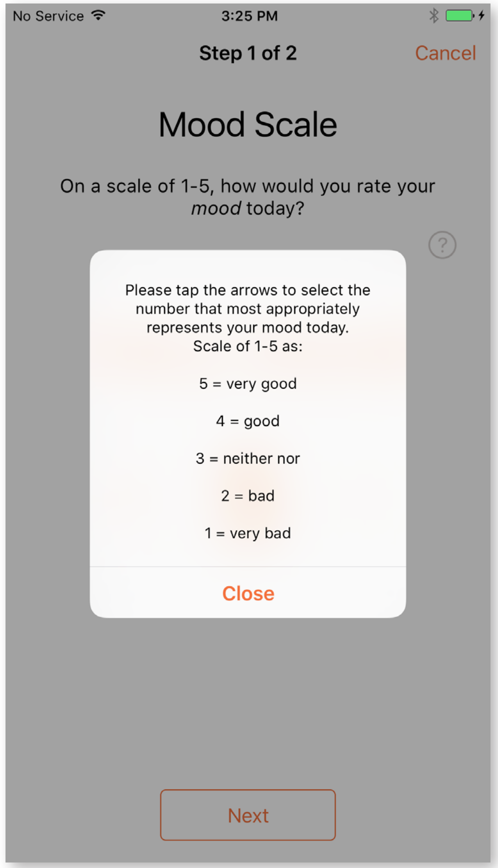

Mood Scale 2

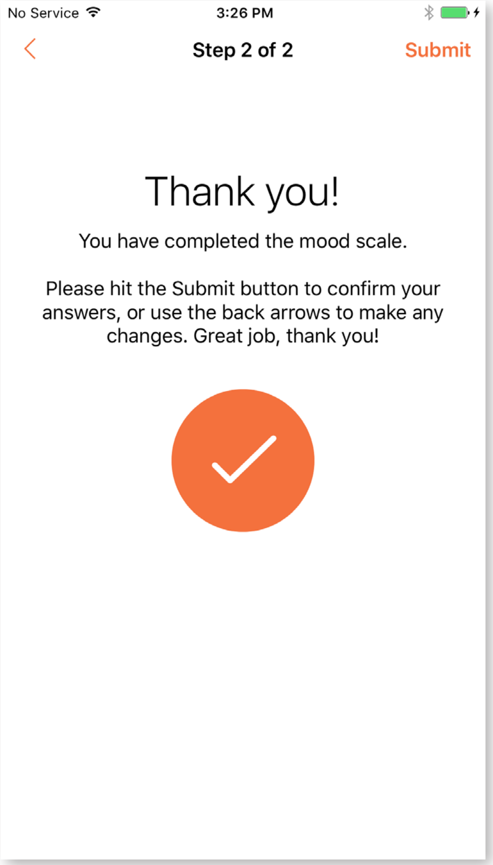

RA Severity Scale Screens

POSSIBLE mobile

RA Severity Scale 1

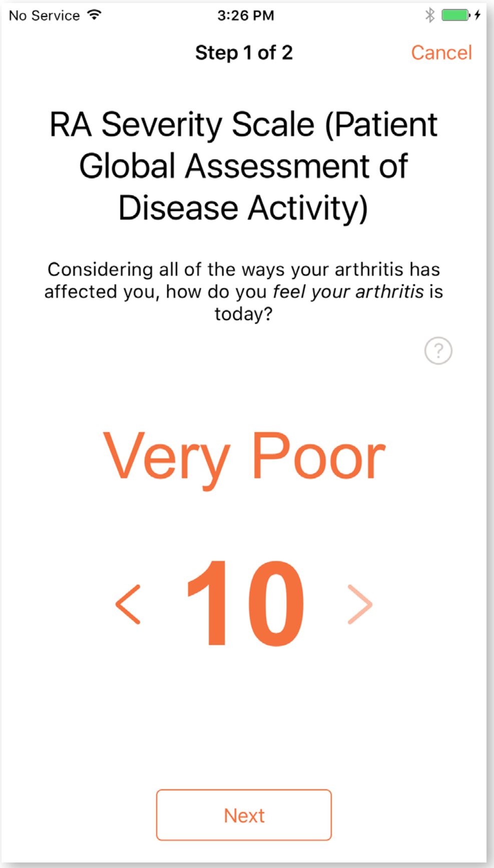

RA Severity Scale: More Info

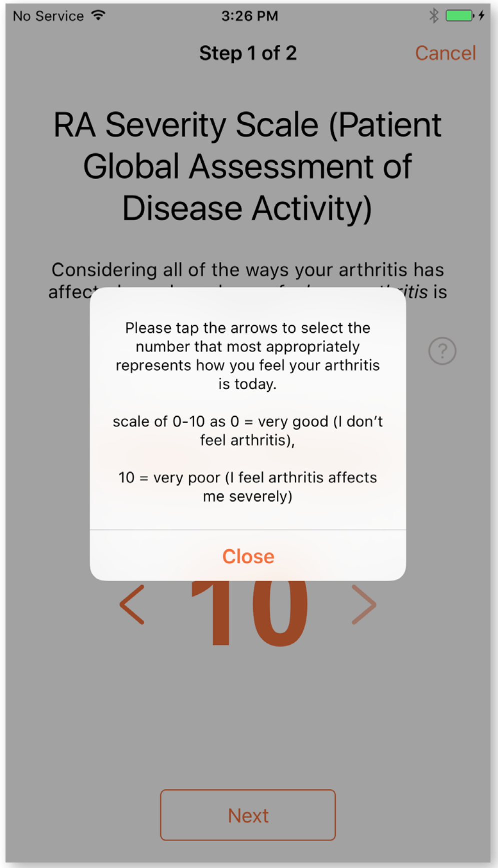

RA Severity Scale 2

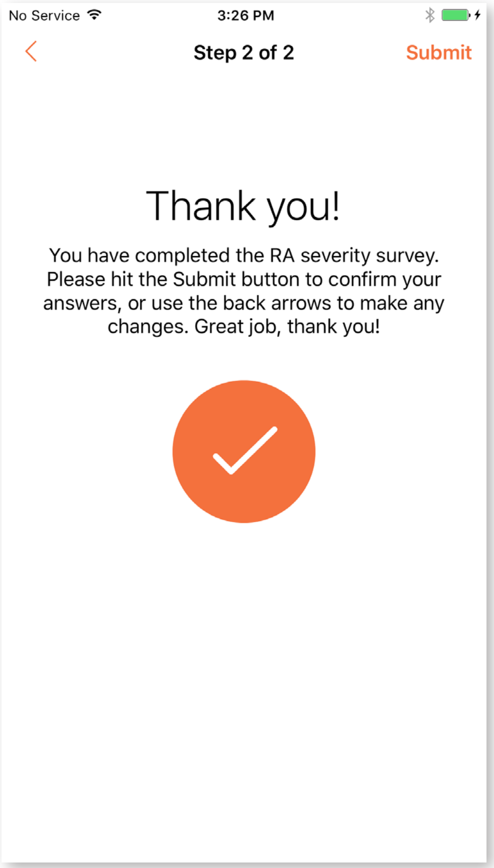

Walk Test Screens

POSSIBLE mobile

Walk Test 1

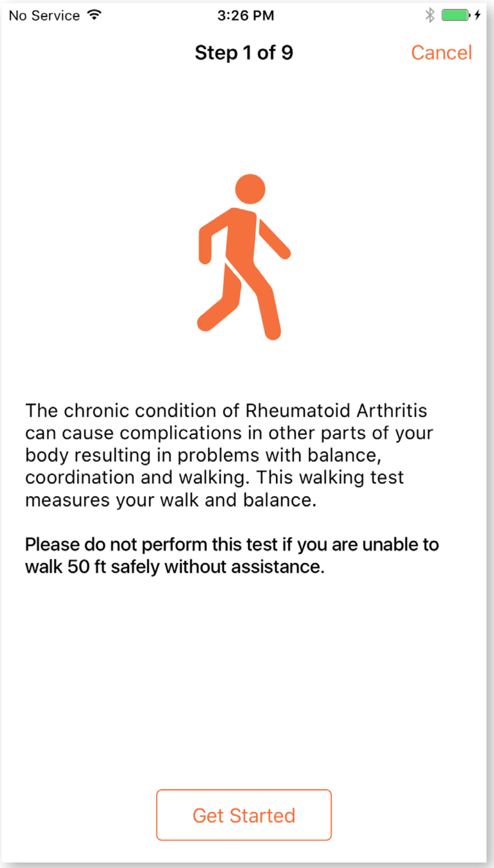

Walk Test 2

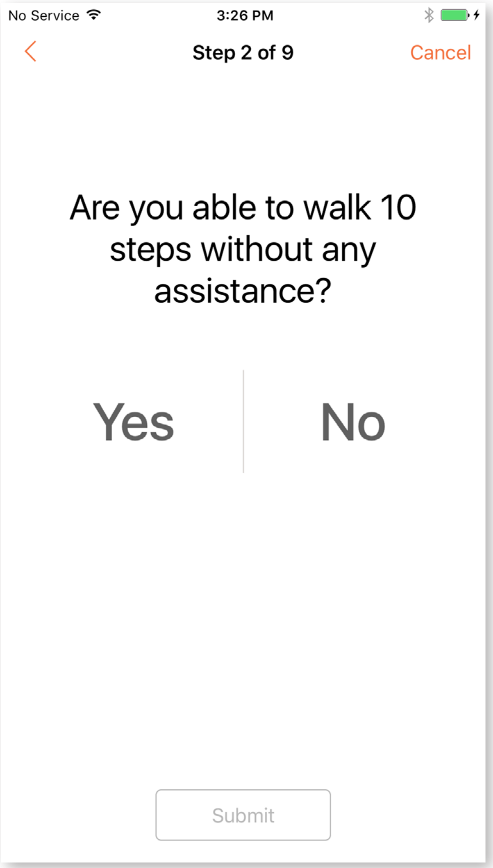

Walk Test: Do Not Need To Complete

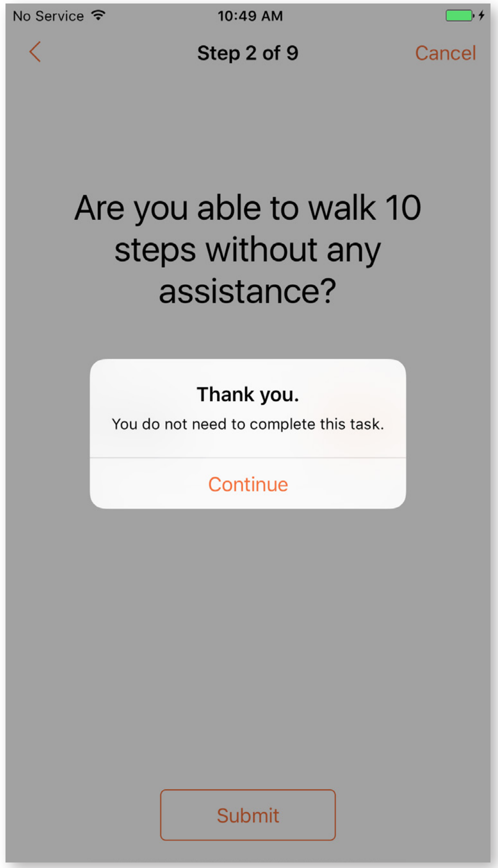

Walk Test 3

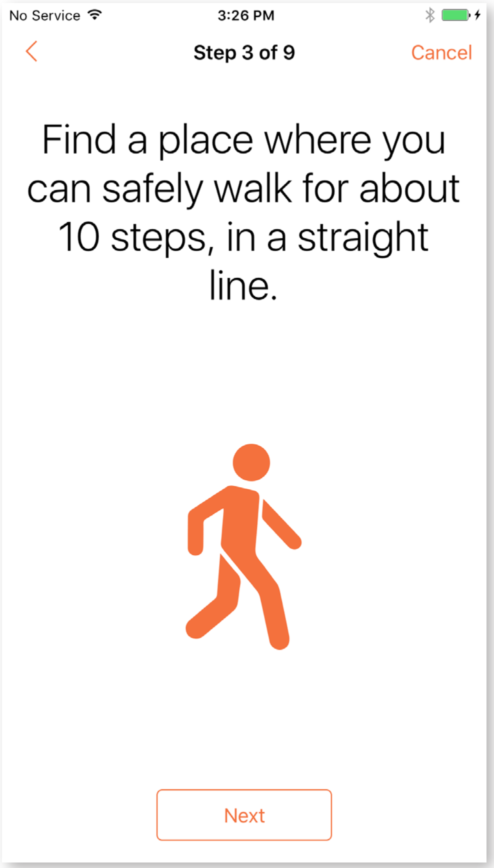

Walk Test Screens, 2

Walk Test 4

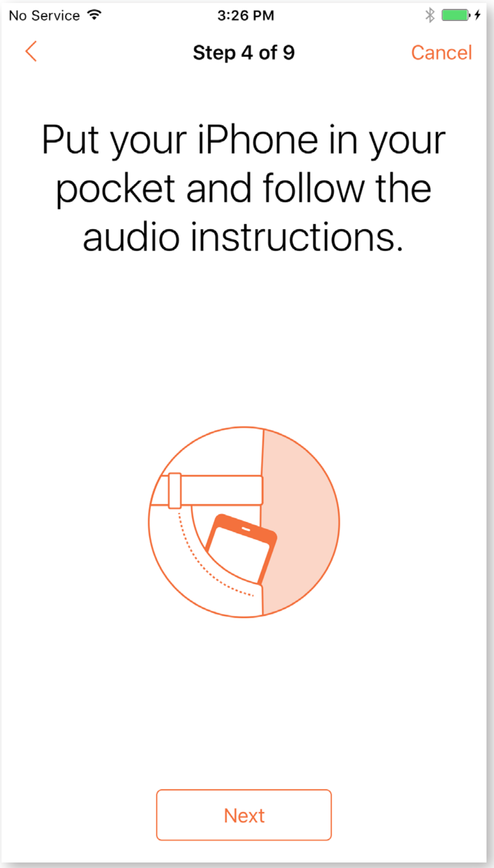

Walk Test 5

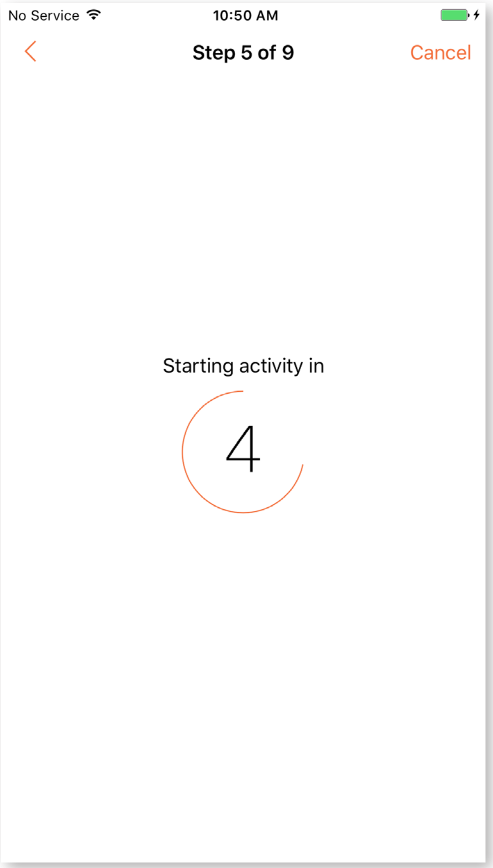

Walk Test 6

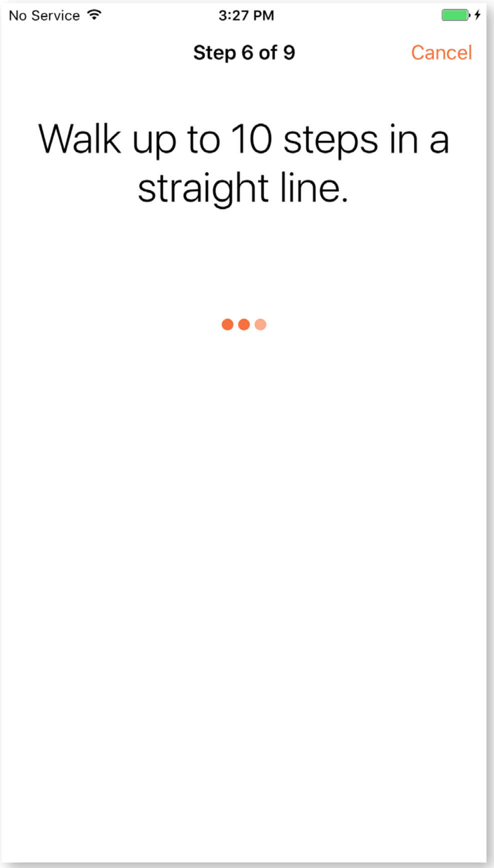

Walk Test 7

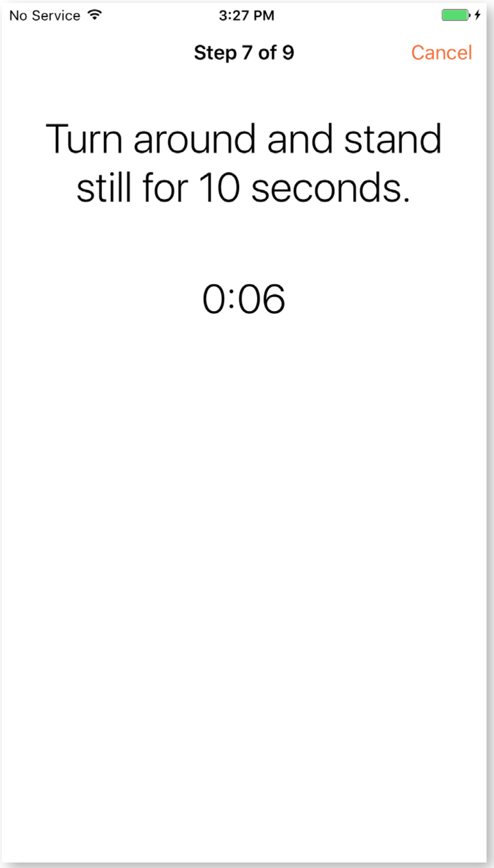

Walk Test Screens, 3

POSSIBLE mobile

Walk Test 8

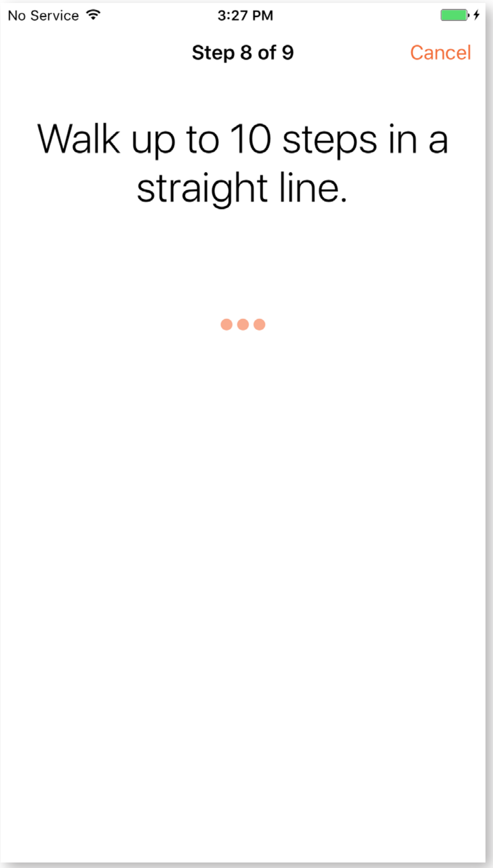

Walk Test 9

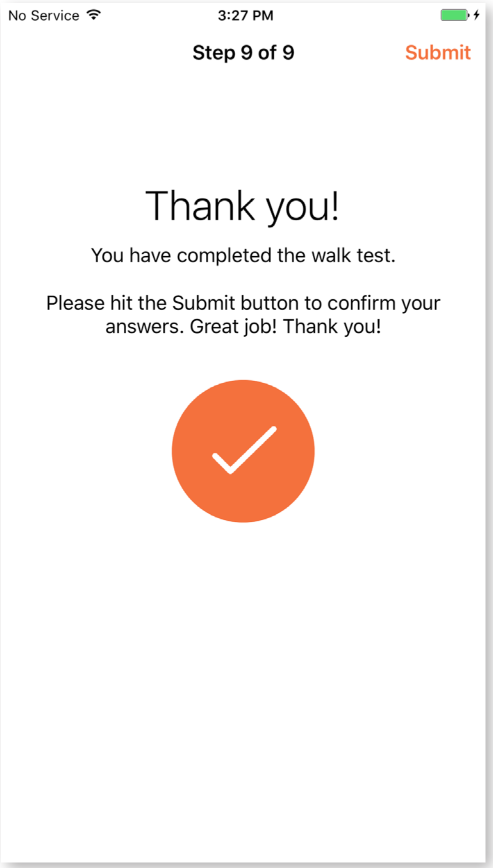

Health Status Survey Screens

POSSIBLE mobile

EQ-5D-5L 1

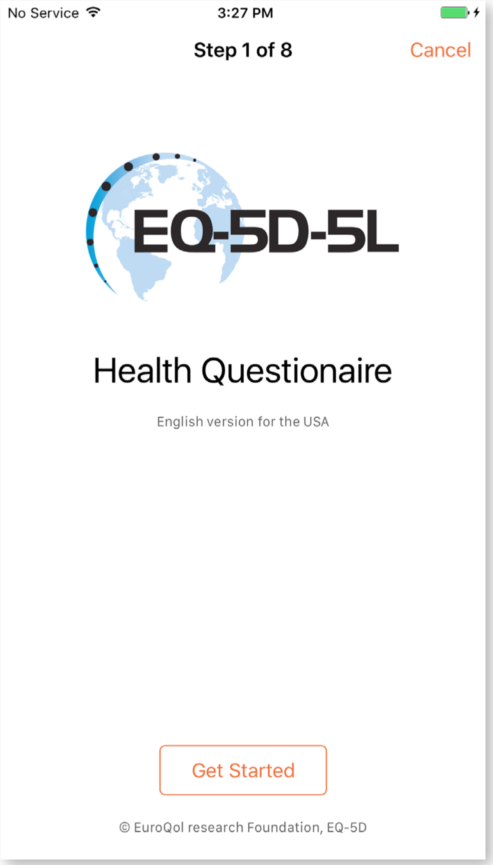

EQ-5D-5L 2

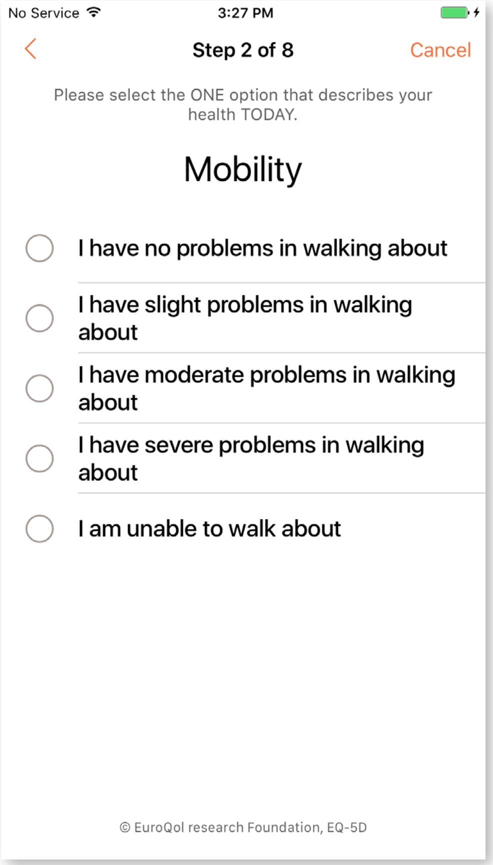

EQ-5D-5L 3

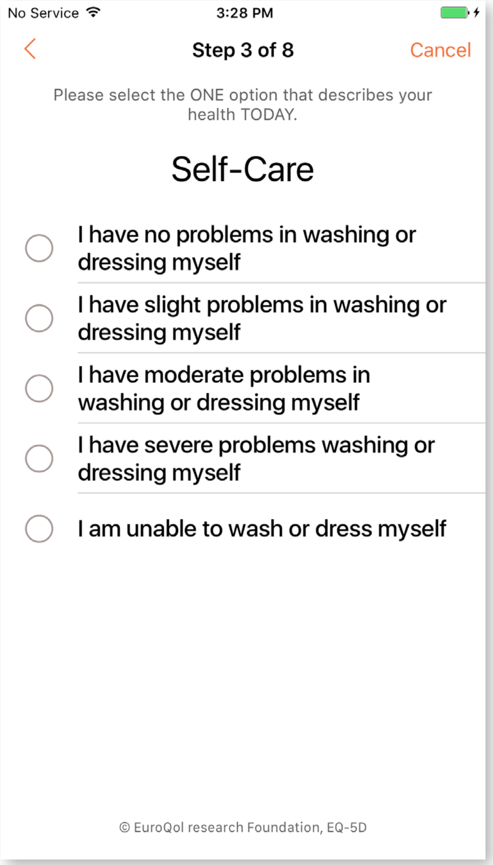

EQ-5D-5L 4

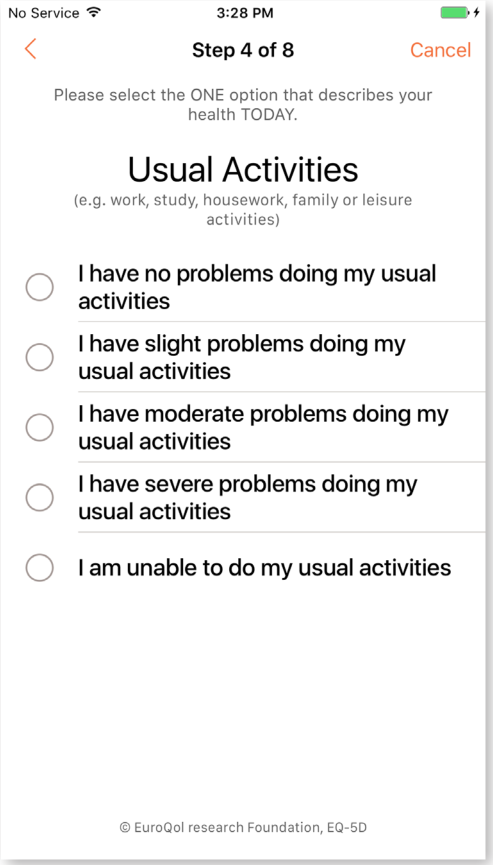

Health Status Survey Screens, 2

POSSIBLE mobile

EQ-5D-5L 5

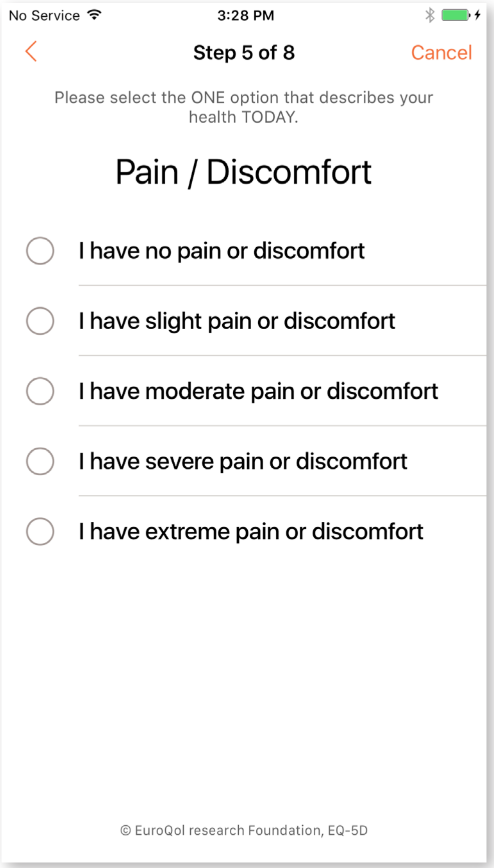

EQ-5D-5L 6

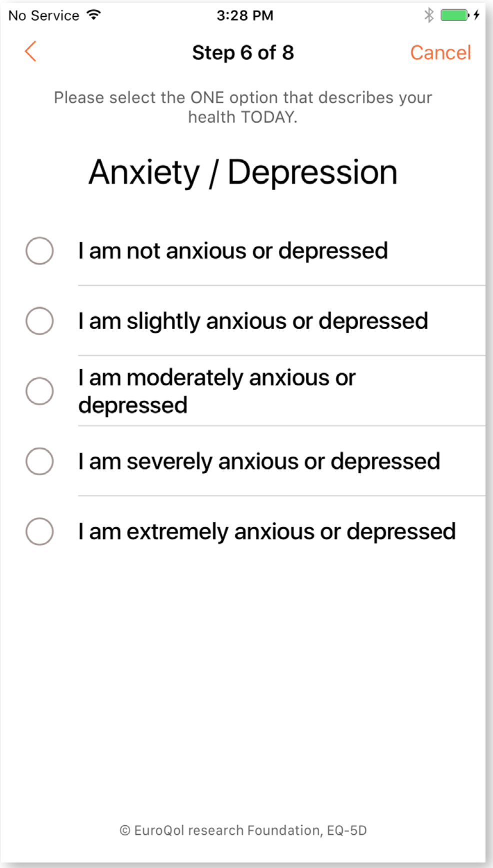

EQ-5D-5L 7

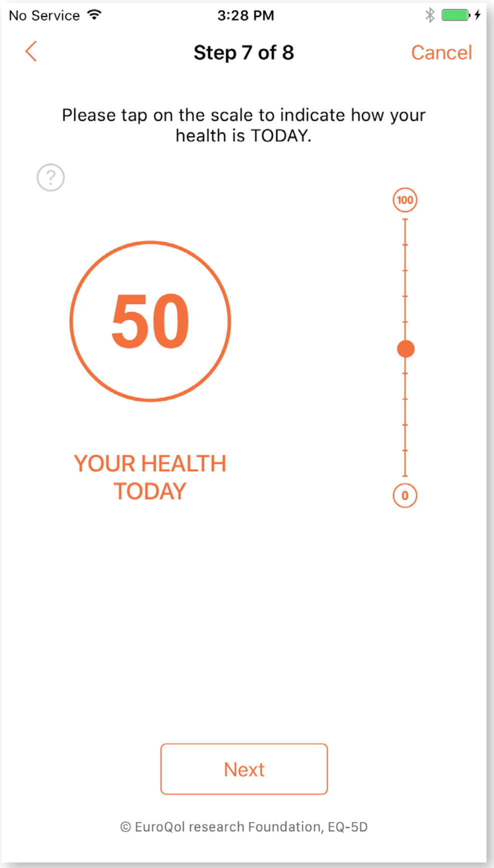

EQ-5D-5L 8

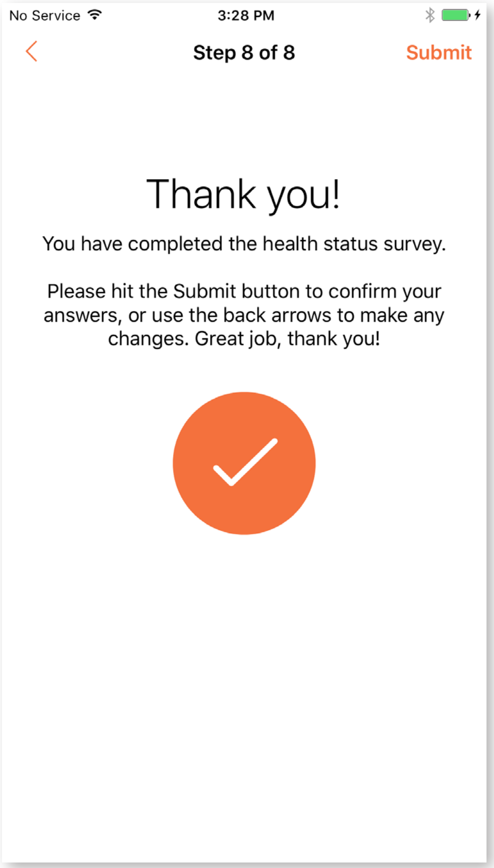

HAQ Screens

POSSIBLE mobile

Physical Function Assessment (HAQ) 1

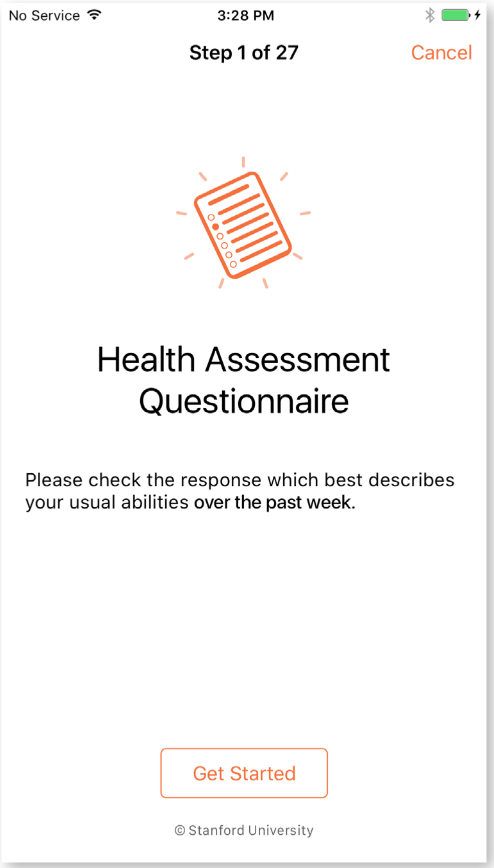

Physical Function Assessment (HAQ) 2

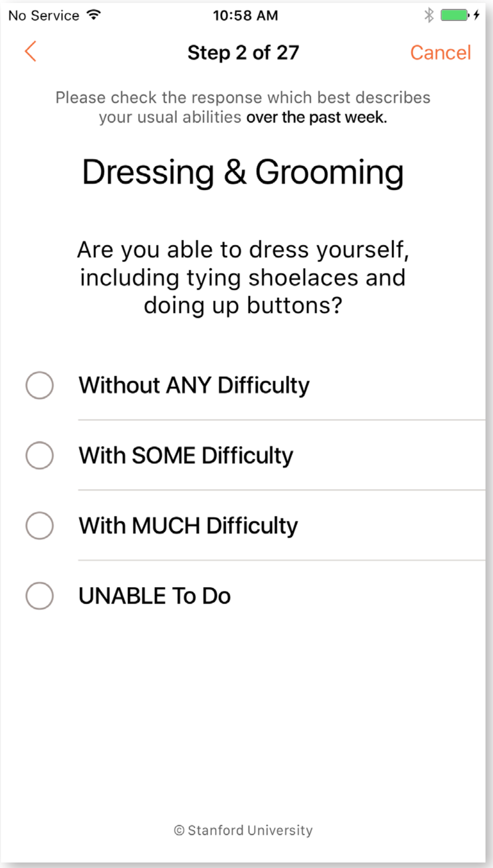

Physical Function Assessment (HAQ) 3

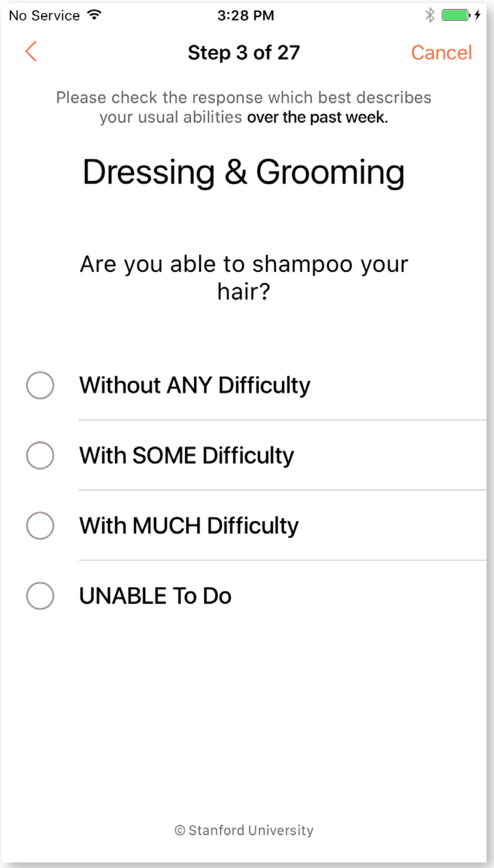

Physical Function Assessment (HAQ) 4

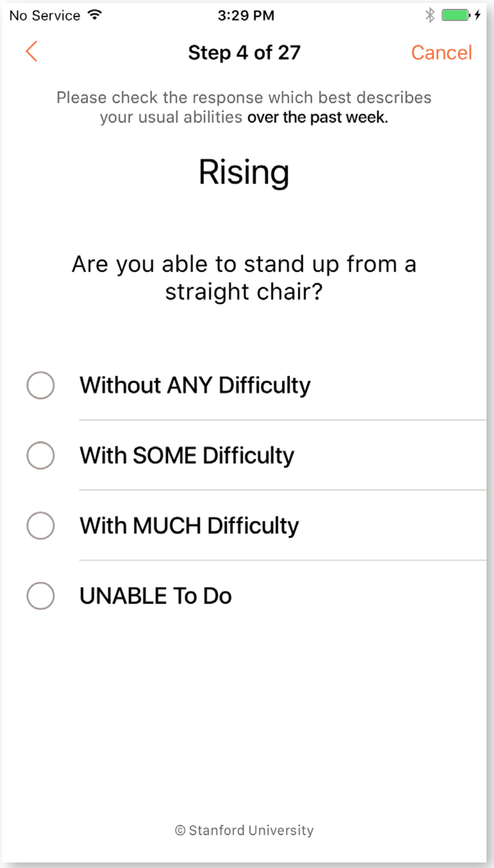

HAQ Screens, 2

POSSIBLE mobile

Physical Function Assessment (HAQ) 5

No Service 3:29 PM

Step 5 of 27 Cancel

Please check the response which best describes your usual abilities **over the past week.**

Rising

Are you able to get in and out of bed?

- Without ANY Difficulty
- With SOME Difficulty
- With MUCH Difficulty
- UNABLE To Do

© Stanford University

Physical Function Assessment (HAQ) 6

No Service 3:29 PM

Step 6 of 27 Cancel

Please check the response which best describes your usual abilities **over the past week.**

Eating

Are you able to cut up your meat?

- Without ANY Difficulty
- With SOME Difficulty
- With MUCH Difficulty
- UNABLE To Do

© Stanford University

Physical Function Assessment (HAQ) 7

No Service 3:29 PM

Step 7 of 27 Cancel

Please check the response which best describes your usual abilities **over the past week.**

Eating

Are you able to lift a full cup or glass to your mouth?

- Without ANY Difficulty
- With SOME Difficulty
- With MUCH Difficulty
- UNABLE To Do

© Stanford University

Physical Function Assessment (HAQ) 8

No Service 3:29 PM

Step 8 of 27 Cancel

Please check the response which best describes your usual abilities **over the past week.**

Eating

Are you able to open a new milk carton?

- Without ANY Difficulty
- With SOME Difficulty
- With MUCH Difficulty
- UNABLE To Do

© Stanford University

HAQ Screens, 3

POSSIBLE mobile

Physical Function Assessment (HAQ) 9

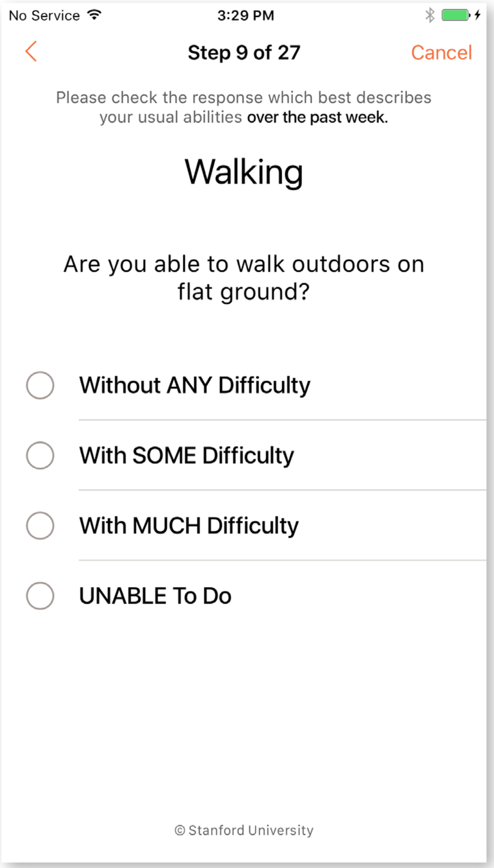

Physical Function Assessment (HAQ) 10

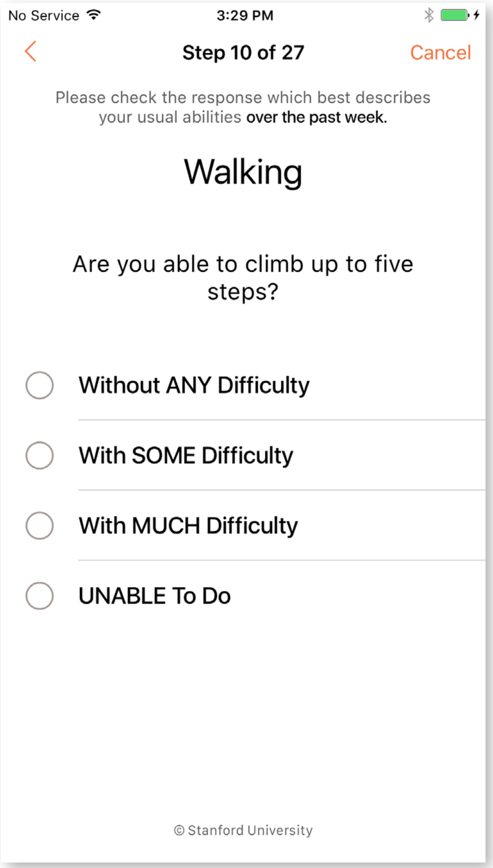

Physical Function Assessment (HAQ) 11

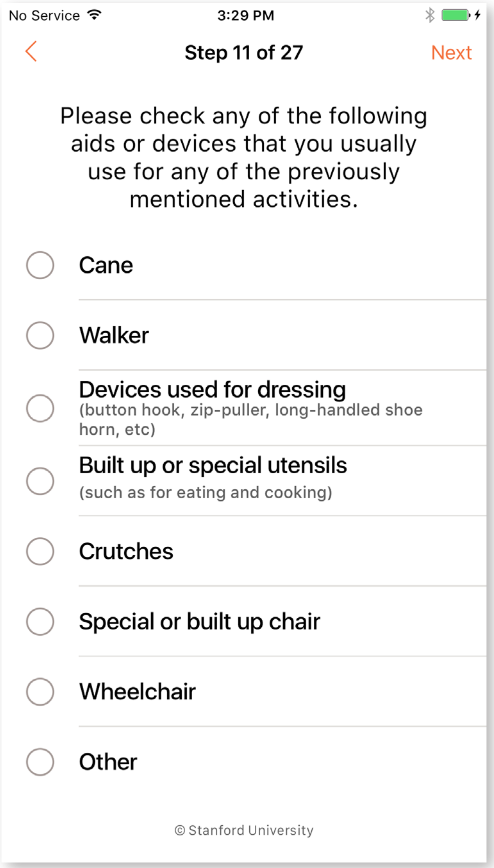

Physical Function Assessment (HAQ) 12

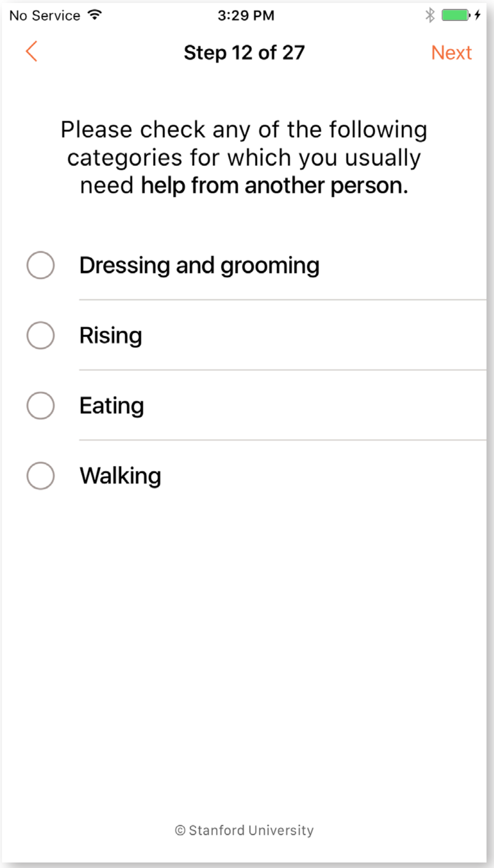

HAQ Screens, 4

POSSIBLE mobile

Physical Function Assessment (HAQ) 13

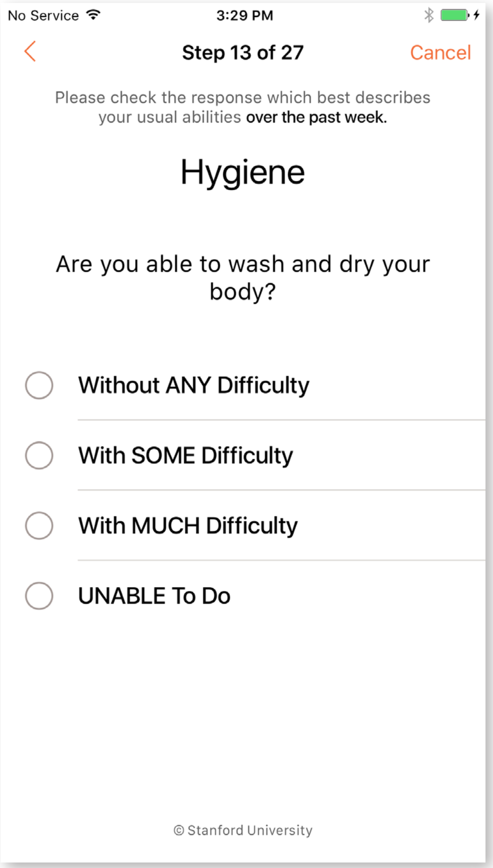

Physical Function Assessment (HAQ) 14

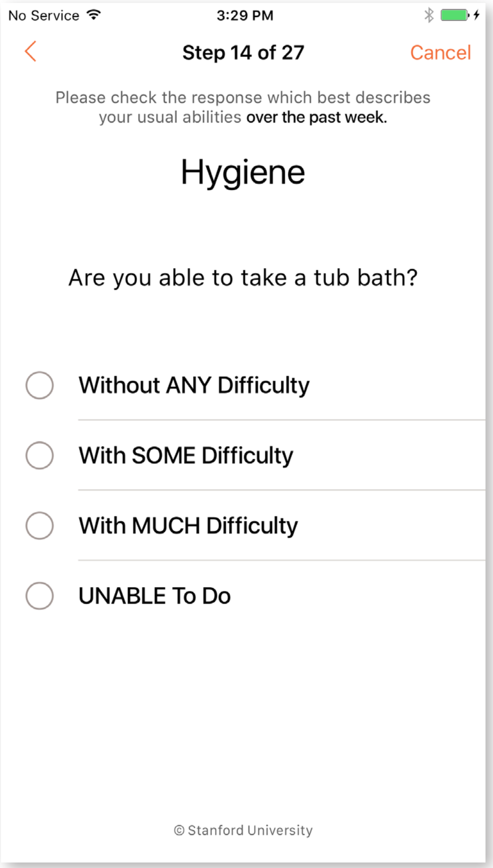

Physical Function Assessment (HAQ) 15

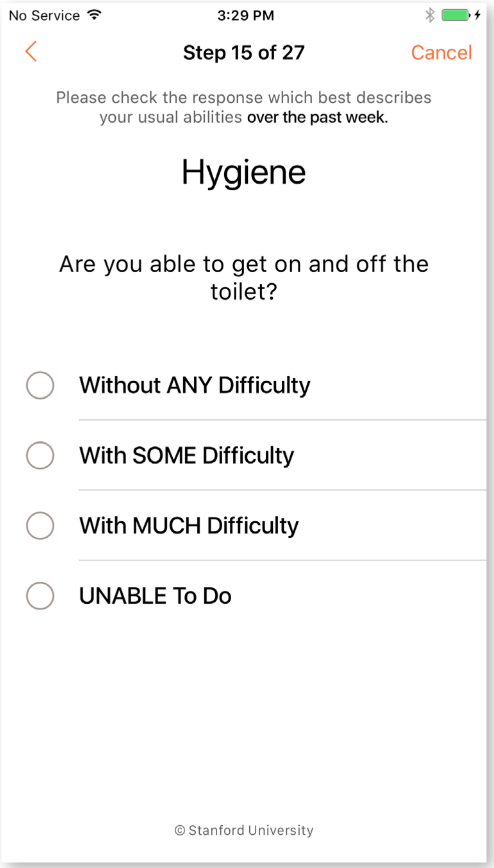

Physical Function Assessment (HAQ) 16

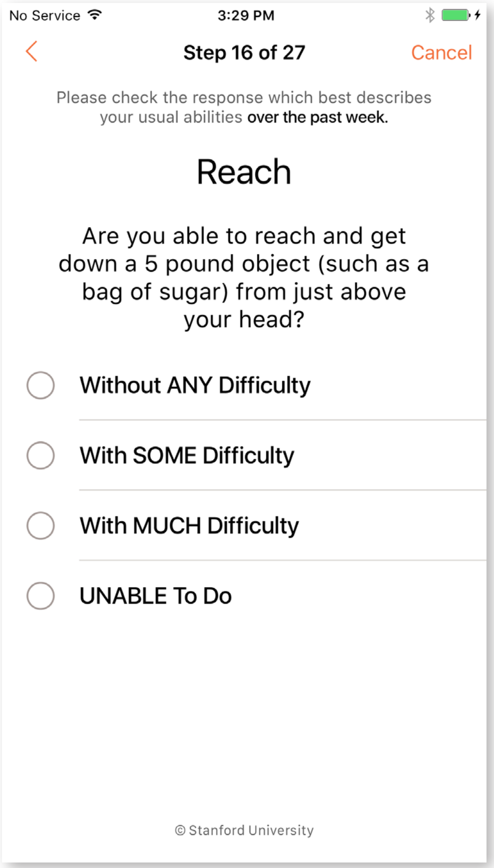

HAQ Screens, 5

POSSIBLE mobile

Physical Function Assessment (HAQ) 17

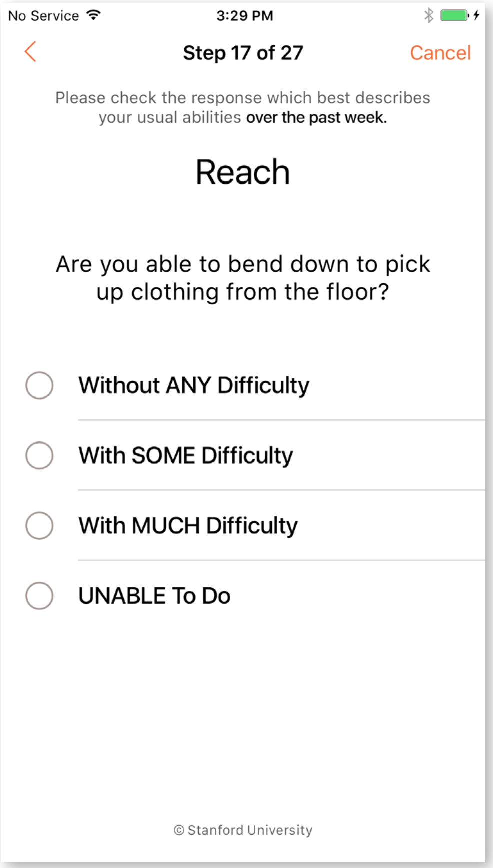

Physical Function Assessment (HAQ) 18

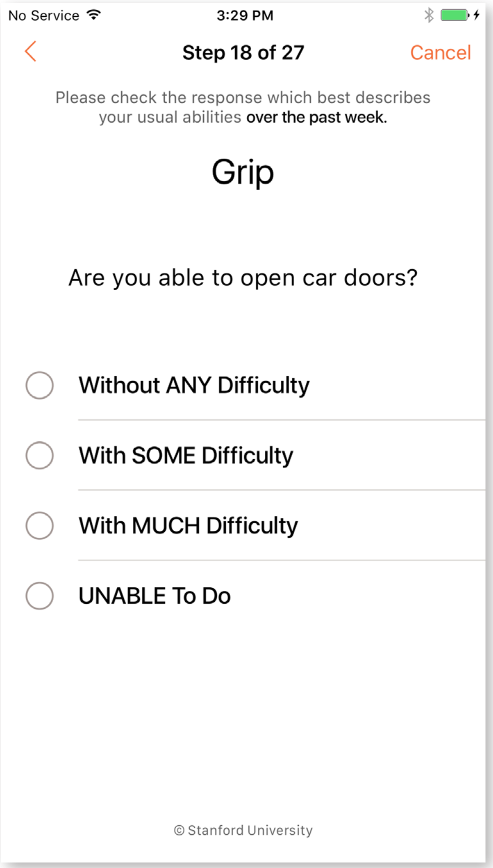

Physical Function Assessment (HAQ) 19

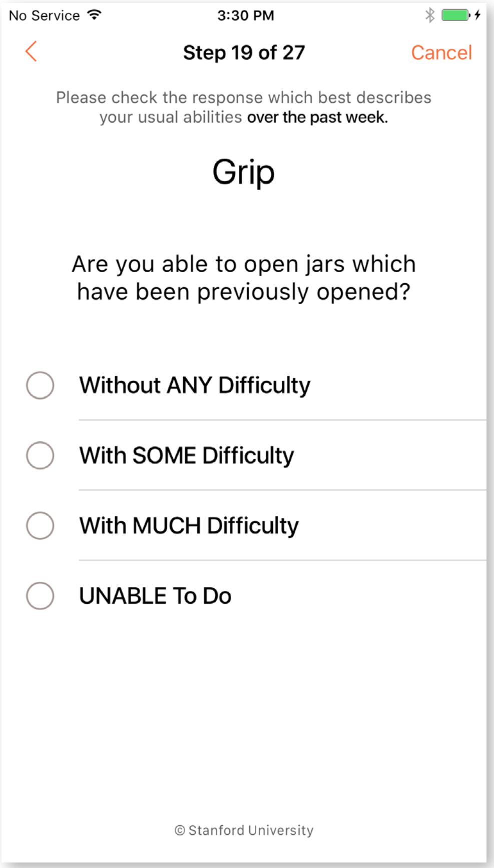

Physical Function Assessment (HAQ) 20

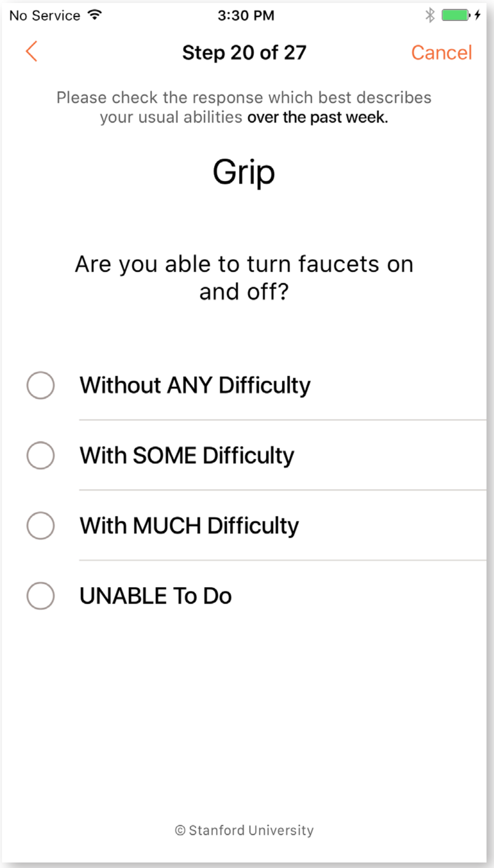

HAQ Screens, 6

POSSIBLE mobile

Physical Function Assessment (HAQ) 21

No Service 3:30 PM

< Step 21 of 27 Cancel

Please check the response which best describes your usual abilities **over the past week.**

Activities

Are you able to run errands and shop?

- Without ANY Difficulty
- With SOME Difficulty
- With MUCH Difficulty
- UNABLE To Do

© Stanford University

Physical Function Assessment (HAQ) 22

No Service 3:30 PM

< Step 22 of 27 Cancel

Please check the response which best describes your usual abilities **over the past week.**

Activities

Are you able to get in and out of a car?

- Without ANY Difficulty
- With SOME Difficulty
- With MUCH Difficulty
- UNABLE To Do

© Stanford University

Physical Function Assessment (HAQ) 23

No Service 3:30 PM

< Step 23 of 27 Cancel

Please check the response which best describes your usual abilities **over the past week.**

Activities

Are you able to do chores such as vacuuming or yardwork?

- Without ANY Difficulty
- With SOME Difficulty
- With MUCH Difficulty
- UNABLE To Do

© Stanford University

Physical Function Assessment (HAQ) 24

No Service 3:30 PM

< Step 24 of 27 Next

Please check any of the following aids or devices that you usually use for any of the previously mentioned activities.

- Raised Toilet Seat
- Bathtub seat
- Jar Opener (for jars previously opened)
- Bathtub bar
- Long-handled appliances for reach
- Long-handled appliances in the bathroom (e.g. a long-handled brush)
- Other

© Stanford University

HAQ Screens, 7

POSSIBLE mobile

Physical Function Assessment (HAQ) 25

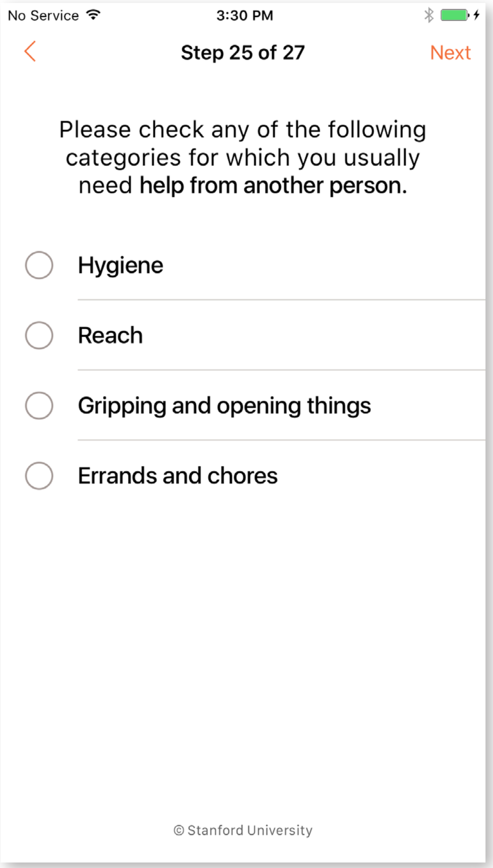

Physical Function Assessment (HAQ) 26

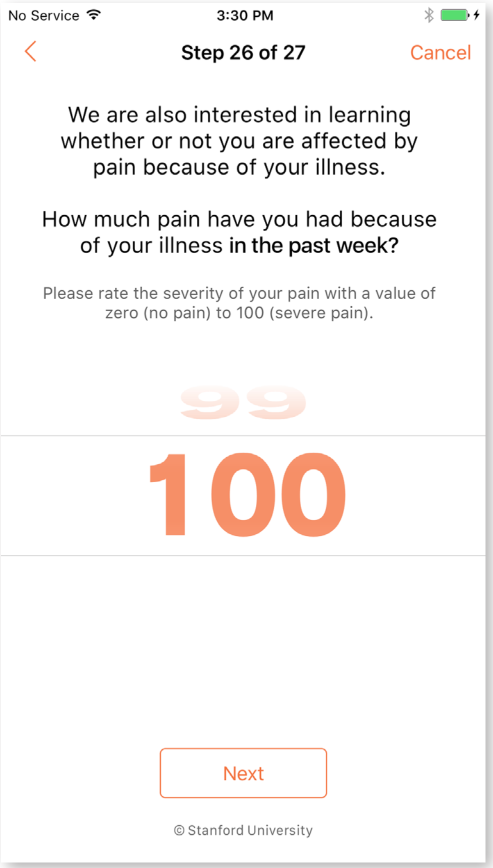

Physical Function Assessment (HAQ) 27

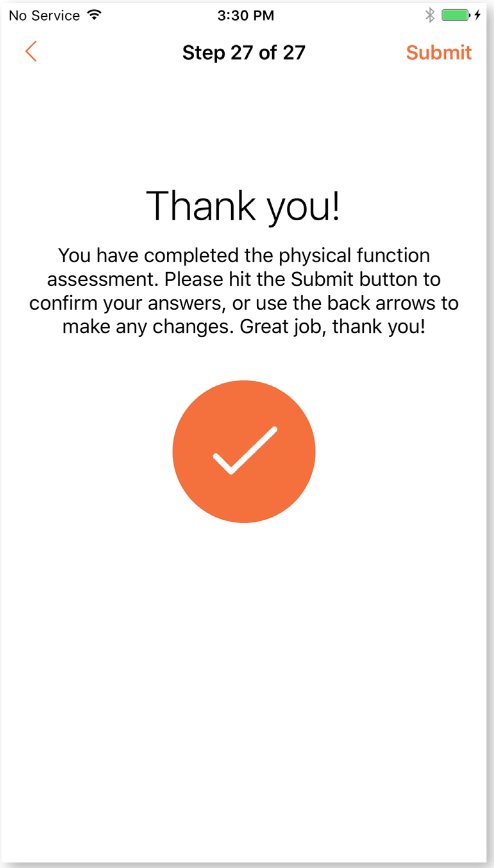

Fatigue Scale Screens

POSSIBLE mobile

Fatigue Scale (FACIT) 1

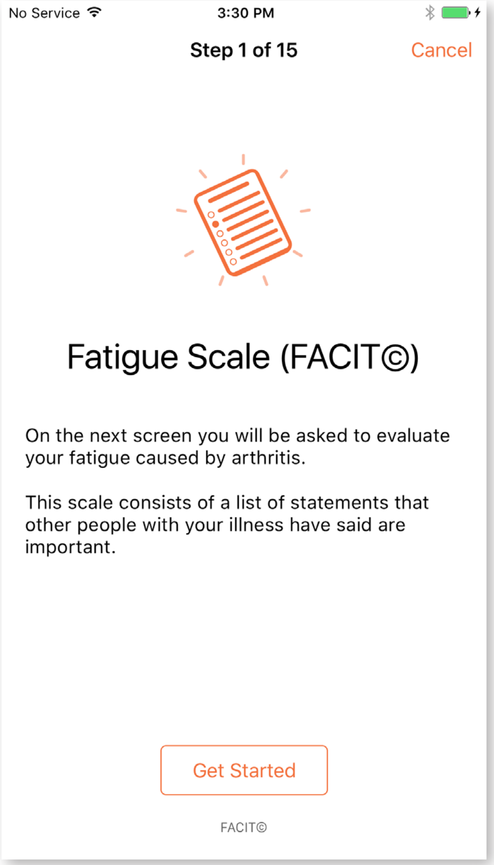

Fatigue Scale (FACIT) 2

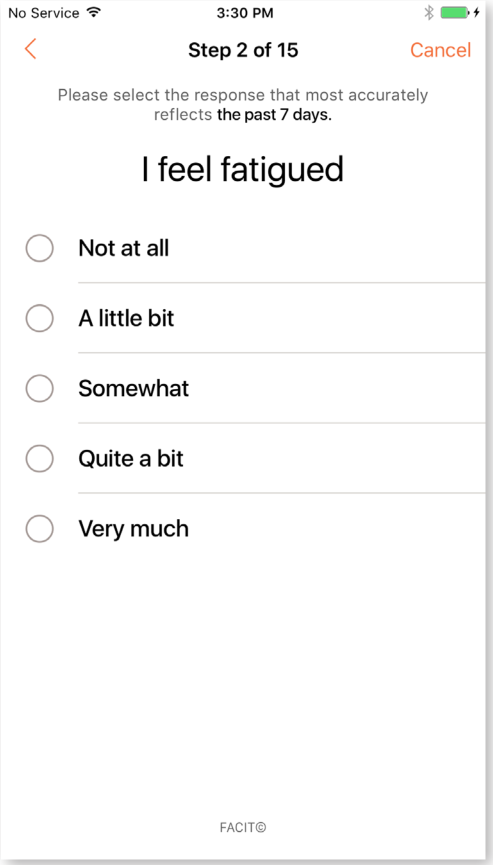

Fatigue Scale (FACIT) 3

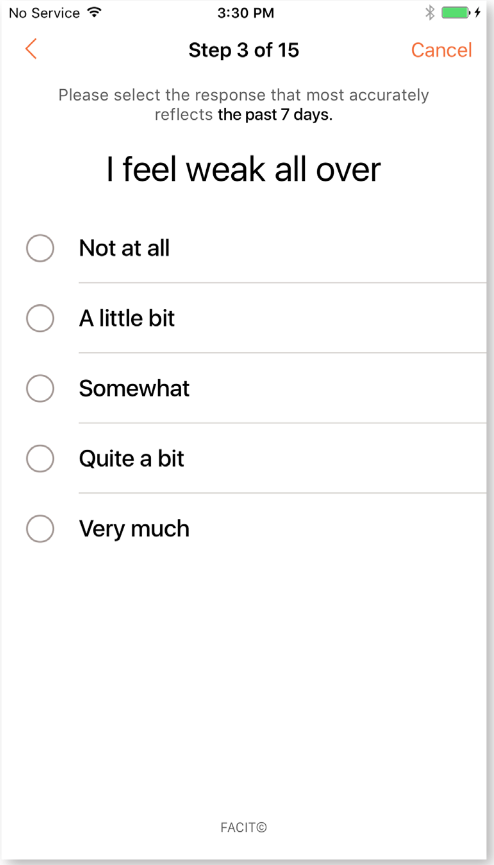

Fatigue Scale (FACIT) 4

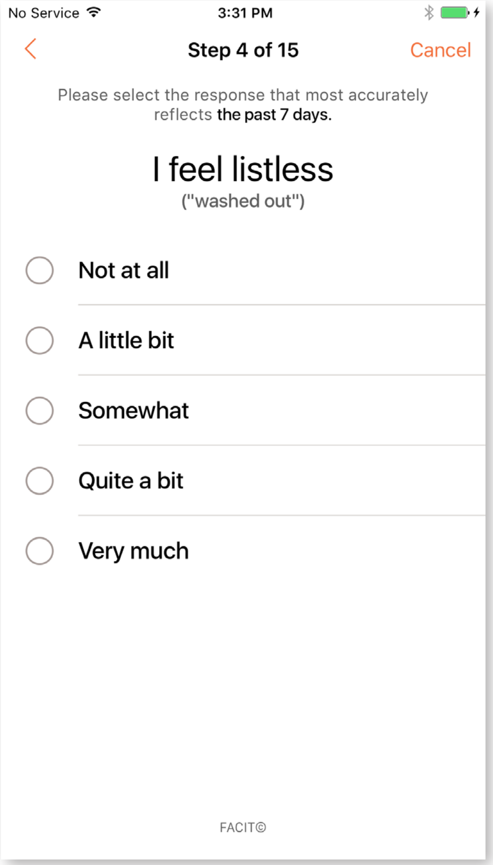

Fatigue Scale Screens, 2

POSSIBLE mobile

Fatigue Scale (FACIT) 5

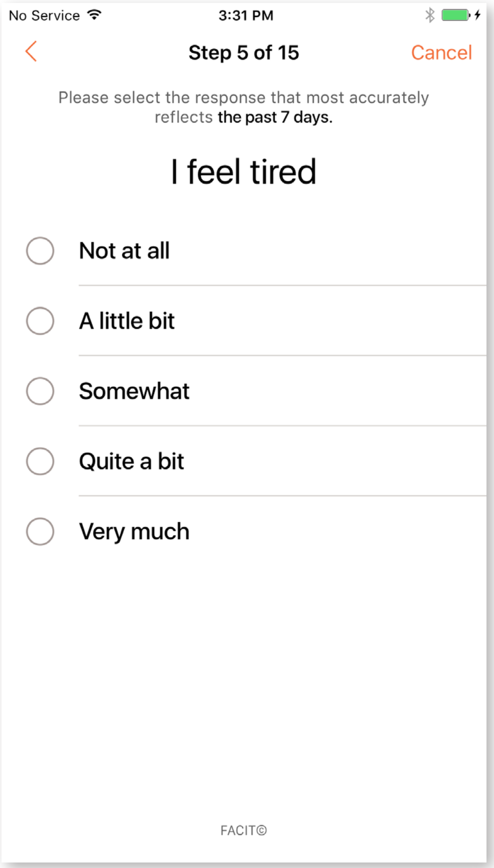

Fatigue Scale (FACIT) 6

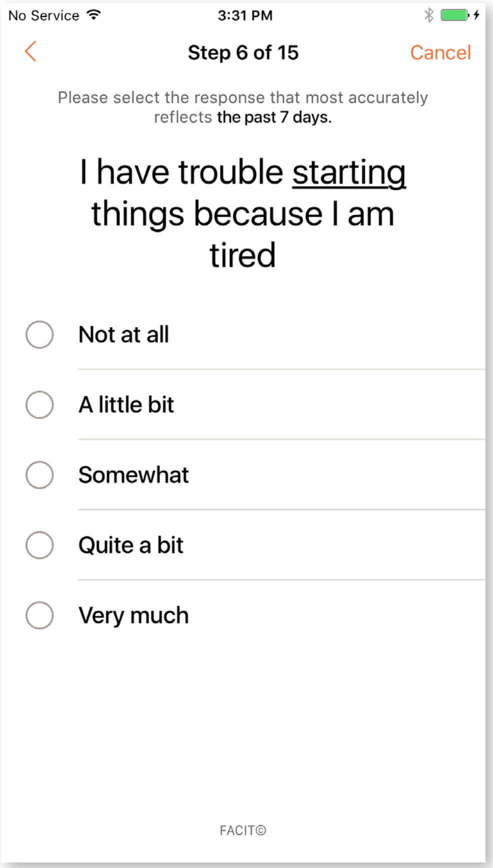

Fatigue Scale (FACIT) 7

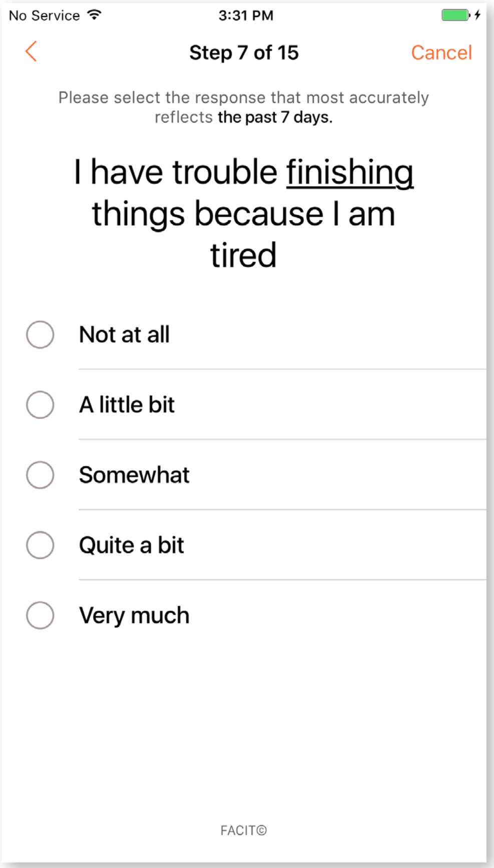

Fatigue Scale (FACIT) 8

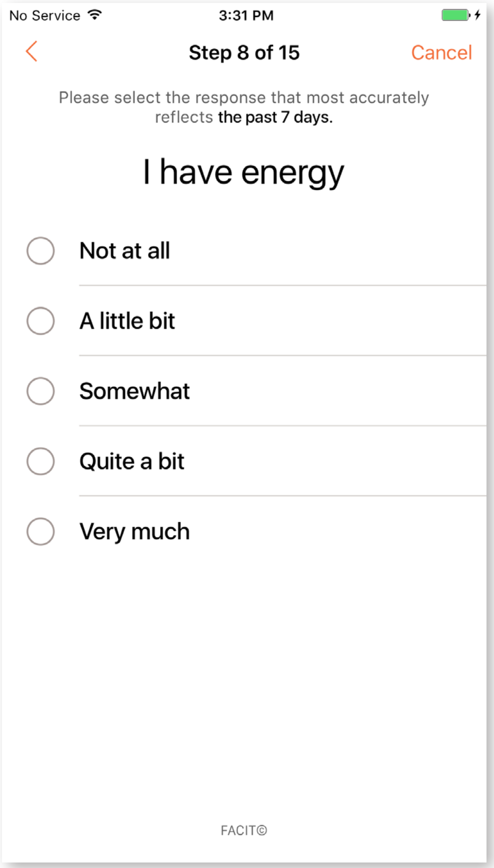

Fatigue Scale Screens, 3

POSSIBLE mobile

Fatigue Scale (FACIT) 9

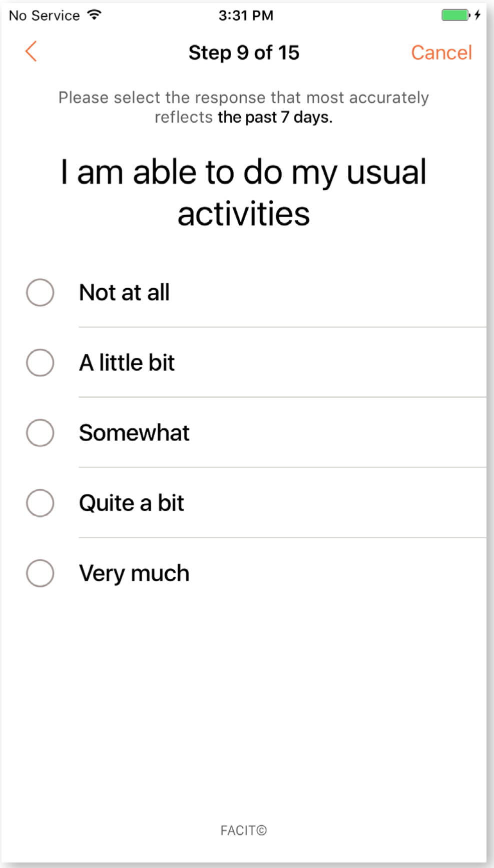

Fatigue Scale (FACIT) 10

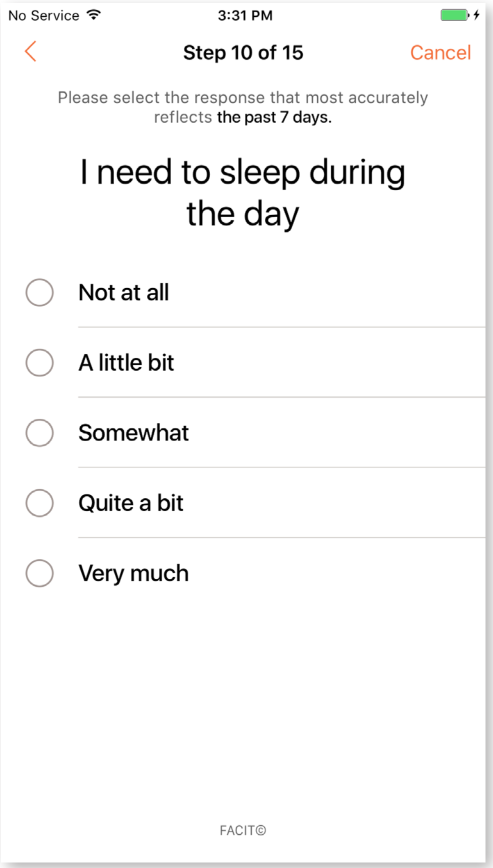

Fatigue Scale (FACIT) 11

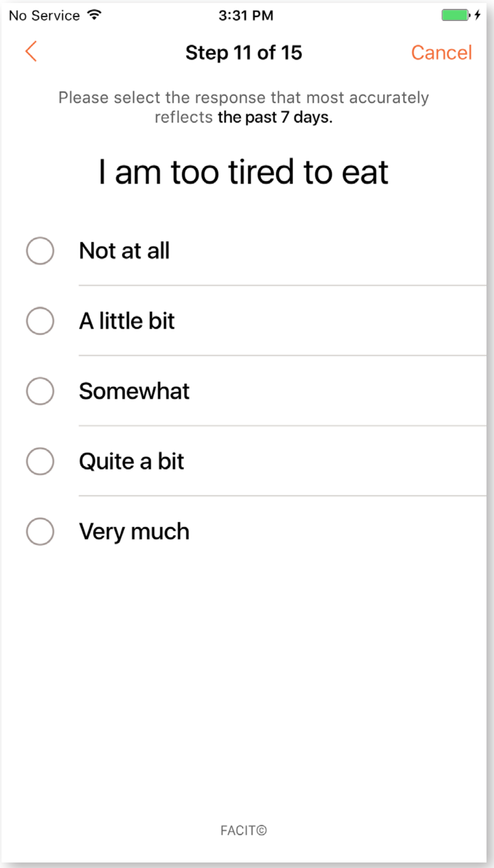

Fatigue Scale (FACIT) 12

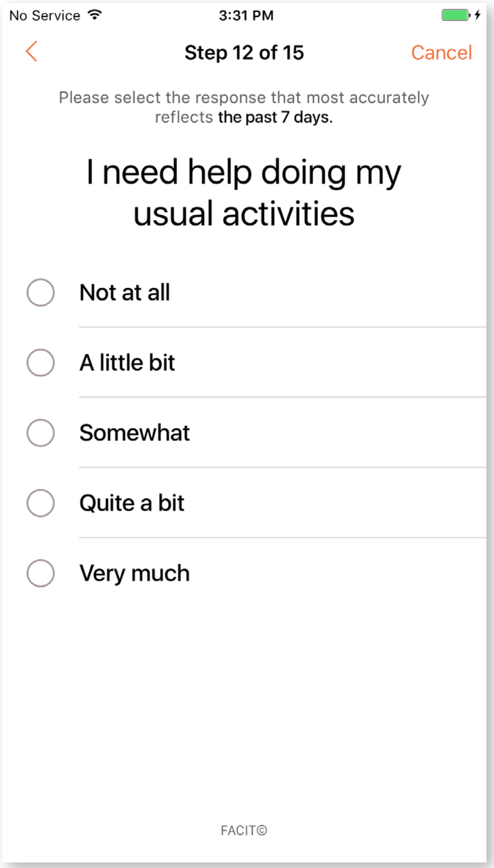

Fatigue Scale Screens, 4

POSSIBLE mobile

Fatigue Scale (FACIT) 13

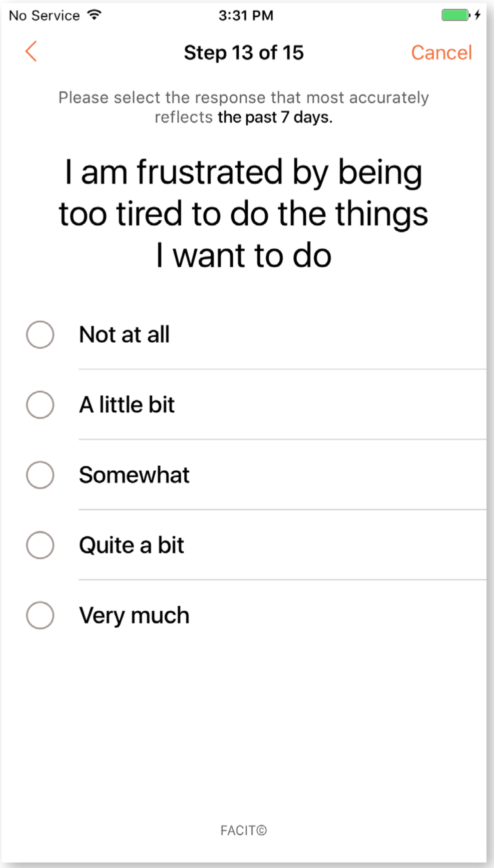

Fatigue Scale (FACIT) 14

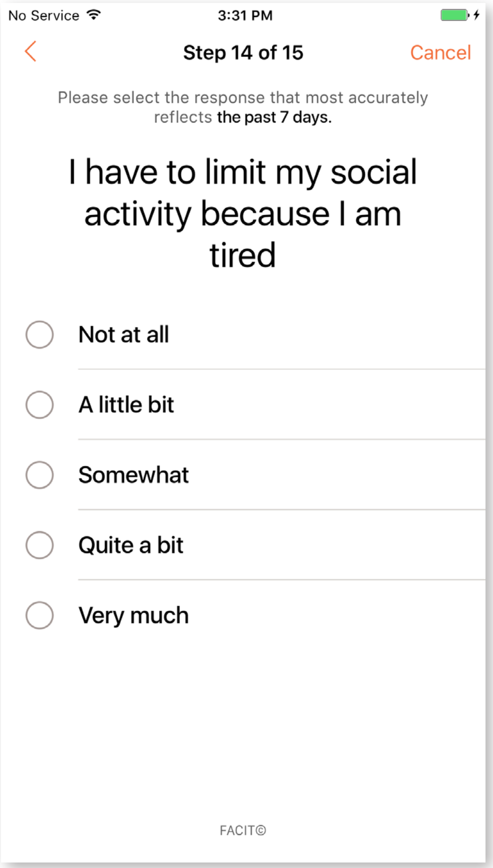

Fatigue Scale (FACIT) 15

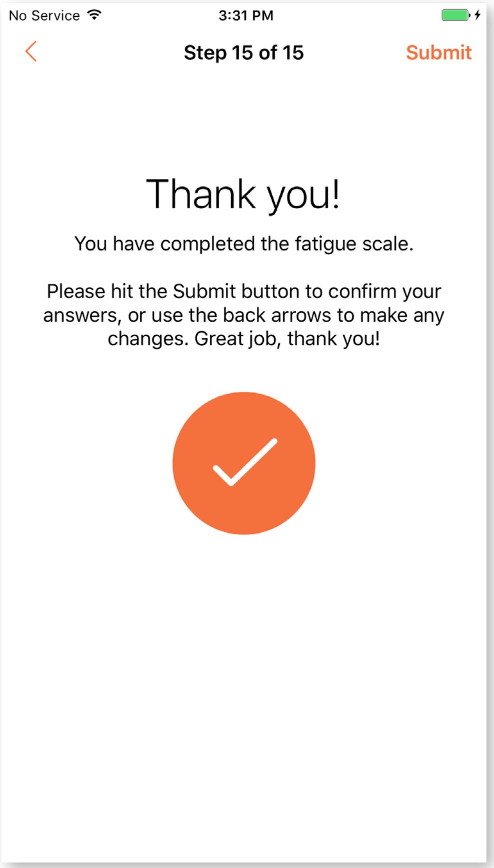

Joint Pain Map Screens

POSSIBLE mobile

Joint Pain Map 1

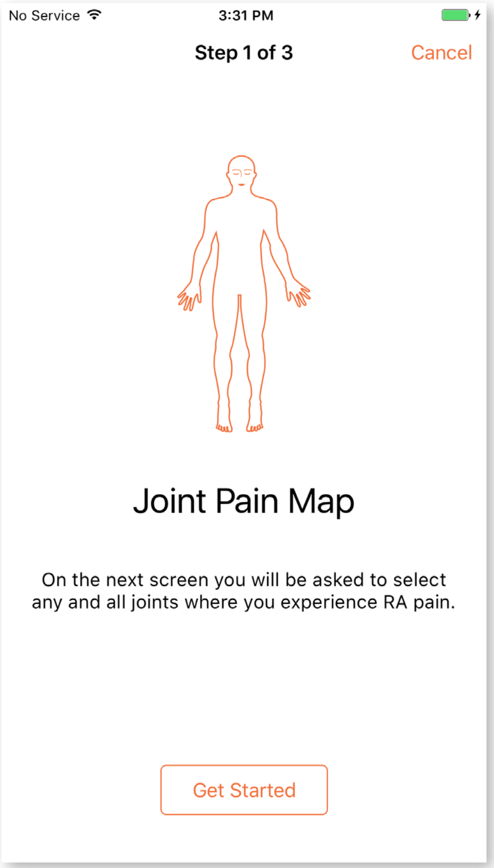

Joint Pain Map: More Info

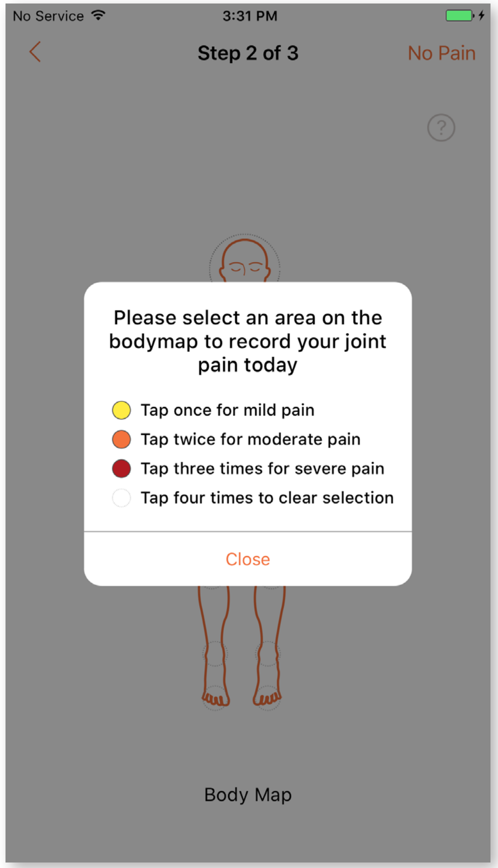

Joint Pain Map 2

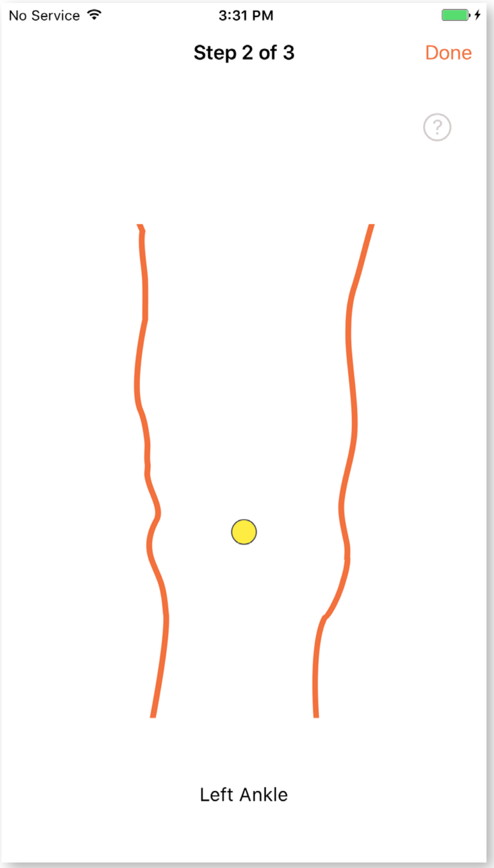

Joint Pain Map 3

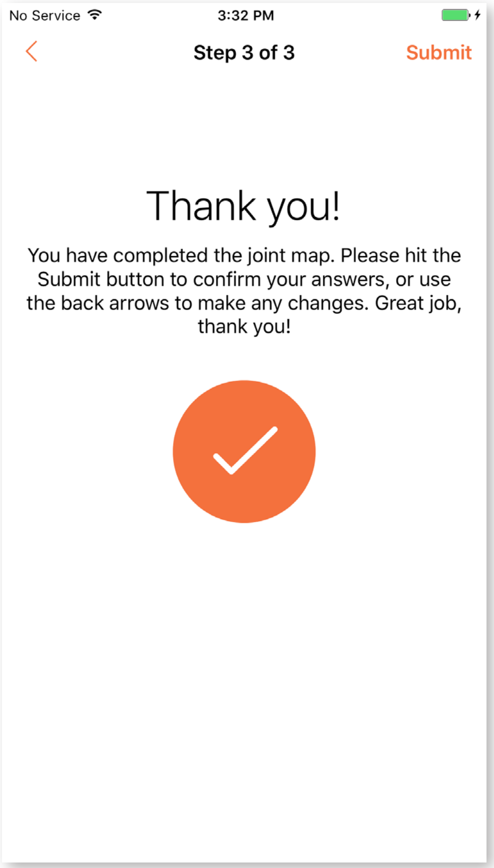

Wrist Exercise Screens

POSSIBLE mobile

Wrist Exercise 1

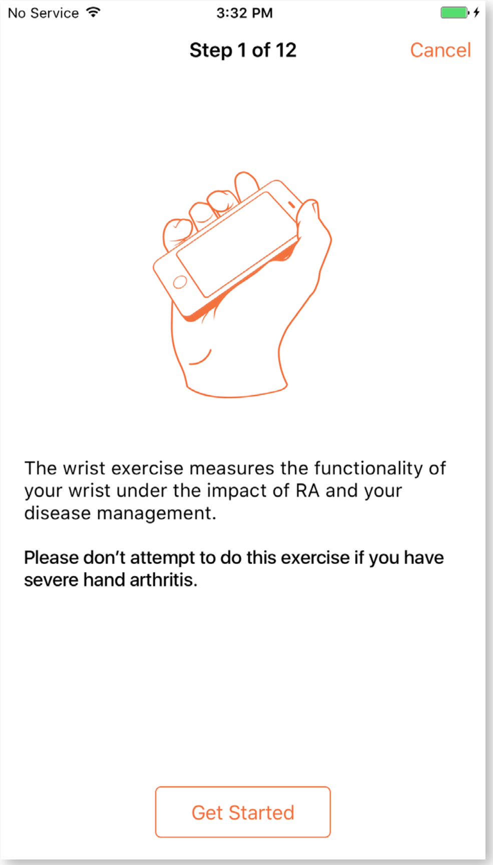

Wrist Exercise: GIF Tutorial 1

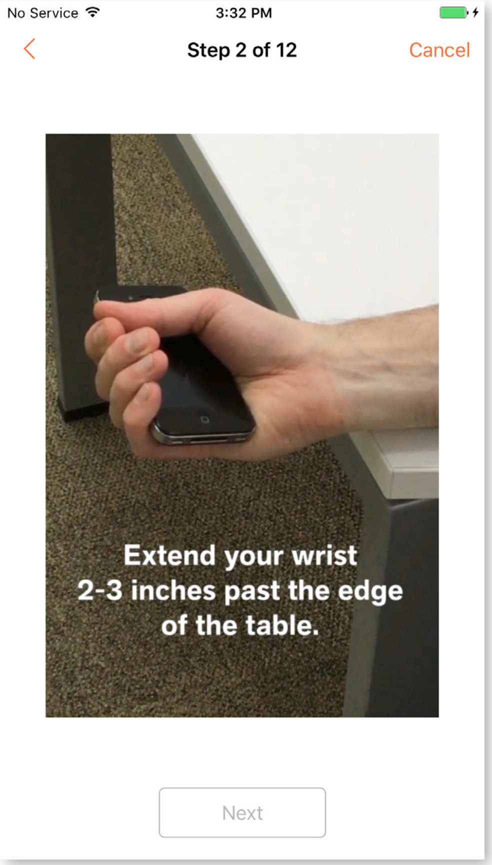

Wrist Exercise: GIF Tutorial 2

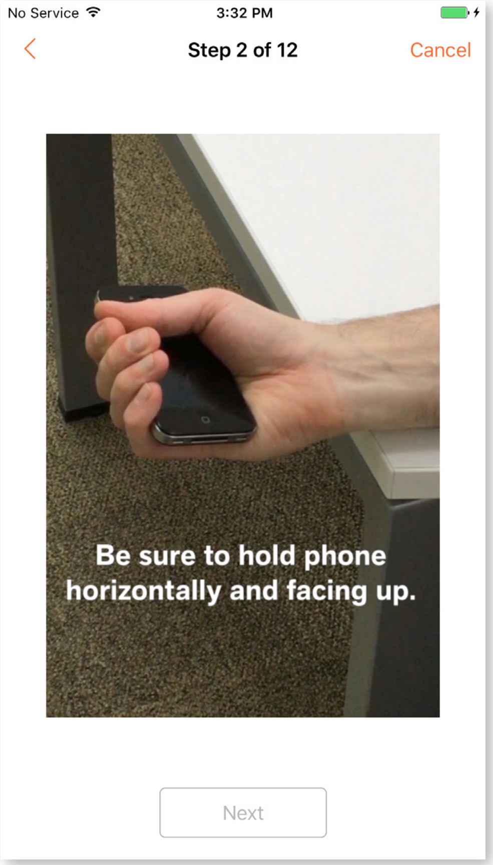

Wrist Exercise: GIF Tutorial 3

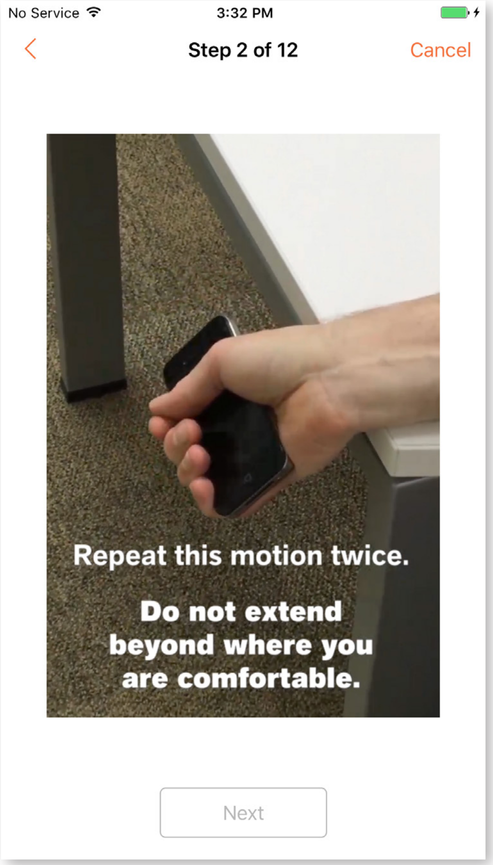

Wrist Exercise Screens, 2

POSSIBLE mobile

Wrist Exercise: GIF Tutorial 4

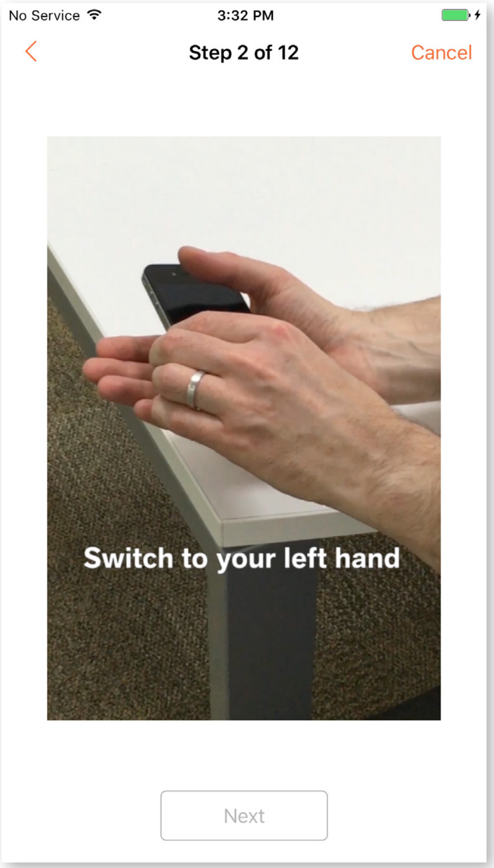

Wrist Exercise 3

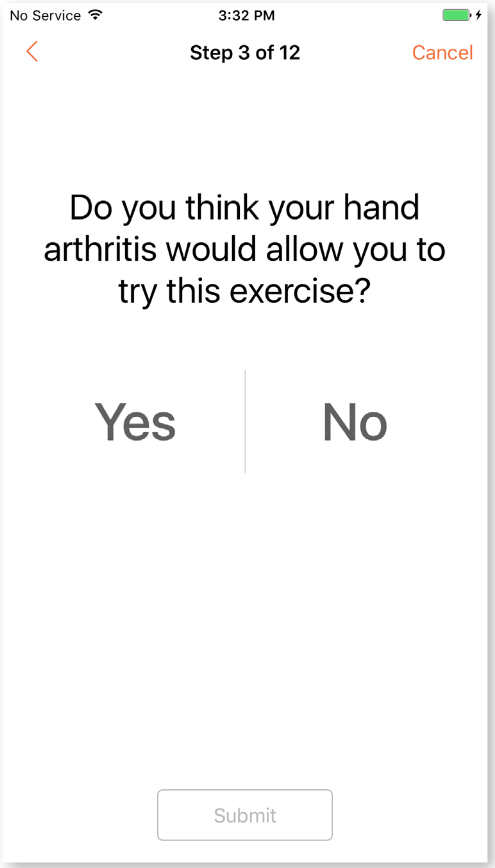

Wrist Exercise: Do Not Need To Complete

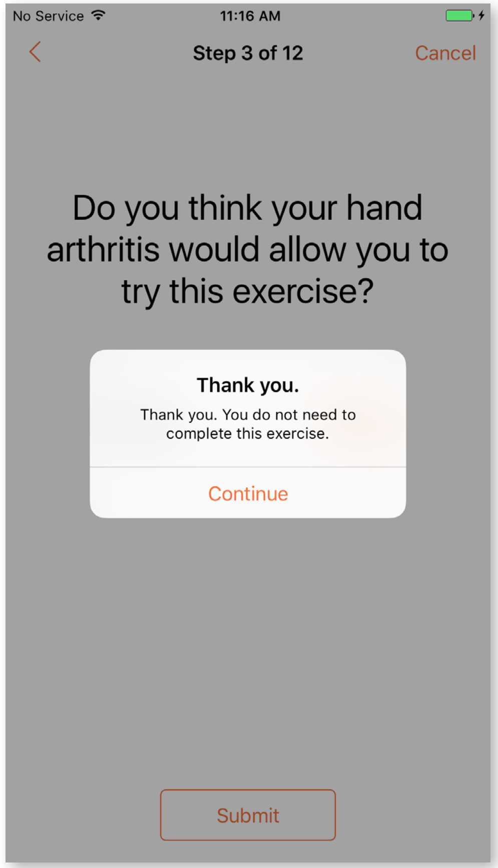

Wrist Exercise 4

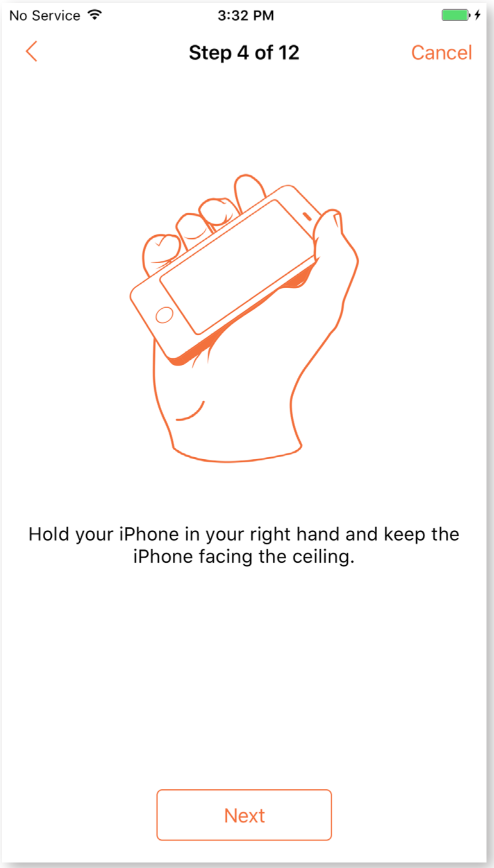

Wrist Exercise Screens, 3

Wrist Exercise 5

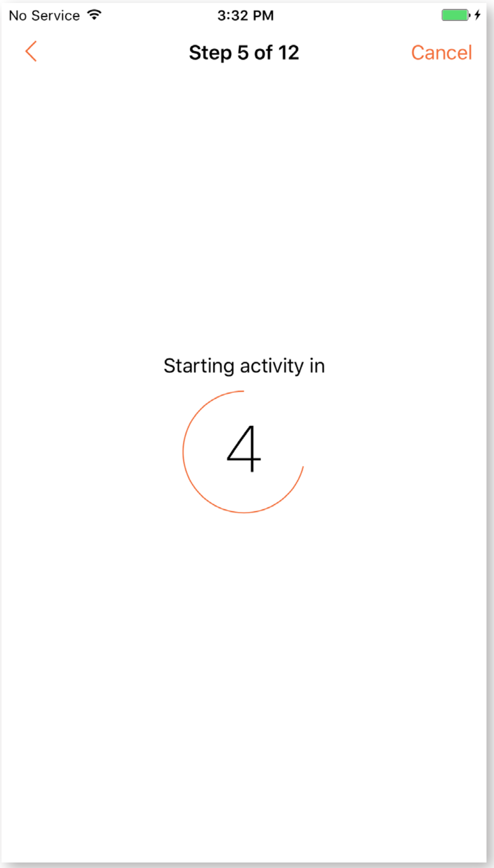

Wrist Exercise 6

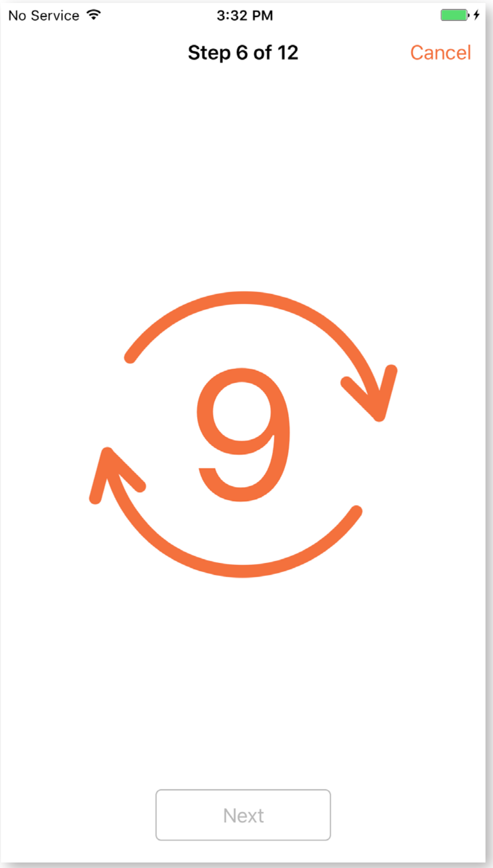

Wrist Exercise 7

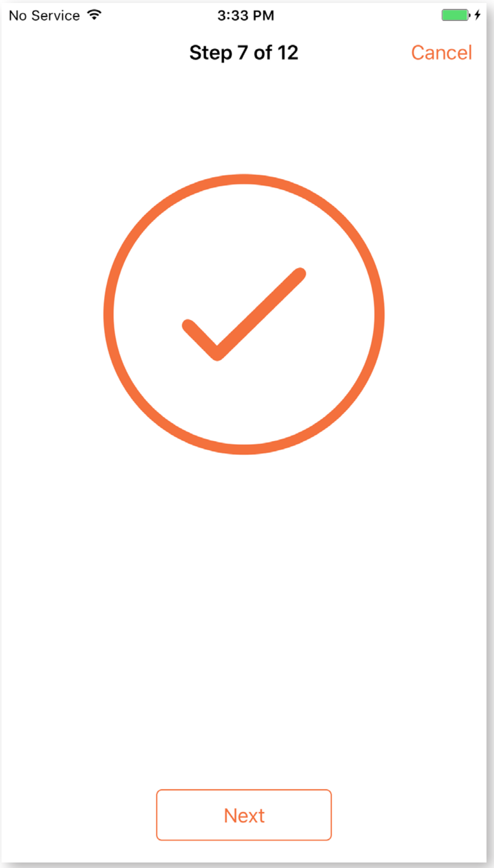

Wrist Exercise 8

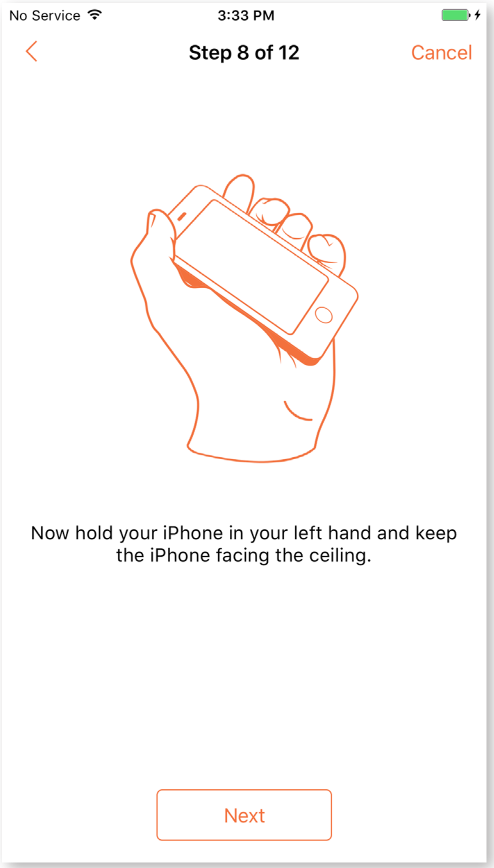

Wrist Exercise Screens, 4

POSSIBLE mobile

Wrist Exercise 9

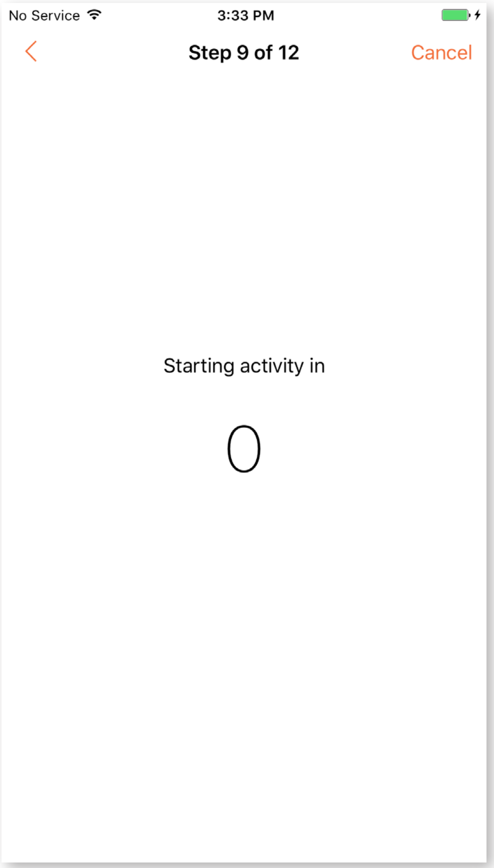

Wrist Exercise 10

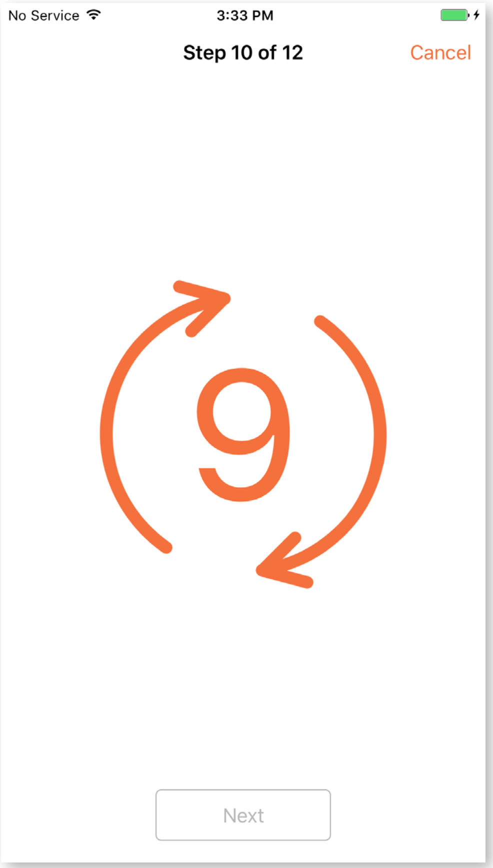

Wrist Exercise 11

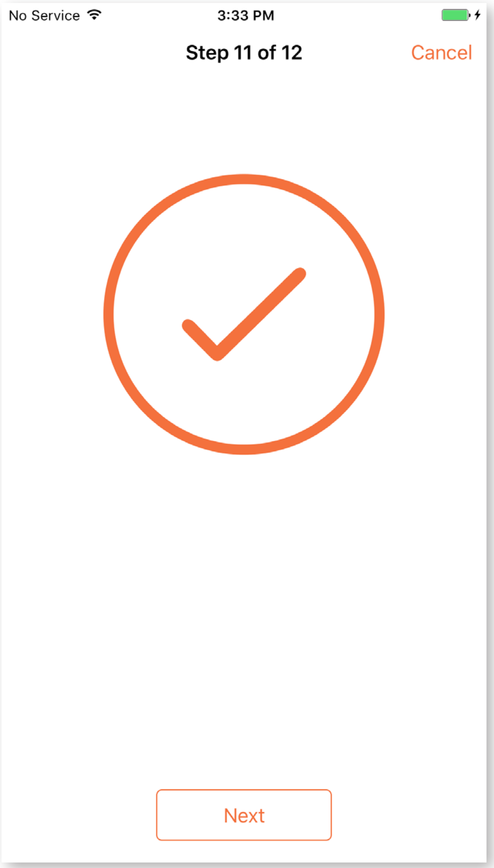

Wrist Exercise 12

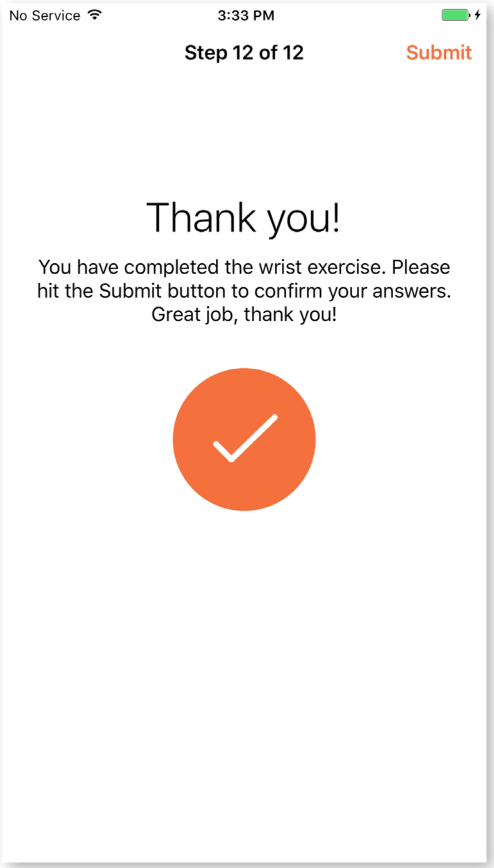

End of Study Screens

POSSIBLE mobile

End of Study 1

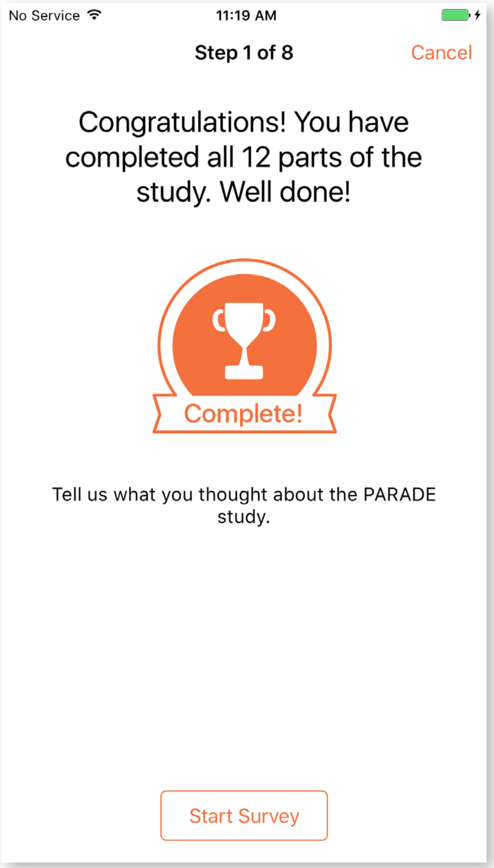

End of Study 2

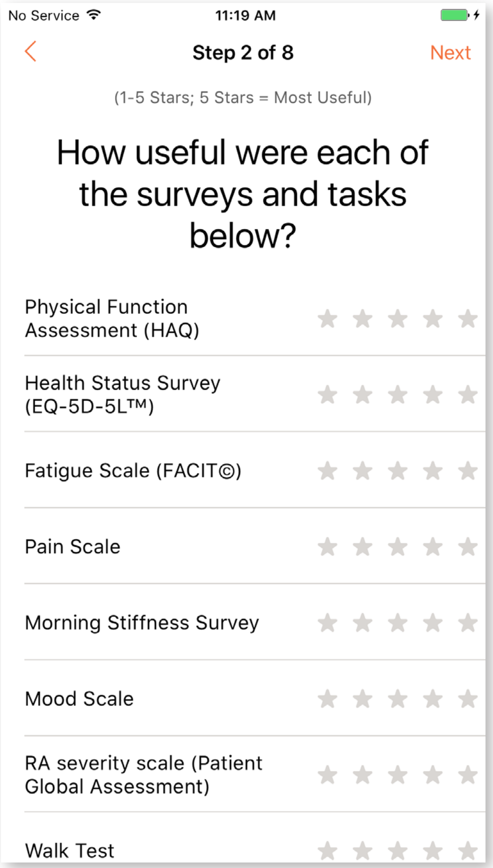

End of Study 2 (cont.)

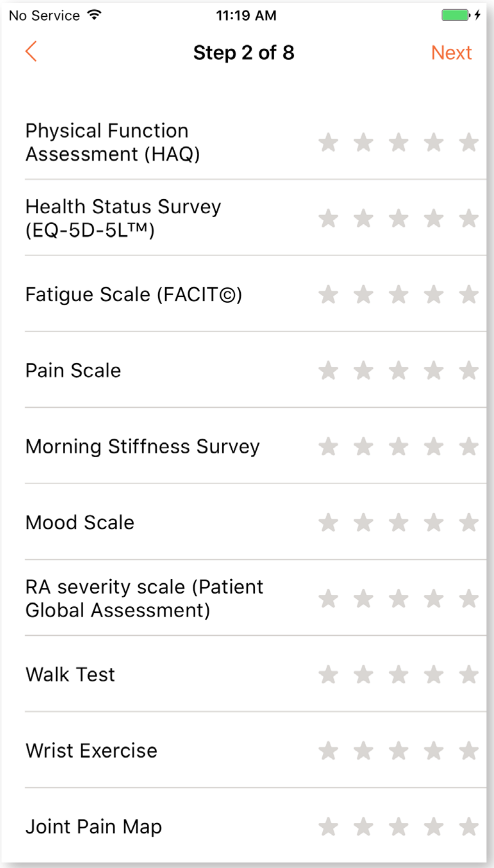

End of Study 3

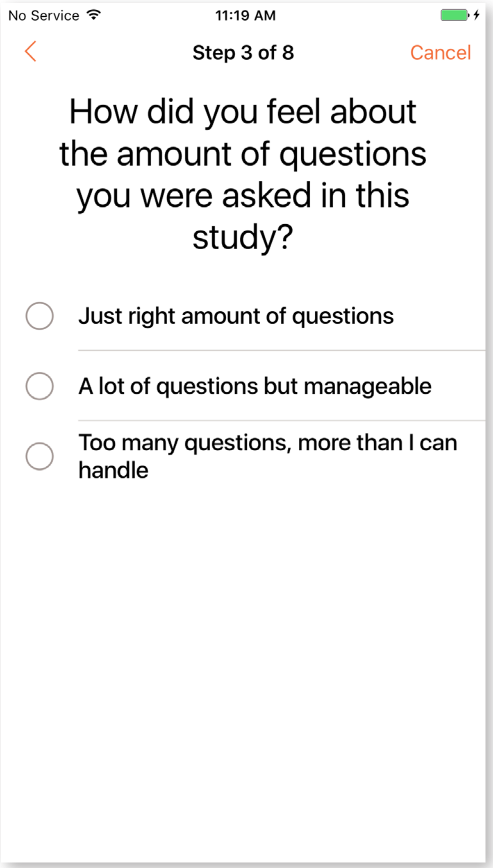

End of Study Screens, 2

End of Study 4

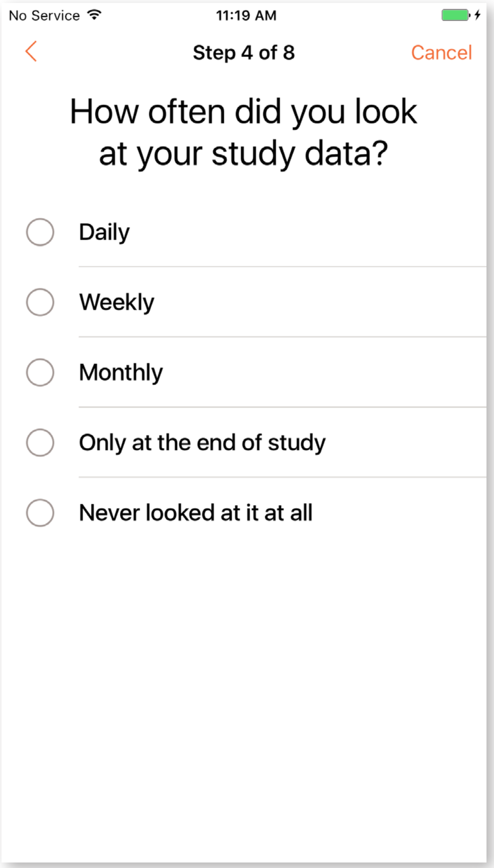

End of Study 5

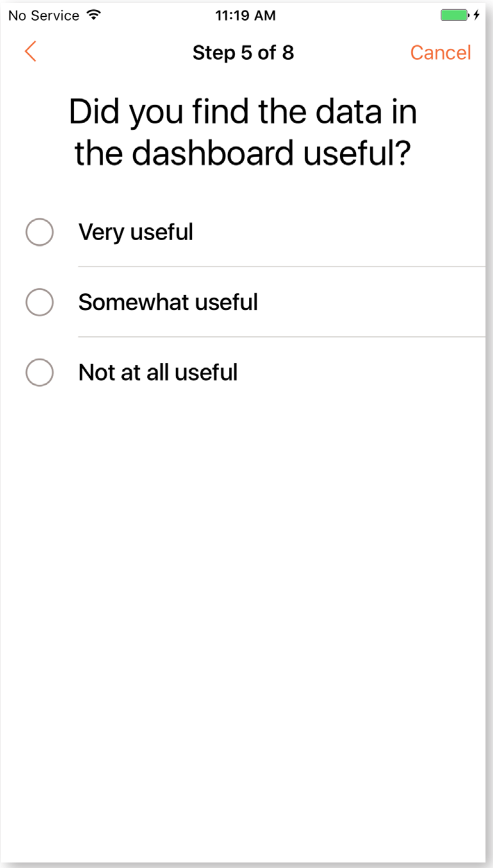

End of Study 6

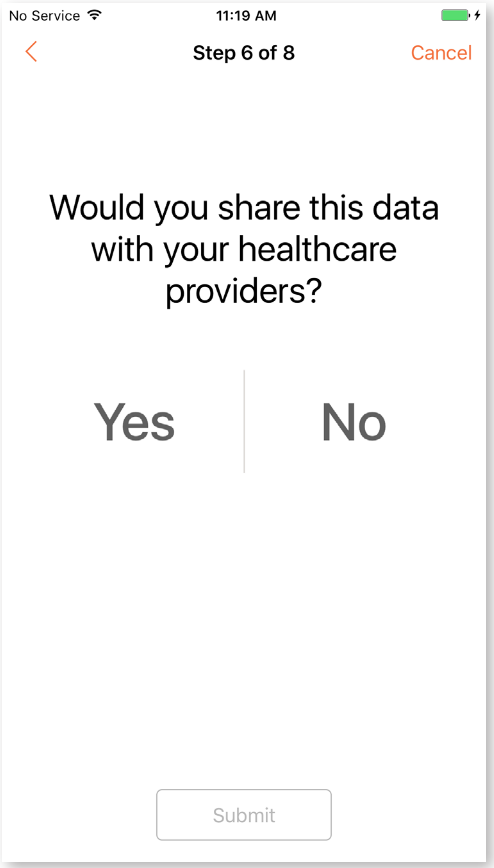

End of Study 7

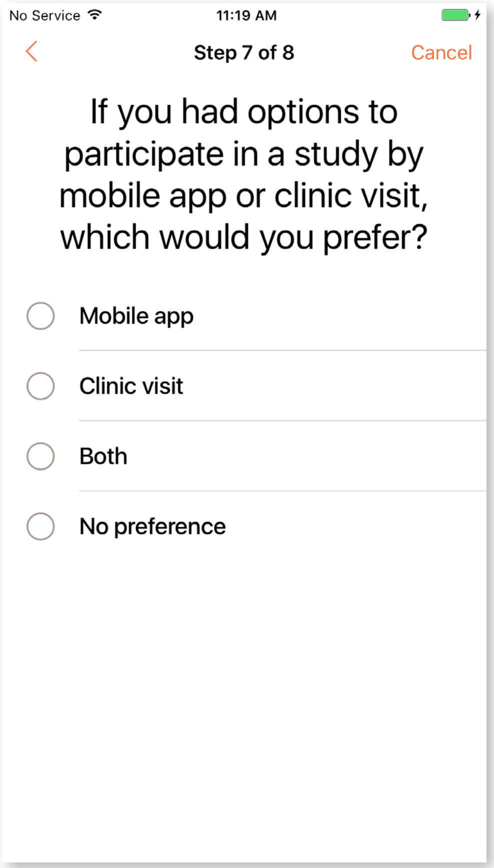

End of Study Screens, 3

POSSIBLE mobile

End of Study 8

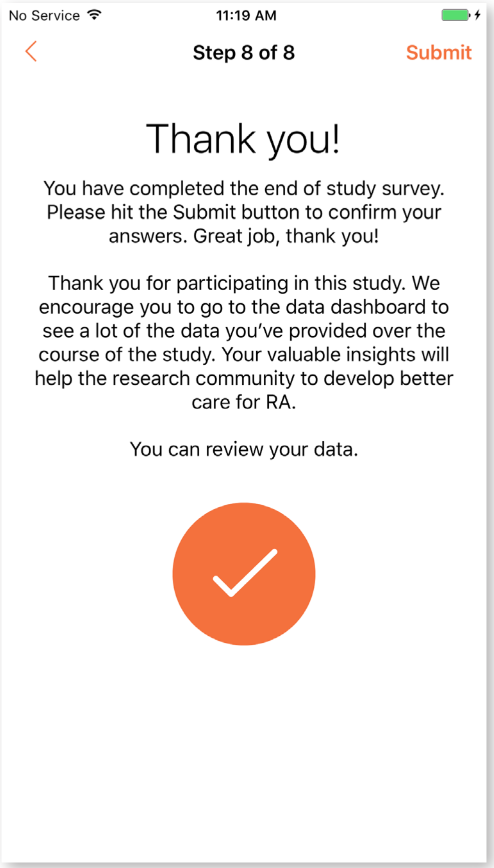

Thank You!

For questions please contact:

Brianne Bell
Project Manager
brianne.bell@possible.com

POSSIBLE mobile
