# Supplementary material for: Using a ResearchKit Smartphone App to Collect Rheumatoid Arthritis Symptoms From Real-World Participants: Feasibility Study
Source: JMIR Mhealth Uhealth. 2018 Sep 13;6(9):e177. doi: 10.2196/mhealth.9656 (PMC6231853; doi:10.2196/mhealth.9656)
Supplement: Multimedia Appendix 2 [file mhealth_v6i9e177_app2.pdf]

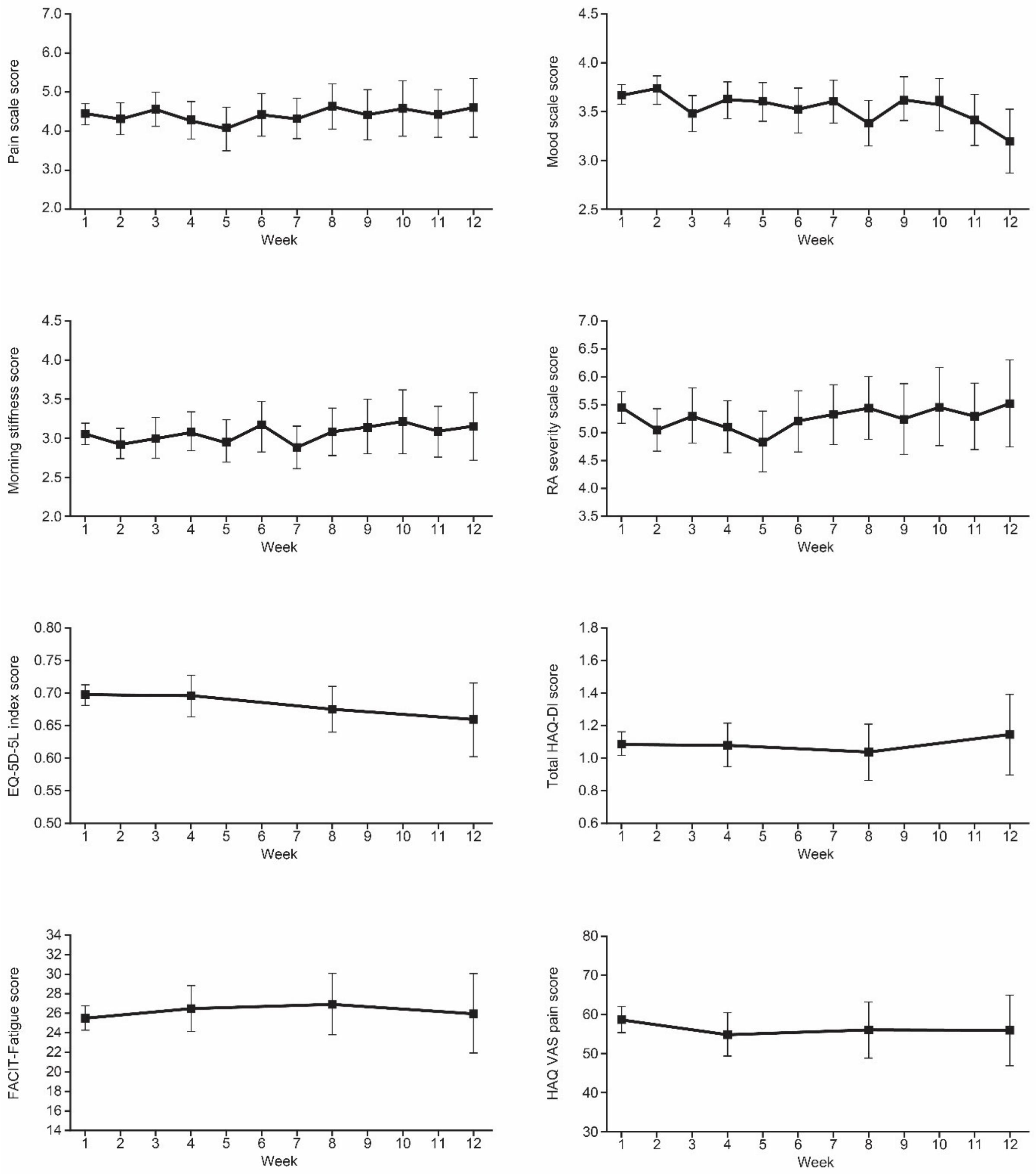

Multimedia Appendix 2. Mean patient-reported outcome responses
EQ-5D-5L, 5-level version of the Euro Quality of Life, 5-dimensions; FACIT, Functional Assessment of Chronic Illness Therapy; HAQ-DI, Health Assessment Questionnaire-Disability Index; HAQ-VAS, Health Assessment Questionnaire-Visual Analog Scale; RA, rheumatoid arthritis
